# An Artificial Intelligence-Assisted Umbrella Review of Eating Disorder Meta-Analyses (2020–2025): Mapping Treatment Efficacy, Comorbidity, and Persistent Gaps in Sample Diversity

**DOI:** 10.3390/nu18213650

**Published:** 2026-11-03

**Authors:** E. Caitlin Lloyd, Pablo Del Sol Calderon, Cate Morales, Euna Lee, Sarita Maharajh, Brooke Moorman, Daniel Le Grange, Kelsey Hagan, Sasha Gorrell

**Affiliations:** 1 Department of Psychiatry, Columbia University, New York City, NY 10032, USA; 2 New York State Psychiatric Institute, New York City, NY 10032, USA; 3 Department of Psychiatry, Universidad Complutense, 28040 Madrid, Spain; 4 Department of Psychiatry, Virginia Commonwealth University, Richmond, VA 23284, USA; 5 Williams College, Williamstown, MA 01267, USA; 6 Department of Psychiatry and Behavioral Sciences, University of California, San Francisco, CA 94143, USA

**Keywords:** anorexia nervosa, artificial intelligence, BERT, bulimia nervosa, binge eating disorder, Claude, eating disorder treatment, eating behavior, generative artificial intelligence, meta-analysis

## Abstract

**Background/Objectives:** Eating disorders (EDs) are serious psychiatric illnesses that result in physical and mental-health impairments. A unifying summary of the broad collection of ED research findings is lacking. This umbrella review leveraged artificial intelligence (AI) to summarize ED-focused meta-analyses, to identify directions for future research and treatment. **Methods:** PubMed, PsycINFO, Web of Science, Embase, and ProQuest were searched for meta-analyses of human ED research published between 1 June 2020 and 31 May 2025 (inclusive). A large language model (LLM) conducted risk of bias assessment and data extraction for studies meeting all inclusion criteria. Neural topic modeling (BERTopic) identified the themes present across included meta-analyses, and an LLM summarized findings of articles in each category. **Results:** 220 meta-analyses were included. AI tools demonstrated good accuracy in quality appraisal, data extraction, and data synthesis (97%, 93%, and 96%, respectively). Most meta-analyses had at least one critical flaw (per AMSTAR 2 quality appraisal). Neural topic modeling identified 16 themes within the set of included articles: Neurocognition, Treatment Effectiveness, Intervention Efficacy, Biomarkers, Premorbid Conditions, Clinical Features, Epidemiology, Psychiatric Comorbidity, Behavioral Addictions, Long-Term Outcomes, Prevention, Psychological Factors, Treatment Gaps, COVID-19, Pharmacotherapy, and Avoidant Restrictive Food Intake Disorder. Article summaries collectively identified ED prevalence of 1.69% and associations of EDs with mortality and disability. Studies covered a range of illness mechanisms and treatments, though disproportionately included Western females. **Conclusions:** This review highlights the broad scope but limited generalizability of ED research, as well as the potential of AI to accelerate systematic review workflows.

## 1. Introduction

Eating disorders (EDs) are serious forms of psychopathology, characterized by a persistent disturbance of eating behavior and food intake resulting in considerable impairment, and physical and mental health comorbidity. EDs have one of the highest mortality rates of all mental illnesses [1,2], and lack consistently effective treatment [3]. Over the past four years, incidence rates of EDs have escalated, with data suggesting dramatic increases in onset in Western countries (i.e., as high as 30–40%; [4]); prevalence rates have also been increasing globally in recent years [5,6]. Considering the increasing incidence of EDs and the seriousness of these illnesses, it is essential to disseminate up-to-date knowledge among clinicians and researchers alike to mitigate harm and costs to patients, their families, and society. To address this need, the current study implemented an umbrella review of ED research conducted in the past five years—a window likely to represent the most current research advances and foci in a fast-moving literature base [7].

Systematic reviews and meta-analyses are often considered to be positioned at the top of the evidence hierarchy, to be key drivers of evidence-based practice, and to address knowledge gaps concerning the mechanisms of and treatments for EDs. Despite their utility, systematic reviews and meta-analyses are limited by the quantity, quality, and comparability of available evidence; when including small and poorly conducted research, or highly heterogeneous findings, their utility decreases [8]. Furthermore, the combination of reviews on similar topics and idiosyncratic decisions by authors (e.g., inclusion criteria) contributes to conflicting or misleading findings among reviews of apparently the same literature [9]. For example two reviews concerning the efficacy of pharmacological treatment for anorexia nervosa [AN] reached different conclusions about the effects of anti-psychotic medication on body mass index (BMI) [10,11]. In addition, although existing reviews collectively cover a broad area, individual studies often focus on specific topics and populations. This collection of issues precludes a rapid, holistic summary of the latest evidence to guide research and clinical practice.

An umbrella review combines evidence across systematic reviews and meta-analyses and weights conclusions by the quality of the investigation from which they are drawn, providing the opportunity to contextualize effect sizes and mitigate errant assumptions that might arise from considering a single meta-analytic review in isolation. There is, however, a significant burden associated with identifying and summarizing extracted data and evaluating the quality of studies, particularly when the scope of the review is large [12]. While prior umbrella reviews of eating disorders literature have been conducted, they have focused on specific sub-topics (e.g., prevalence of illness among certain populations, treatment efficacy) within this literature, which again precludes an understanding of the wider literature base.

Advances in artificial intelligence (AI) have provided algorithms to assist with study selection, bias assessment, and data extraction [13]. Initial tools were limited in their ability to understand context and consequently faltered without extensive human oversight and training [14]. More recently developed large language models (LLMs) leverage transformer architectures that enable them to process contextual information when parsing and generating text [15]. This use of contextual information resolves ambiguity, promotes flexibility and has enabled greater automation in conducting systematic reviews. For study screening and selection, LLMs perform better than (the current gold-standard) two independent human reviewers [16]. For data extraction and risk of bias assessment, LLM-based tools are accurate [17], and there is excellent agreement between LLM outputs and human reviewers [16,18,19,20], which is comparable to the agreement of two human reviewers [21,22,23].

AI can also assist with literature synthesis, which is both time-consuming and prone to researcher bias through choices about study categorization, selective reporting, and researcher interpretations. Topic modeling facilitates the identification of topic sets in a selection of studies by examining the occurrence and co-occurrence of specific words across articles. While traditional topic modeling techniques ignore context, developments in neural topic modeling leverage the same transformer architectures of LLMs to encode documents using contextual embeddings. This ensures that the numerical vectors used to determine content similarity incorporate semantic meaning, improving the identification of a set of coherent yet distinct topics present in a series of documents relative to earlier techniques [24,25,26,27]. This approach is implemented by BERTopic (so named because it initially relied on the SBERT, or Sentence-Bidirectional Encoder Representations from Transformers, algorithm for converting sentences into text embeddings; now a multitude of sentence transformers may be used). Several recent large-scale reviews have incorporated BERTopic to yield insights into current research priorities and gaps in knowledge [26,28,29,30,31]. Though (to our knowledge) LLMs have not yet been leveraged for narrative reviews, they have shown promise in summarizing multiple text narratives in scientific research and other settings [32,33,34,35].

With the availability of LLM-based tools able to accelerate the review process, the current article aims to leverage these tools to systematically identify and summarize meta-analyses on EDs published in the last five years. The pace of publication in the ED field is notably brisk [7]. As such, the five-year date range was selected to ensure a summary of the most current and relevant research findings. Our primary aims are to describe the most frequently studied topics and key findings, to highlight points of consensus and divergence, to evaluate the novelty of the information, and to identify future research directions. We also intend this review to serve as a roadmap for the responsible and transparent use of AI in systematic review research within the field of EDs, with direct application to clinical science more broadly. As this is a data-driven investigation, there are not specific hypotheses about the topics present in the literature-base.

## 2. Materials and Methods

This review was conducted in line with PRIOR (Reporting guideline for overviews of reviews of healthcare interventions) Guidelines [36]. The AI extension to the PRISMA [37] reporting guidelines [38] was followed to ensure transparent and comprehensive reporting of the use of AI in this review. The review is not pre-registered; our unchartered use of AI necessitated iteration (e.g., changing prompts for extraction and summarization; using different sentence transformer models within the BERTopic framework) and adaptation (e.g., using different language models). Adhering to a pre-defined strategy would have limited our capacity to optimize the utility of AI tools. To promote transparency, we have detailed each methodological decision and its motivation. We have also retrospectively registered the study (https://osf.io/zavxf, accessed on 27 October 2026), and provide a log of key methodological decisions in the Supplementary Material.

### 2.1. Search Strategy and Selection Criteria

We searched PubMed, PsycINFO, Web of Science, Embase, and ProQuest databases for meta-analyses on ED research in human populations published within the past five years (1 June 2020 to 31 May 2025, inclusive). Inclusion of ProQuest permitted a search of gray literature. Search criteria were developed in consultation with a university librarian (see Supplementary Material for the search terms for each database). An initial search was completed on 9 June 2025 (23 June for ProQuest). An additional search was conducted on 6 July 2025 to identify studies focused on pica and rumination disorder; this search was conducted across all five databases and applied the same restriction on year of publication (details in Supplementary Material).

Papers were included for initial review based on the following criteria: (1) meta-analysis in a population with an ED diagnosis [Anorexia nervosa, bulimia nervosa, binge eating disorder, avoidant/restrictive food intake disorder (ARFID), atypical anorexia nervosa, other specified feeding or eating disorder (OSFED), eating disorder not otherwise specified (EDNOS)] or clinically significant ED psychopathology (providing an ED diagnosis was specified as an outcome), and (2) human subjects research (e.g., non-animal), to ensure findings could be most informative to clinical research with patient populations as well as treatment. Studies not published in English were not excluded to ensure an unbiased representation of the literature base, though none were identified. Meta-analyses focused on food addiction or orthorexia nervosa were not included unless these constructs were examined among individuals with EDs as they are not formal ED diagnoses per the DSM-5. Meta-analyses relating to populations with overweight or obesity (including bariatric surgery) were excluded unless an ED-related outcome was specified. Meta-analyses examining sub-clinical disordered eating, or reviews that combined across EDs and other diagnoses (e.g., psychosis) without reporting outcomes for diagnostic groups separately were also excluded. These exclusions were applied to ensure conclusions were applicable to diagnosed EDs. Studies that included a meta-analysis conducted without an accompanying systematic search (which often occur in genetic and neuroimaging publications) were excluded as this approach does not capture all studies relevant to addressing a specific question. Because the scope of the review was broad, a formal set of Population, Intervention, Comparator, Outcomes, and Study design (PICOS) criteria were not defined. However, for a summary of the inclusion and exclusion criteria applied, see Table 1.

Articles were screened at the title and abstract level. Abstracts that passed this screening were subjected to full-text review. At all levels, papers were screened by two independent human reviewers; a third resolved conflicts. All authors participated in initial and full-text screening. Inter-reviewer reliability was assessed using an average of pairwise Cohen’s κ coefficients for each pair of reviewers.

### 2.2. Quality Appraisal and Data Extraction

Extracted data included article meta-information (title, publication year, author information, study focus, and aims) and methodological information (inclusion and exclusion criteria; number of studies and total participants across included studies; demographic information; main findings and quantitative information for primary outcomes, i.e., effect sizes and confidence intervals). Information for each study (including the bibliographic record and doi) is available in Supplementary Material (Supplementary Tables).

The AMSTAR 2 [39] was used to determine the quality of each included review, and in turn the confidence that should be placed in the result of each review. This tool assesses the quality of a meta-analysis based on reported methods (e.g., pre-registration, comprehensive search strategy, examination of heterogeneity and bias) and differentiates between critical and non-critical flaws.

Data extraction and quality assessment were performed in a single step using the LLM claude-sonnet-4-20250514, available through Anthropic (https://claude.ai/new, accessed on 27 October 2026). Claude was selected due to established performance in these tasks, and superiority compared to alternative LLMs [40]. To conduct extraction and assess risk of bias, a standardized API call was made to the Claude platform to request extraction of data for items specified in a CSV file that accompanied the call (API script and extraction template available here: https://GitHub.com/CaitlinLloyd/UmbrellaReview; API calls made on 2 October 2025). Evaluation of study quality based on AMSTAR 2 was also requested. Note that available protocols and supplements, in addition to the article, were provided to the LLM for the purposes of data extraction and quality appraisal. The prompt was iteratively refined to ensure requested information was returned and reporting was accurate (see Supplementary Material for prompt and details of refinement). The final quality determination based on AMSTAR 2 evaluation was made using guidance from the original publication [39], which describes how confidence in the results of the review should be assigned given the number of critical and non-critical flaws. Quality was downgraded for “No” responses only (i.e., not “Partial Yes” responses), and to varying degrees depending on whether the item was critical or not.

Two human reviewers independently assessed the accuracy of the LLM-based data extraction and quality appraisal for 25 (~11%), a proportion similar to that of prior investigations [19]. The ~11% of articles were selected at random, and disagreements between the two reviewers were resolved by a third reviewer. The threshold for relying on LLMs for extraction across all fields, and for AMSTAR 2 assessment, was set to 90% accuracy. This threshold is higher than what has been reported for human accuracies [41,42] and was implemented to optimize the validity and reliability of the review findings.

Data-extraction accuracy was calculated as the number of fields extracted correctly divided by the total number of fields evaluated. Because the number of outcomes varied across studies, the number of evaluated fields also varied by review. AMSTAR 2 accuracy was assessed separately at the item level by comparing each LLM-generated item rating with the corresponding human assessment.

### 2.3. Data Synthesis

#### 2.3.1. Topic Modeling

Neural topic modeling was implemented using BERTopic (v0.17.3) [43]) which was applied to the set of abstracts from included studies. This technique converts text to numeric vectors (embeddings) that incorporate word meaning using transformer architectures (i.e., the same as those implemented in LLMs) and performs clustering on these vectors. This approach allows texts to be grouped according to shared themes even when distinct terminology is used to describe phenomena, for enhanced clustering performance relative to traditional methods [24,25,26]. The workflow for abstract clustering is based on current recommendations and described in Table 2. Default parameters were used for each stage.

We explored two sentence transformer models (“all-MiniLM-L6-v2” and “PubMedBert-MARCO”) and several cluster sizes (i.e., 7, 10, 13, 16) to optimize the identification of abstract topics. We compared eight different models, and with study team consensus, selected the final model based on topic coherence and diversity, or lack of redundancy in topics [29]. Coherence was calculated using the UMass approach [45], which calculates the pairwise co-occurrence of the top (in this case, 12) words describing a topic (normalized by the number of times each word occurs in isolation) across documents in that category. Here more negative scores indicate improved coherence of documents within a given topic theme. Topic diversity was calculated as the percentage of unique words in the top 10 words describing a theme (i.e., extent to which these words appear in this theme only). Higher scores indicate greater diversity in the collection of topics.

#### 2.3.2. Topic Description

A LLM (claude-sonnet-4-20250514) summarized articles within each category and provided an overview of main findings. We used a two-stage process to generate the article summaries. First (Phase One), a standardized API call with a detailed request for information (i.e., overall label, findings summary, population description, study similarities/differences, key findings from each study) was made for each cluster; in this case the LLM was provided with article TXT files for each article in the category. Texts were broken into chunks when input exceeded the maximum number of characters (100,000). If the collection of articles in a given category exceeded this number articles were processed in multiple batches; an article was never split across batches. In the event that articles were processed in batches, summaries were produced per batch, and then merged by a separate script. In Phase Two, we made another standardized API call requesting a shorter and more focused summary, which prioritized findings that seemed novel. Here, the model was provided with the summary generated in step one. All prompts are provided in the Supplementary Material, along with summaries of how they were refined. Phase One summaries were requested on 2 December 2025; Phase Two summaries were requested on 6 April 2026. The temperature parameter was set to 0.3 in the API calls.

Prompts were constructed free from preconceived notions about information or themes to reduce bias from researcher expectations. Quality of Phase One summaries was evaluated on 3 (~20%) of the clusters (per current standards; [19]), using the following criteria: (1) Appropriate cluster label; (2) Summary emphasis on the most relevant information; (3) Identified subthemes are present; (4) Data and conclusions extracted are accurate; (5) Relevant information is presented; (6) Summary of convergent and divergent findings is accurate. If errors were present (either for data from a single study or conclusions from the set of studies), the category as a class failed, given the implications for validity of results.

One human reviewer (PD) carefully scrutinized the final summaries from Phase Two to ensure the data, conclusions, and references cited were correct. Four reviewers (ECL, PDS, KH, SG) reviewed the Phase Two summaries in their entirety and leveraged their pre-existing knowledge and experience to check the technical and clinical accuracy and interpretability of the summaries. This team collectively possesses a broad methodological and clinical expertise, promoting greater confidence in the ability to detect inaccuracies in the summaries. The presented summaries were adjusted to correct grammatical errors, ensure consistent use of abbreviations, and detail the number of included studies. Occasionally a more material adjustment was made (e.g., if a sentence was completely misaligned with the rest of the cluster description); this is detailed in full in the Supplementary Material (Supplementary Tables). The original full Phase One and Two summaries are available as Supplemental Files.

**Figure 1:**
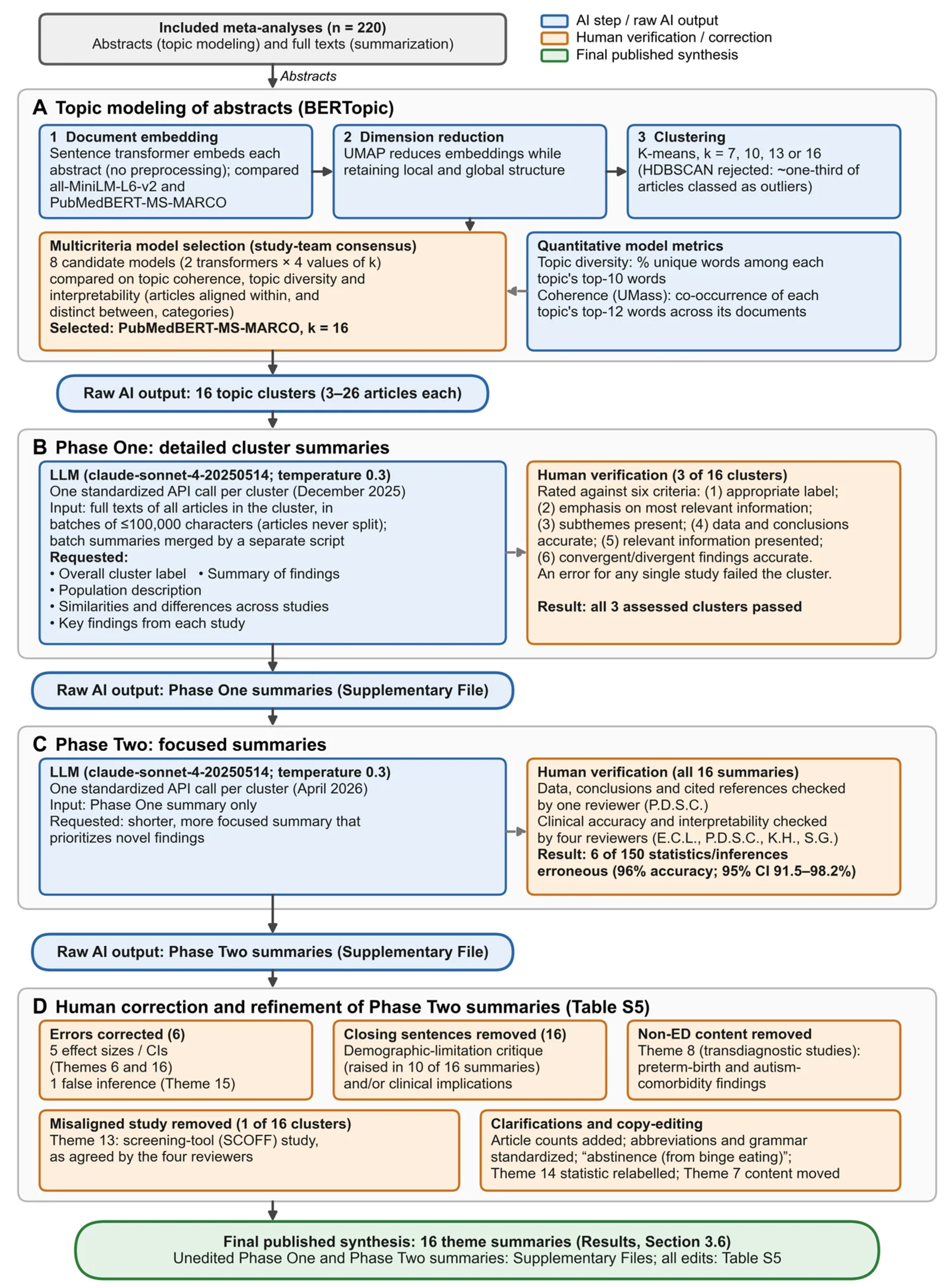
Workflow for topic modeling and summarization of article categories for included studies.

All scripts used for this analysis were written in Python, and are available here: https://GitHub.com/CaitlinLloyd/UmbrellaReview. The workflow for the topic modeling and topic cluster summary procedures is presented in Figure 1 (which differentiates between human and AI tasks).

#### 2.3.3. Confidence in Review Findings

The broad scope of this review meant that providing an overall rating for confidence as per established criteria like GRADE would be infeasible without being somewhat misleading or challenging to interpret. Instead, we consider the distribution of AMSTAR 2 findings within each cluster to guide the confidence in the main findings of the review. AMSTAR 2 ratings should not be interpreted as indicative of the certainty of evidence for any one of the range of outcomes covered by the studies included in this review.

## 3. Results

**Figure 2:**
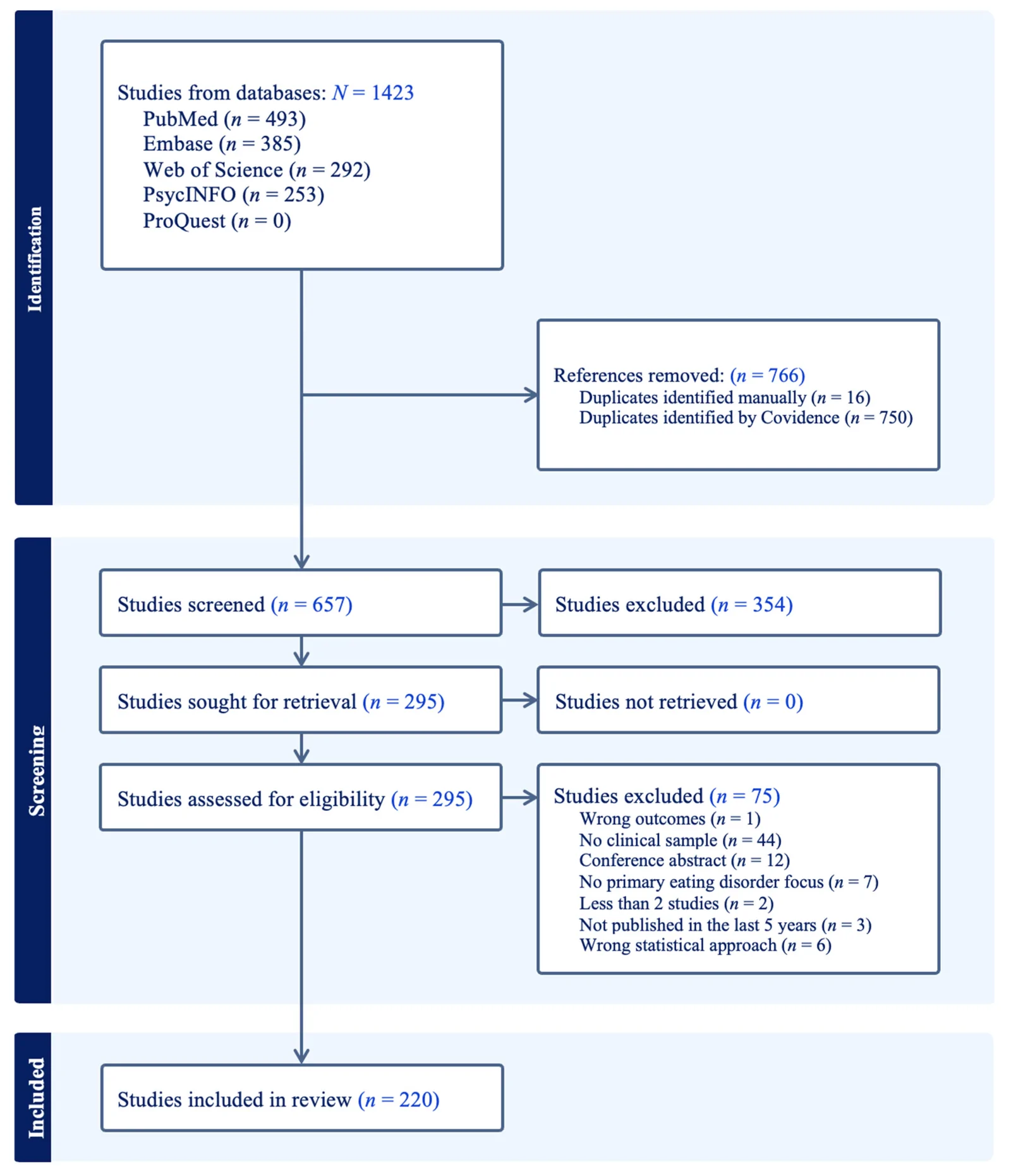
PRISMA Flowchart of included studies.

A PRISMA Flowchart is presented in Figure 2. A total of 1423 studies met initial criteria for review; the ProQuest search returned no eligible studies. After removing 766 duplicates, 657 studies proceeded to title and abstract screening. Of these, 295 studies progressed to full-text screening; 220 were retained for final inclusion. Extracted data from the 220 studies are included in the Supplementary Tables excel file. Inter-reviewer agreement was 82.9% during title/abstract screening and 86.7% during full-text screening.

### 3.1. Quality of Data Extraction

Across the 25 reviews (of 220, ~11%) included in the data extraction validation sample, both human reviewers evaluated 1075 individual data-extraction fields. A total of 998 fields were accurate, yielding an overall accuracy of 92.8% (95% CI: 91.1, 94.2%). Accuracy varied by extracted field: it was 90.2% for the number of studies, 96.7% for sample size, 94.6% for effect estimates, and 91.3% for confidence intervals.

AMSTAR 2 accuracy was 96.6% (95% CI: 94.3, 98.0%; out of 384 fields, 371 were correct). Accuracy remained above 95% for both critical and non-critical AMSTAR 2 domains (Supplementary Table S7).

### 3.2. Quality Assessment

Most (*n* = 137) included meta-analyses had more than one critical flaw, per AMSTAR 2 assessment. Consequently, modal ratings across almost all clusters were “critically low”. The most frequent violation of a critical item was for item 13 (“*Did the review authors account for RoB in individual studies when interpreting/discussing the results of the review?*”, *n* = 209). For details about individual meta-analyses, see the Supplementary Material (Table S6).

### 3.3. Topic Modeling

The performance of the eight BERTopic model variants did not differ substantially (see Supplementary Material, Table S4). The 16-cluster solution (“PubMedBert-MS-MARCO”) was selected for performing the best across our specified criteria: topic coherence and diversity, and interpretability of the cluster solution (articles in each category aligned and were clearly distinct from each other, and the rationale for the distinct categories was clear). An overview of the emergent clusters is provided in Table 3.

**Figure 3:**
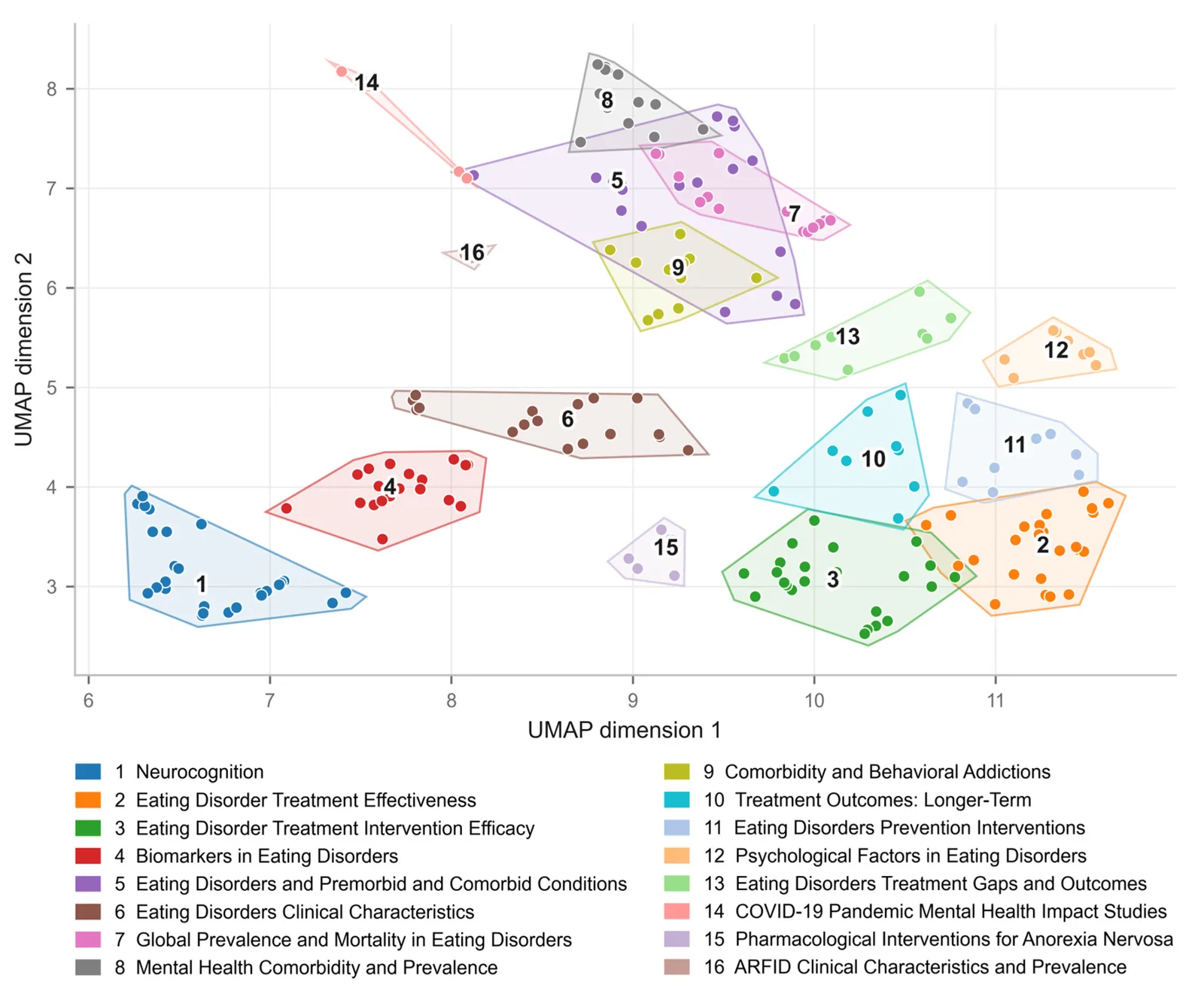
Abstract positions in semantic space Each point represents one article’s abstract, positioned using UMAP, which projects the high-dimensional text embeddings onto a reduced number of dimensions (here 2) while preserving local similarity. The axes have no intrinsic meaning or units; articles that are closer together have more semantically similar abstracts.

Dimension reduction (UMAP) was used to project the study embeddings onto two dimensions for visualization (see Figure 3).

### 3.4. Quality of Data Summarization

All of the three assessed clusters passed quality assessment according to the six criteria described for Phase One, suggesting that in this limited validation sample accuracy was acceptable. Six erroneous inferences/statistics, out of a total of 150 provided, were identified in the Phase Two summaries (resulting in 96% accuracy; 95% CI: [91.5%, 98.2%]). Five of these were effect sizes or confidence intervals, and the remaining one was a false inference. Specific details are available in the Supplementary Material (Table S5).

### 3.5. Adaption of Summaries

The LLM noted in 10 of the 16 summaries that ED research is limited by the underrepresentation of males, minoritized populations, and non-Western cultures, and recommended that these populations be included in future research. We removed text specific to this critique from themes to reduce redundancy. In Theme 8, which included transdiagnostic studies, we removed information that did not apply to EDs. Clusters generally categorized conceptually similar studies; however, on one occasion (out of 16 clusters), certain studies did not align (as agreed by the four reviewers examining the summaries). Specifically, Theme 13 largely centered around longer-term treatment outcomes, though one study focused on screening tool performance. The sentences describing the misaligned study was removed for interpretability (original summaries are presented as Supplementary Material; see Original Phase Two Summaries File).

### 3.6. Emergent Themes

Summaries of article clusters are provided below.

Theme 1. Neurocognitive Mechanisms in EDs

This cluster of 26 articles reveals consistent evidence that EDs involve profound neurocognitive dysfunction across multiple domains, with the most robust findings including moderate-to-large executive function deficits (particularly cognitive flexibility and inhibitory control), widespread gray matter reductions most pronounced in AN (Cohen’s *d* up to −0.95; [67], and severe interoceptive impairments across all ED subtypes with large effect sizes (AN *d* = 1.57, bulimia nervosa [BN] *d* = 2.61, binge-eating disorder [BED] *d* = 1.96; [49]). Neuroimaging meta-analyses consistently identify limbic-prefrontal circuit alterations with distinct patterns between disorders—AN showing reward system hypoactivation while BN demonstrates hyperactivation. Evidence suggests these deficits are partially reversible with treatment, indicating both state and trait components.

Theme 2. ED Treatment Intervention Effectiveness

This cluster of 24 articles on ED treatment research reveals several robust findings. Cognitive Behavioral Therapy (CBT) demonstrates consistently strong effectiveness across diverse populations and settings, with large effect sizes ranging from 0.88 in controlled trials to 1.12 in routine clinical care, achieving 36% abstinence (from binge eating) rates compared to 10% in controls [75,87]. Family-based treatments show superior weight outcomes compared to individual therapy (*g* = 0.46) for adolescents, with separated parent-focused approaches outperforming conjoint formats [73]. Digital interventions emerge as a promising modality, demonstrating moderate effectiveness (*g* = 0.58) with self-monitoring identified as a critical behavior change technique present in all effective digital interventions [78,90].

Theme 3. ED Treatment Interventions

This cluster of 23 articles reveals emerging evidence for diversified ED interventions beyond traditional psychotherapy approaches. Pharmacological treatments demonstrate notable efficacy, with lisdexamfetamine showing large effect sizes for BED (SMD 0.93), while GLP-1 agonists and combination naltrexone/bupropion therapy show promise for weight-related outcomes. Neurostimulation interventions, particularly deep brain stimulation for treatment-refractory AN, demonstrate substantial effects (*g* = 1.13), challenging assumptions about intervention hierarchies that traditionally favor CBT as first-line treatment.

Theme 4. ED Biomarkers and Pathophysiology

This cluster of 19 articles reveals consistent pathophysiological patterns in EDs, with robust evidence for hormonal dysregulation including decreased leptin (SMD −1.62 to −3.84), elevated ghrelin (SMD 2.51), and increased cortisol across measurement methods, alongside reduced growth factors BDNF and IGF-1, elevated inflammatory markers TNF-α and IL-1β, and cardiac structural abnormalities with preserved functional capacity. These biomarker changes appear to represent adaptive responses to starvation rather than primary pathology, with genetic associations showing population-specific effects (5-HTR2A polymorphisms significant only in Caucasian populations). Critically, medical instability occurs across weight ranges, with 29–42% of non-underweight adolescents requiring hospitalization, emphasizing that rapid weight loss is more predictive of complications than absolute weight.

Theme 5. EDs and Comorbid Conditions

This cluster of 18 articles reveals consistently elevated ED prevalence across vulnerable populations, with particularly high rates in transgender individuals (17.7%) and those with type 1 diabetes (14.4% increased risk), alongside strong bidirectional relationships between EDs and medical conditions including diabetes, pregnancy complications, and psychiatric comorbidities. The findings demonstrate robust associations between childhood maltreatment and ED development (OR 2.37), with sexual abuse showing the strongest relationship, while medical comorbidities present complex patterns—AN reduces gestational diabetes risk (OR 0.63) but increases low birth weight risk (OR 1.74).

Theme 6. EDs Clinical Characteristics

This cluster of 17 articles reveals that EDs present with multi-system complications requiring comprehensive clinical attention. Sleep disturbances in AN include reduced total sleep time by 32.1 min and persist after weight restoration [156], while dental complications show dramatic increases in erosion across all EDs (OR 15.27) with particularly severe effects in BN (54.4% prevalence) [157]. AN patients also exhibit significantly elevated autism spectrum disorder (ASD) traits across age groups (*g* = 0.88) with 29% scoring above clinical cutoffs [161], and show severe bone mineral density deficits across all skeletal sites [164]. Importantly, other specified feeding or eating disorders (OSFED) demonstrate comparable or even greater severity than full-threshold diagnoses, with atypical AN patients showing higher eating pathology than typical AN patients, challenging traditional diagnostic hierarchies [158,163].

Theme 7. ED Comorbidity and Global Prevalence

This cluster of 14 articles reveals that EDs affect 1.69% of the global population with substantial geographic variation (0.22% in Asian countries vs. 1.89% in Western countries), carry severe mortality risk (standardized mortality ratio = 5.21 for AN), and demonstrate extensive psychiatric comorbidity including non-suicidal self-injury in 40% of patients, suicidal ideation in 51%, and impulse control disorders in 22%. The research consistently shows that binge-purge subtypes (BN and AN-BP) exhibit the highest rates of comorbid conditions such as substance use (alcohol 28%, smoking 30% in BN).

Theme 8. Mental Health Comorbidity and Prevalence

This cluster of 13 articles reveals mental health comorbidities are pervasive across psychiatric conditions, demonstrating that comorbidity is the rule rather than the exception. Familial transmission follows transdiagnostic patterns, with offspring of parents with mental disorders showing 1.5–3.0 times higher risk of developing any mental disorder across 37 significant transdiagnostic associations [197]. The clinical impact is severe, with mental disorders reducing life expectancy by nearly 15 years globally [187], while age-of-onset data reveals that 34.6% of all mental disorders begin before age 14 and 48.4% before age 18 [196], challenging traditional service delivery models that divide care at age 18.

Theme 9. EDs Comorbidity and Behavioral Addictions

This cluster of 11 articles reveals substantial comorbidity between EDs and addictive/compulsive behaviors, with particularly elevated rates in binge-purge presentations. Key findings include alcohol use disorders affecting ~20% of individuals with binge eating [201], food addiction prevalence of 55% in clinical binge-eating populations [206], and impulse control disorders occurring in 22% of ED cases overall [176]. Social determinants significantly influence risk, with food insecurity increasing binge eating odds by 66% [199], while attachment insecurity shows large effect sizes across all EDs [205].

Theme 10. ED Treatment Outcomes

This cluster of 10 articles reveals consistent evidence for the effectiveness of diverse ED treatments, with psychological interventions maintaining 45–53% abstinence (from binge eating) rates at 12 months [212], exercise showing large effect sizes for BED (SMD = −0.94; [217]), and weight-loss interventions actually improving rather than worsening ED symptoms (SMD = −1.47; [219]). Notably, several findings challenge traditional clinical assumptions: illness duration shows no significant association with treatment outcomes (r = 0.05; [216]), compulsory treatment achieves similar effectiveness to voluntary treatment despite more severe presentations [210], and individuals with BED can successfully engage in weight-loss interventions, though with modestly reduced weight loss and higher dropout rates compared to non-BED individuals [211]. The research demonstrates significant long-term improvements across most ED behaviors over 10+ years [218] and reveals that cognitive inefficiencies are similarly present in both AN and BN but not in BED [213], supporting a more optimistic, individualized approach to treatment.

Theme 11. EDs Treatment and Prevention Interventions

This cluster of 10 articles reveals a consistent pattern of effectiveness for various intensity levels and delivery modalities. Low-intensity and self-guided interventions demonstrate equivalent efficacy to high-intensity therapy for ED psychopathology (*g* = −0.13, 95% CI: −0.30, 0.04) while being more accessible, though with lower remission rates [221]. Early treatment response emerges as a robust predictor of outcomes across all EDs (*r* = 0.41, 95% CI: 0.32–0.48) [220], while intensive residential treatments show superior outcomes to other settings with large effect sizes for symptom improvement (*g* = 0.97, 95% CI: 0.85, 1.09) [222]. Prevention programs demonstrate significant effectiveness in reducing ED onset by 38% (IRR = 0.62, 95% CI: 0.44, 0.87), with peer-led and single risk-factor approaches showing particular promise [224,228].

Theme 12. Psychological Risk Factors in EDs

This cluster of nine articles reveals consistent medium-to-large associations between key psychological factors and EDs, with self-disgust (*g* = 1.90) and eating-related shame (*r* = 0.589) showing the strongest effects, followed by social physique anxiety (*r* = 0.51) and self-criticism (*r* = 0.37), while perfectionism demonstrated more moderate but consistent associations across youth and adults (*r* = 0.20–0.33). Self-compassion emerged as a protective factor with medium negative associations (*r* = −0.34) and showed promise as an intervention target (*g* = 0.58), while CBT targeting perfectionism demonstrated efficacy for ED symptoms (*g* = 0.61).

Theme 13. ED Treatment and Recovery Outcomes

This cluster of nine articles reveals critical gaps in ED care, with only 30% of individuals seeking help despite effective treatments being available. The most robust finding is that approximately two-thirds of people with EDs have unmet treatment needs [239], primarily due to denial of severity and perceived inability of others to help [244]. While recovery rates reach 46% overall and improve to 67% with long-term follow-up [247], relapse remains common at 36–37% across disorders, with post-treatment BMI and pre-treatment depression being the strongest predictors [245,246]. Evidence-based treatments like guided self-help and lisdexamfetamine for BED show significant effectiveness.

Theme 14. COVID-19 Impact on EDs

This cluster of eight articles demonstrates that COVID-19 significantly worsened ED symptoms and increased healthcare utilization, with 57–65% of patients experiencing symptom deterioration across studies [249,250,254]. Güzel 2023’s large-scale analysis (*n* = 4,688,559) found increased ED prevalence (OR 1.30) and moderate effect sizes for symptom severity across AN, BN, and BED (*d* = 0.24–0.29) [248], while another study [251] reported a 54% increase in pediatric ED healthcare utilization (risk ratio = 1.54), with disproportionate impacts on adolescent girls. However, a longitudinal before-after analysis found no significant objective symptom changes (Standardized mean difference = 0.44 [95% CI: −1.30, 2.18]), suggesting a discrepancy between perceived and measured deterioration [255].

Theme 15. Pharmacological Interventions for AN

This cluster of six articles reveals that pharmacological interventions for AN show limited efficacy, with olanzapine emerging as the only medication with modest but significant benefits for weight gain in adults (BMI increase of 0.67 kg/m^2^) but not adolescents [10]. Despite evidence of significant oxytocin dysregulation in both subtypes of AN [257], intranasal oxytocin supplementation failed to improve social cognition or food intake in AN patients [256,258]. Traditional psychotropic medications, including antidepressants and other antipsychotics, demonstrated no significant effects on weight recovery or psychiatric symptoms [11], though dropout rates for second-generation antipsychotics remain acceptable at 28%, primarily driven by personal choice rather than adverse effects [259].

Theme 16. ARFID Clinical Characteristics and Prevalence

This cluster of three articles establishes ARFID as a clinically significant ED with a prevalence of 4.51%, 95% CI: 0.7–10.68% [261]. Despite similar BMI z-scores to AN patients, ARFID patients demonstrate distinct physiological profiles including significantly higher heart rates (WMD = 12.93 bpm, 95% CI: 8.65–17.21) and physical health complications across the entire weight spectrum [260]. The strong comorbidity with ASD, with autism prevalence of 16.27% (95% CI: 8.64–28.53%) in ARFID populations compared to ~1% in the general population [262], highlights the need for cross-condition screening protocols.

### 3.7. Confidence in Findings of the Review

Given AMSTAR 2 confidence ratings for each cluster were generally low, the certainty of evidence provided by the body of research within this review should be considered low (though note that the evidence for individual studies or specific outcomes may be stronger; see the Supplementary Tables for effect sizes and confidence intervals for each included meta-analysis). Clusters 11, 12 and 16 had a relatively smaller number of studies with critical flaws, so somewhat higher trust may be placed in the cluster summaries pertaining to eating disorders prevention, psychological factors in eating disorders and ARFID.

## 4. Discussion

In this umbrella review, we leveraged AI tools to synthesize meta-analytic findings within the ED field with the goals of (1) generating up-to-date knowledge for clinician-scientists, and (2) providing a framework for responsible and transparent use of AI tools to accelerate the process of conducting such a review. Broadly, this AI-based approach to review is the first of its kind in this mental health subfield with high applicability to other domains. Our methods identified 16 distinct themes across 220 included reviews.

### 4.1. Synthesis of Current Research

Two general findings emerged from the umbrella review. First was the lack of diversity (with respect to sex, gender, race, and socioeconomic status) in ED research, and the need to improve reporting and inclusion of these characteristics. Consequently, the generalizability of findings from existing research to all individuals experiencing EDs is a significant concern. A second overarching finding was evidence of transdiagnostic features between EDs and other psychiatric conditions (e.g., ASD, substance use disorders [200,262]). This suggests a need to include ED screening across settings (e.g., educational, primary care) and to consider interventions targeting transdiagnostic processes. Notably, calls for broader screening sit in tension with the U.S. Preventive Services Task Force’s conclusion that current evidence is insufficient to assess the balance of benefits and harms of screening adolescents and adults without signs or symptoms of an ED [241], particularly those in non-underweight bodies. The diversity and transdiagnostic findings identified here suggest that this evidence gap may partly reflect the absence of appropriate assessment tools, rather than evidence that screening is not needed. While the included meta-analyses cannot necessarily be considered independent sources of evidence (given likely overlap in the included primary studies), these conclusions were supported by enough studies to be determined reliable.

The 16 themes identified by topic modeling broadly encompassed ED treatment or phenomenology. Treatment research collectively reinforced established knowledge such as lack of evidence for effectiveness of pharmacological treatment for restrictive EDs [10], the importance of evidence-based psychotherapies for EDs, and the association of early response to treatment and better prognosis [220]. Two qualifications are warranted. First, the pharmacological picture is not uniformly negative: olanzapine has shown modest benefit for weight gain in adults with AN [263], and lisdexamfetamine is approved for BED. Second, the existence of evidence-based psychotherapies does not ensure their delivery; a persistent research-to-practice gap means many patients do not receive these treatments with fidelity [264]. Our review also revealed several novel findings specific to ED interventions. For instance, although outcomes from less intensive (e.g., e-Health; self-guided) and more intensive (e.g., residential treatment) care are less examined (relative to standard outpatient care), more recent work suggests substantial benefit from both in decreasing the incidence of EDs, or reducing ED severity, respectively [222,226]. This work also suggests that a staged care model matched to the individual may be most effective in mitigating the full spectrum of illness observed across populations. It is noteworthy that the studies producing these conclusions were generally of higher quality (per AMSTAR 2 assessment) relative to other meta-analyses included in the review. Several studies failed to find a significant association between illness duration and treatment outcomes, e.g., [216]. These results support continued advocacy for engagement in treatment despite longstanding illness, contrasting with recent (and highly controversial) calls to shift toward a palliative approach for individuals with a long and severe illness [265]. This debate, catalyzed by proposed criteria for “terminal anorexia nervosa” [266] and vigorous rebuttals [190], merits careful framing. A null group-level association between illness duration and outcome does not rule out individual differences in treatment response, and longer-duration patients who enroll in treatment studies may be selectively those most willing to engage; note that the field still lacks a consensus definition of severe and enduring AN [267]. Our findings argue for prospective research on prognostic indicators, and for adding harm-reduction and quality-of-life outcomes alongside, not in place of, recovery-oriented goals. Finally, the recent increase in the implementation of neuromodulation for other forms of psychopathology has been extended to AN, though this work is in its infancy [97].

Regarding phenomenological research, EDs have been identified as among the deadliest psychiatric disorders, and research confirms this grim reality (AN mortality odds = 5.2, all other EDs = 3.4; [2]. Several studies (of relatively higher quality in terms of AMSTAR 2 assessment, compared to those focused on other eating disorders) investigated ARFID, and outcomes suggest that this diagnosis is even more common than previously known (4.5%) with particularly elevated overlap with ASD [261,262]. Although AN and some presentations of ARFID (e.g., fear of aversive consequences) are both characterized by significant weight loss, medical consequences (e.g., cardiovascular outcomes) appear to be different. Relatedly, being underweight in the context of constitutional thinness has a distinct physiological signature compared to AN. Collectively, these findings indicate that the rate and duration of weight loss (distinguishing ARFID from AN) and percent body fat (distinguishing constitutional thinness from AN) drive the severity of medical consequences of AN. On the other hand, atypical AN, a diagnosis with increasing recognition since its mention in the DSM in 2013 [268], has severe medical implications, with up to 42% of patients demonstrating medical instability despite a “normal” weight [163]. These findings challenge the weight-centric structure of current diagnostic criteria for AN without consensus on a definition of “significant weight loss”, underscore calls to reconsider whether the distinction between “typical” and “atypical” AN is clinically meaningful [269], and highlight how weight stigma may delay recognition and treatment of restrictive EDs in larger bodies. Future work investigating the physiological and psychological consequences of weight loss in atypical AN appears warranted.

The present review also identified macro-level environmental influences on ED trajectories. One class of findings emphasized the impact of the COVID-19 pandemic. Cross-sectional studies largely suggested the considerable impact of this pandemic on increasing prevalence rates and ED severity, as well as comorbidities, e.g., [248]. However, longitudinal studies suggest the opposite, showing few changes in ED symptoms, e.g., [255]. Possible explanations for these discrepancies include recall bias or methodological differences between studies, though it is notable that incidence rates of EDs and hospitalization rates remain high compared to pre-pandemic estimates [4,251,270,271]. Another class of findings highlighted enduring cultural contexts. Regarding risk for ED onset, one review found lower rates of EDs in non-western cultures and among migrants compared to local inhabitants. Historic debate over the culture-bound nature of EDs [272] has been countered with a focus on the biological contribution to EDs like AN. Nevertheless, research efforts might determine features specific to non-western cultures that could prove protective.

### 4.2. Recommended Research Directions

Turning to the future, our review highlighted areas of needed focus in ED research. Research funding has traditionally not matched the clinical priority that EDs present (accordingly to recent data, $0.73 is spent per affected individual with an ED versus $86.79 per year per affected individual with schizophrenia; [273]). Our review underscores the need to devote more resources to help understand the etiology, maintenance, and treatment of EDs. It is also notable that most of the meta-analyses included in this review were critically flawed as per the AMSTAR 2 assessment; increasing research funding for EDs will hopefully improve the quality of studies that are undertaken across all topic areas.

Future research into the underlying biological processes of EDs is crucial, particularly transdiagnostic cognitive processes, including repetitive negative thinking or rumination. Although research is currently limited, these cognitive patterns are present across ED samples [252] and thus hold high potential as treatment targets for EDs and comorbid psychopathology (e.g., depression, anxiety [274]). Multiple studies identified inefficiencies in interoception, executive function, and reward systems across EDs (e.g., [49,62,213]. In AN, cognitive inefficiencies may be secondary to malnutrition, and reward processing may be altered (rather than deficient; [62]). Whether disruption in neurocognitive processes contributes directly to cognitive and behavioral symptoms of EDs is unclear and should be prioritized in future research. A more promising approach is likely to involve direct examination of the correlates and mechanisms of eating disorder symptoms. Whether biobehavioral mechanisms are related to treatment response is also a research priority and may ultimately facilitate treatment matching.

The review also highlighted a clear need for additional research to understand the utility of neuromodulation and how it can be optimized for the treatment of EDs. How pharmaceutical approaches (e.g., naltrexone for dampening food-related reward) might best be used (e.g., at what point during treatment, and at what dose) also requires further examination. Recent escalation in the use of GLP-1 agonist medications for obesity have implications for binge eating, given the comorbidity of these conditions [275,276]. Their rapid uptake is generating increased concern within the ED field, with interest in their potential to reduce symptoms set against concern that their widespread use for weight loss may precipitate or mask restrictive EDs [277].

The different cluster sizes suggest that the limited funding for and attention towards EDs has been disproportionately directed towards certain research topics. For example, ARFID has received much more limited research attention compared to other disorders. While anorexia nervosa is much more researched than other EDs, relatively few studies have focused on pharmacological approaches to treating anorexia nervosa versus other types of treatment.

Our findings emphasize the need for improvements in research practice. Consistent surveillance would improve ED incidence and prevalence reporting. Measurement heterogeneity, particularly for ARFID, underscores the need for consensus diagnostic tools to facilitate cross-study comparisons. Despite being more common than AN, ARFID, BN, BED, and OSFED have received less research attention [7], and advances in the understanding of these disorders are predicated on this pattern in research focus changing.

Although specific clinical guidance is beyond the scope of an umbrella review, one practical implication is that brief screeners for ED symptoms (e.g., Brief Assessment of Stress and Eating (BASE-10 [278]) could be more widely used across settings (e.g., university; primary care) with priority given to tools that show measurement invariance across sex, race/ethnicity, and weight status. Likewise, interventions for underrepresented groups should be adapted using established cultural adaptation frameworks (e.g., the Ecological Validity Model [279]) and co-designed with people with lived experience from those communities (e.g., PEACE pathway for autistic individuals with EDs [280]); trials should, in turn, report outcomes by demographic subgroup so that the need for such adaptation can be tested empirically.

### 4.3. Use of AI in the Process of Conducting an Umbrella Review

Beyond content findings, our review offers a roadmap for using AI in research. AI automated repetitive tasks (data extraction, quality assessment), accelerating these activities considerably. Neural topic modeling [43] organized the literature, which sped up the synthesis process and reduced reliance on predefined human-generated categories (though it should be acknowledged that other biases may have been introduced, and that methodological subjectivity is not mitigated). Notably this sometimes led to categorization of articles by characteristics that may be less obvious to human reviewers. For example, ED treatments or studies were sometimes grouped by proposed underlying mechanism (or for treatments, mechanism of action) rather than treatment modality.

This AI-assisted review revealed several key considerations for ensuring rigor in similar future work. First, AI tools should be selected based on alignment with research goals. We chose Claude as the LLM for its emphasis on accuracy, as noted across several scientific applications [281,282,283]. As LLMs evolve, piloting various models for task suitability will be important. Second, validation thresholds should be established before proceeding; while we used existing best practices, future work should determine optimal human oversight levels and accuracy standards. Finally, some experimentation and iteration may be necessary. We conducted preliminary analyses to optimize information extraction, refine prompts, and evaluate neural topic modeling. While this precluded pre-registration, it enabled more effective use of AI tools.

Certain stages of this review could not be automated. Reviewing articles alongside supplemental and protocol files enabled a fairer quality assessment, but manual uploading was burdensome, highlighting the value of unified systems for linked study materials. Importantly, Claude could not write our discussion; even with advanced tools, human experience remains essential for contextualizing and interpreting research findings appropriately. It is notable that while few in number, there were instances in which sentences describing the collection of articles in a given category appeared incoherent, raising interesting questions about the mechanisms by which LLMs were producing article summaries (and highlighting room for improvement).

### 4.4. Strengths and Limitations

Strengths of this study include our transparency, iterative approach, adherence to best practices, and commitment to rigor (requiring team consensus, ensuring replicability, making all code available). Limitations include lack of preregistration and acknowledgment that methodological choices different from ours may have led to different results. We excluded animal research, non-official ED presentations (e.g., food addiction, orthorexia), and studies of sub-clinical eating pathology (where an ED outcome was not present). Topic models used abstracts rather than full texts; while consistent with recent work [28,29,284] full-text processing may have produced different groupings of research topics. This possibility should be explored in future research, and the identified themes should be interpreted as reflecting the semantic content in abstracts rather than what might be present in recent eating disorders research. While the topic model was selected for its best performance across several criteria, different topic model configurations (perhaps performing best per different criteria or metrics) may produce different thematic structures. LLMs can hallucinate [285], respond in a prompt and training data-dependent way, and are subject to specific biases (e.g., focusing most on words earlier or later in a document [286]) that are yet to be fully elucidated and understood. While our assessments of LLM accuracy for data extraction, quality appraisal and Phase One category summaries indicated good performance for these tasks, assessment was limited to a subset of articles and clusters, respectively. For more confident conclusions in LLM accuracy clearly the full set of articles and clusters would need to be examined, and it should be acknowledged that there is some randomness in the LLMs that prevents reproducibility. In considering review consistency it should be considered that there may have been overlap in the studies included in the separate meta-analyses, which can contribute to apparent similarity between conclusions of distinct review articles. We did not assess this because our inclusion criteria were broad and our study is extensive in its scope. For a more constrained umbrella review, assessment of the overlap in the primary studies included in each meta-analysis would be helpful in contextualizing the evidence consistency. Finally, the results of the AMSTAR 2 quality appraisal suggest that most studies were low in quality, and consequently the degree of confidence that may be placed in the topic cluster summaries is limited. This precludes using the findings from this review to guide clinical practice in the absence of additional evidence. Nonetheless, the findings do provide a foundation for further research and are valuable in identifying priority areas. It should also be considered that AMSTAR 2 does not offer insight into the certainty of evidence for each outcome included in this review. A meta-analysis rated low or critically low on AMSTAR 2 may still report a robust effect, and a high-quality review may report imprecise or inconsistent estimates. We refer readers to the Supplementary Tables for effect sizes and confidence intervals for outcomes of each included study.

## 5. Conclusions

In summary, our umbrella review of the most recent literature in the EDs field confirms prevalence and comorbidity rates, treatment outcomes, and a primary focus on AN and white, Western samples. In addition to promoting diversity in our samples, suggestions for future translational work include efforts to link neurocognitive and neuroimaging data directly to behavioral symptoms and outcomes, use of longitudinal designs, and further examination of novel neuromodulation therapies. The use of AI tools accelerated the review process, supporting their potential utility in assisting human-led scientific research. Future studies should directly compare human effort with AI tools for performing scientific research tasks to more rigorously identify the strengths and limitations of assistance from AI tools, both with and without human supervision.

## Figures and Tables

**Table 1:** Inclusion/exclusion criteria for the review.

**Population**	Humans with a diagnosed eating disorder or clinically significant ED psychopathology (if diagnosed eating disorder was specified as the outcome); food addiction or orthorexia nervosa only when examined in people with EDs	Animal studies; sub-clinical disordered eating; overweight/obesity populations (including bariatric surgery) without an ED-related outcome; mixed diagnostic samples (e.g., ED plus psychosis) without separate ED results
**Outcomes**	Any ED-related outcome (e.g., treatment efficacy, symptom change, comorbidity, prevalence, mortality, neurocognitive or biomarker differences)	Outcomes not reported separately for ED groups
**Study design**	Meta-analyses published 2020–2025	Primary studies; narrative or systematic reviews without meta-analysis; genome-wide association studies; any study without a systematic search

**Table 2:** Adopted BERTopic workflow.

Step	Technique
**1**	Employ a sentence transformer model to generate document embeddings that capture the semantic meaning of text. No preprocessing is required (i.e., punctuation remains in the abstracts, which aids the ability of the transformer to infer context from the text input).
**2**	Reduce the dimension of the extracted embeddings using Uniform Manifold Approximation and Projection (UMAP ([44])). This method retains the local structure as well as the global structure of high-dimensional data when reducing it to fewer dimensions.
**3**	Cluster the embeddings in the reduced dimensional space. K-means clustering was used to determine topic categories; alternative methods (e.g., Hierarchical Density-Based Spatial Clustering of Applications with Noise; HBDscan) yielded a significant number of outlier articles (~one-third).

**Table 3:** Details of identified topics.

Topic Number	Label	Article Count	AMSTAR 2 Rating Distribution 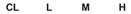	Reference List
1	Neurocognition	26	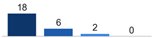	[46,47,48,49,50,51,52,53,54,55,56,57,58,59,60,61,62,63,64,65,66,67,68,69,70,71]
2	Eating Disorder Treatment Effectiveness	24	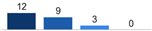	[72,73,74,75,76,77,78,79,80,81,82,83,84,85,86,87,88,89,90,91,92,93,94,95]
3	Eating Disorder Treatment Intervention Efficacy	23	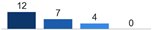	[96,97,98,99,100,101,102,103,104,105,106,107,108,109,110,111,112,113,114,115,116,117,118]
4	Biomarkers in Eating Disorders	19	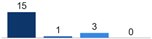	[119,120,121,122,123,124,125,126,127,128,129,130,131,132,133,134,135,136,137]
5	Eating Disorders and Premorbid and Comorbid Conditions	18	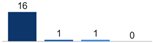	[138,139,140,141,142,143,144,145,146,147,148,149,150,151,152,153,154,155]
6	Eating Disorders Clinical Characteristics	17	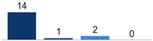	[156,157,158,159,160,161,162,163,164,165,166,167,168,169,170,171,172]
7	Global Prevalence and Mortality in Eating Disorders	14	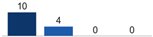	[2,173,174,175,176,177,178,179,180,181,182,183,184,185]
8	Mental Health Comorbidity and Prevalence	13	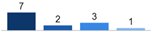	[186,187,188,189,190,191,192,193,194,195,196,197,198]
9	Comorbidity and Behavioral Addictions	11	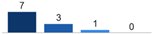	[199,200,201,202,203,204,205,206,207,208,209]
10	Treatment Outcomes: Longer-Term	10	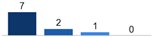	[210,211,212,213,214,215,216,217,218,219]
11	Eating Disorders Prevention Interventions	10	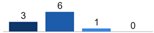	[220,221,222,223,224,225,226,227,228,229]
12	Psychological Factors in Eating Disorders	9	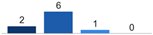	[230,231,232,233,234,235,236,237,238]
13	Eating Disorders Treatment Gaps and Outcomes	9	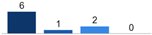	[239,240,241,242,243,244,245,246,247]
14	COVID-19 Pandemic Mental Health Impact Studies	8	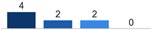	[248,249,250,251,252,253,254,255]
15	Pharmacological Interventions for Anorexia Nervosa	6	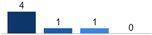	[10,11,256,257,258,259]
16	ARFID Clinical Characteristics and Prevalence	3	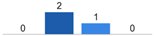	[260,261,262]
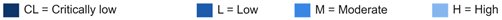 Bar heights show the proportion of articles in each topic receiving each AMSTAR 2 rating (same 0–100% scale in every row); numbers above bars are article counts.				

## Data Availability

Data from the systematic reviews summarized are available in the original publications. Data extracted from these articles is presented in the Supplementary Material, along with outcomes of the Quality Assessment and LLM summaries of each article category (derived from the topic modeling).

## Supplementary Materials

The following supporting information can be downloaded at: https://www.mdpi.com/article/10.3390/nu18213650/s1: Supporting Material (containing: Systematic Search Terms; Generative AI Prompts; Data extraction; Prompt to summarize articles in each category (Phase 1 summary); Table S1: Summary of Decisions made during the Review:; Table S2: Topic Modeling Settings; Table S3: Summary of Prompt Changes Over Time; Table S4: Topic Model Comparison; Table S5: Corrections of Phase Two Summaries; Table S6: Quality Assessment (AMSTAR 2) of all included Studies); Table S7: AMSTAR 2 data validation; Full Phase One and Phase Two Summaries; Supplementary Tables.
