# Supplementary material for: An Artificial Intelligence-Assisted Umbrella Review of Eating Disorder Meta-Analyses (2020–2025): Mapping Treatment Efficacy, Comorbidity, and Persistent Gaps in Sample Diversity
Source: Nutrients. 2026 Nov 3;18(21):3650. doi: 10.3390/nu18213650 (PMC13765187; doi:10.3390/nu18213650)
Supplement: Supplementary file 1 [file nutrients-18-03650-s001.zip › nutrients-4563069-supplementary-material.pdf]

Supporting Material for **An Artificial Intelligence-Assisted Umbrella Review of Eating Disorder Meta-Analyses (2020–2025): Mapping Treatment Efficacy, Comorbidity, and Persistent Gaps in Sample Diversity**

Table of Contents

***Systematic Search Terms* 2**

***Generative AI Prompts*.. 5**

  Data extraction 5

  Prompt to summarize articles in each category (Phase 1 summary)........................ 8

***Table S1: Summary of Decisions made during the Review:* 11**

***Table S2: Topic Modeling Settings*... 11**

***Table S3: Summary of Prompt Changes Over Time* 12**

***Table S4: Topic Model Comparison*... 15**

***Table S5: Corrections of Phase Two Summaries*.. 16**

***Table S6: Quality Assessment (AMSTAR 2) of all included Studies* 17**

***Table S7: AMSTAR 2 data validation* 24**

Systematic Search Terms

PubMed

June 9th 2025

meta-analysis[Title/Abstract] AND (anorexia nervosa[Title/Abstract] OR bulimia nervosa[Title/Abstract] OR binge eating disorder[Title/Abstract] OR avoidant/restrictive food intake disorder[Title/Abstract] OR avoidant restrictive food intake disorder[Title/Abstract] OR atypical anorexia nervosa[Title/Abstract] OR other specified feeding or eating disorder[Title/Abstract] OR eating disorder not otherwise specified[Title/Abstract] OR purging disorder OR night eating syndrome[Title/Abstract] OR eating disorder[Title/Abstract])

Filters: from 2020/6/1–2025/5/31

July 3rd 2025

meta-analysis[Title/Abstract] and (pica[Title/Abstract] OR rumination disorder[Title/Abstract])

Medical Subject Headings (MeSH terms):

("Meta-Analysis as Topic"[MeSH Terms]) AND ("Anorexia Nervosa"[MeSH Terms] OR "Bulimia Nervosa"[MeSH Terms] OR "Binge-Eating Disorder"[MeSH Terms] OR "Avoidant Restrictive Food Intake Disorder"[MeSH Terms] OR "Feeding and Eating Disorders of Childhood"[MeSH Terms] OR "Night Eating Syndrome"[MeSH Terms] OR "Pica"[MeSH Terms] OR "Rumination Syndrome"[MeSH Terms])

July 3rd 2025

("Meta-Analysis as Topic"[MeSH Terms]) AND ("Pica"[MeSH Terms] OR "Rumination Syndrome"[MeSH Terms])

PsycINFO

June 9th 2025

(title: meta-analysis OR abstract: meta-analysis) AND (title: anorexia nervosa OR abstract: anorexia nervosa OR title: bulimia nervosa OR abstract: bulimia nervosa OR title: binge eating disorder OR abstract: binge eating disorder OR title: avoidant/restrictive food intake disorder OR abstract: avoidant/restrictive food intake disorder OR title: avoidant restrictive food intake disorder OR abstract: avoidant restrictive food intake disorder OR title: atypical anorexia nervosa OR abstract: atypical anorexia nervosa OR title: "other specified feeding or eating disorder" OR abstract: "other specified eating disorder" OR title: purging disorder OR abstract: purging disorder OR title: night eating syndrome OR abstract: night eating syndrome OR title: "eating disorder not otherwise specified" OR abstract: "eating disorder not otherwise specified" OR title: eating disorder OR abstract: eating disorder)

July 3rd 2025

(title: meta-analysis OR abstract: meta-analysis) AND (title: rumination disorder OR abstract: rumination disorder)

Web of Science

June 9th 2025

(TI=("meta-analysis") OR AB=("meta-analysis")) AND (TI=("anorexia nervosa") OR AB=("anorexia nervosa") OR TI=("bulimia nervosa") OR AB=("bulimia nervosa") OR TI=("binge eating disorder") OR AB=("binge eating disorder") OR TI=("avoidant/restrictive food intake disorder") OR AB=("avoidant/restrictive food intake disorder") OR TI=("avoidant restrictive food intake disorder") OR AB=("avoidant restrictive food intake disorder") OR TI=("atypical anorexia nervosa") OR AB=("atypical anorexia nervosa") OR TI=("other specified feeding or eating disorder") OR AB=("other specified feeding or eating disorder") OR TI=("other specified eating disorder") OR AB=("other specified eating disorder") OR TI=("eating disorder not otherwise specified") OR AB=("eating disorder not otherwise specified") OR TI=("purging disorder") OR AB=("purging disorder") OR TI=("night eating syndrome") OR AB=("night eating syndrome") OR TI=("eating disorder") OR AB=("eating disorder"))

July 3rd 2025

(TI=("meta-analysis") OR AB=("meta-analysis")) AND (TI=("pica") OR AB=("pica") OR TI=("rumination disorder") OR AB=("rumination disorder"))

Embase

June 9th 2025

(meta-analysis:ti,ab) AND ('anorexia nervosa':ti,ab OR 'bulimia nervosa':ti,ab OR 'binge eating disorder':ti,ab OR 'avoidant/restrictive food intake disorder':ti,ab OR 'avoidant restrictive food intake disorder':ti,ab OR 'atypical anorexia nervosa':ti,ab OR 'other specified feeding or eating disorder':ti,ab OR 'other specified eating disorder':ti,ab OR 'eating disorder not otherwise specified':ti,ab OR 'purging disorder':ti,ab OR 'night eating syndrome':ti,ab OR 'eating disorder':ti,ab)

July 3rd 2025

(meta-analysis:ti,ab) AND (pica':ti,ab OR 'rumination disorder':ti,ab OR 'eating disorder':ti,ab)

ProQuest

June 23rd 2025

(TI(meta-analysis) OR AB(meta-analysis)) AND (TI("anorexia nervosa") OR AB("anorexia nervosa") OR TI("bulimia nervosa") OR AB("bulimia nervosa") OR TI("binge eating disorder") OR AB("binge eating disorder") OR TI("avoidant/restrictive food intake disorder") OR AB("avoidant/restrictive food intake disorder") OR TI("avoidant restrictive food intake disorder") OR AB("avoidant restrictive food intake disorder") OR TI("atypical anorexia nervosa") OR AB("atypical anorexia nervosa") OR TI("other specified feeding or eating disorder") OR AB("other specified feeding or eating disorder") OR TI("other specified eating disorder") OR AB("other specified eating disorder") OR TI("purging disorder") OR AB("purging disorder") OR TI("night eating syndrome") OR AB("night eating syndrome") OR TI("eating disorder not otherwise specified") OR AB("eating disorder not otherwise specified") OR TI("pica") OR AB("pica") OR TI("rumination disorder") OR AB("rumination disorder") OR TI("eating disorder") OR AB("eating disorder"))

July 3rd 2026

(TI(meta-analysis) OR AB(meta-analysis)) AND ("pica") OR TI("rumination disorder") OR AB("rumination disorder"))

Generative AI Prompts

Data extraction

For each study, the following prompt was fed to the Claude API, using a separate chat for each call.

"*You are conducting an AMSTAR 2 quality assessment of a systematic review/meta-analysis.*

IMPORTANT: Check ALL provided documents (main article, supplements, and protocol) for information.

AMSTAR 2 has 16 items. For each item below, provide a response in this EXACT JSON format:

[

{{

"Section": "AMSTAR_Items",

"Field": "Item_1",

"Value": "Yes/No/Partial Yes. [Provide a detailed explanation with evidence from the paper/supplement/protocol]"

}}

]

Note that not all items can have a "Partial" response; this is limited to questions: 2,4,7,8,9. For information about how to differentiate "Partial Yes" from "Yes" see the SPECIAL ATTENTION section below.

AMSTAR 2 ITEMS TO ASSESS:

1. Did the research questions and inclusion criteria include components of PICO? Did this include: Population, Intervention, Comparator and Outcome?

2. Did the report contain an explicit statement that methods were established prior to conduct? Did the report justify any significant deviations from the protocol? (CHECK SUPPLEMENTS/PROTOCOL)

3. Did the authors explain their selection of study designs for inclusion? This should include reasons for including randomized controlled trials or non-randomized studies or both

4. Did the authors use a comprehensive literature search strategy? (CHECK SUPPLEMENTS)

5. Did the authors perform study selection in duplicate?

6. Did the authors perform data extraction in duplicate? This should involve either 1) at least two reviewers achieved consensus on which data to extract from included studies; or 2) two reviewers extracted data from a sample of eligible studies and achieved good agreement (at least 80 percent), with the remainder extracted by one reviewer.

7. Did the authors provide a list of excluded studies and justify exclusions? (CHECK SUPPLEMENTS)

8. Did the authors describe included studies in adequate detail? This includes a description of populations, interventions, comparator interventions, outcomes, and research designs.

9. Did the authors use satisfactory technique for assessing risk of bias? (CHECK SUPPLEMENTS)

10. Did the authors report on sources of funding for included studies? Must have reported on the sources of funding for individual studies included in the review.

11. If meta-analysis performed, did authors use appropriate statistical methods? This involves:

justifying combining the data in a meta-analysis; using an appropriate weighted technique to combine study results and adjusted for heterogeneity if present; and investigating causes of heterogeneity (randomized controlled study only).

For a non-randomized study authors must justify combining raw estimates or only combine estimates adjusted for confounding, and report separate estimates from randomized controlled studies and non-randomized studies if applicable.

12. If meta-analysis performed, did authors assess impact of risk of bias? This should include sub-analyses that examine whether findings are different when only low risk of bias studies are included, or examine the moderating effect of risk of bias on findings.

13. Did the authors account for risk of bias when interpreting results?

14. Did the authors provide satisfactory explanation for heterogeneity observed? If heterogeneity was present the authors performed an investigation of sources of any heterogeneity in the results and discussed the impact of this on the results of the review

15. If quantitative synthesis performed, did authors investigate publication bias? Performed graphical or statistical tests for publication bias and discussed the likelihood and magnitude of impact of publication bias

16. Did the authors report potential sources of conflict of interest? The authors reported no competing interests OR The authors described their funding sources and how they managed potential conflicts of interest

ASSESSMENT CRITERIA:

- "Yes" = Criterion clearly met

- "No" = Criterion clearly not met

- "Partial Yes" = Criterion partially met or with limitations

- "Not applicable" = Does not apply to this study type

- Note that only items 2,4,7,8,9 can have the response "Partial Yes"; for other items only "Yes" or "No" is possible

SPECIAL ATTENTION:

- Items 2, 4, 7, 9 often have details in supplements or protocols

- For Item 2, for the rating to be "Partial Yes" the authors must have stated that they had a written protocol or guide that included ALL the following: review question(s); a search strategy; inclusion/exclusion criteria; a risk of bias assessment

- For Item 2, for the rating to be "Yes" criteria for partial yes should be fulfilled and the protocol should be registered and should also have specified: a meta-analysis/synthesis plan; plan for investigating causes of heterogeneity

- For Item 4, for the rating to be "Partial Yes" the authors must have searched at least 2 databases (relevant to research question); provided key word and/or search strategy; and justified publication restrictions (e.g. language)

- For Item 4, for the rating to be "Yes" criteria for partial yes should be fulfilled and the authors should have: searched the reference lists / bibliographies of included studies; searched trial/study registries; included/consulted content experts in the field; where relevant, searched for grey literature; conducted search within 24 months of completion of the review

- For Item 7; for the rating to be "Partial Yes" authors must have provided a list of all potentially relevant studies that were read in full-text form but excluded from the review

- For Item 7; for the rating to be "Yes" criteria for partial should be fulfilled, and the authors must have justified the exclusion from the review of each potentially relevant study

- For Item 8; to move from a "Partial Yes" to a "Yes" the article should give a detailed description of population, intervention, comparator and describe each study's setting and the timeframe for follow-up

- For Item 9; for the rating to be "Partial Yes", the following should be satisfied:

FOR RCT: risk of bias from unconcealed allocation, and lack of blinding of patients and assessors when assessing outcomes (unnecessary for objective outcomes such as all-cause mortality) should have been assessed

FOR non-randomized study: risk of bias from confounding and selection bias should have been assessed

For the rating to be "Yes", in addition to the criteria for "Partial Yes", the article should have assessed risk of bias from:

RCT: allocation sequence that was not truly random, and selection of the reported result from among multiple measurements or analyses of a specified outcome

non-randomized study: methods used to ascertain exposures and outcomes, and selection of the reported result from among multiple measurements or analyses of a specified outcome

- Search supplement/protocol text carefully for registration info, search strategies, excluded study lists, and bias assessment details

- Cite which document (main/supplement/protocol) contains the evidence

Data extraction

For each question in the data extraction CSV sheet:

Extract specific data from this research study and format as JSON array.

Each object must have exactly these keys: "Section", "Field", and "Value".

For each field above, provide:

- "Section": The appropriate section name

- "Field": The EXACT field name from the list above

- "Value": The extracted data from the article

EXTRACTION GUIDELINES:

- For country of article, report the country in which the corresponding author is based

- Report what the findings of the statistical analyses were for primary and second/third outcomes, the "Focus and main finding for outcome" field.

- For statistical results, provide EXACT numbers (OR, CI, p-values). You need to identify the primary outcome and the second and third outcomes of focus. For each one, provide the effect size along with the confidence interval and p-value. Be as comprehensive as you can.

- You do not need to report the statistical results of any meta-regression; only all meta-analytic findings.

- Provide information about the eating disorder or disorders of focus

- Describe the included population of the study, and specifically their sex, gender, race, ethnicity, age and socioeconomic status

- Report the number of studies in each separate meta-analysis

- For sample sizes, provide exact participant counts

- For missing data, write "Not reported" or "Not available"

- For yes/no questions, write "Yes", "No", or "Unclear"

"

Prompt to summarize articles in each category (Phase 1 summary)

"*There are X total articles from a systematic umbrella review, which formed a coherent cluster. Please synthesize these into a final analysis.*

Please provide:

*1. **Overall Cluster Label**: Descriptive label for all articles (max 6 words)*

Indicate whether there are subcategories of studies within this collection of articles.

*2. **Overall Findings Summary**:*

What are the main conclusions and themes across all studies?

Please provide effect sizes and confidence intervals for the main analyses, and indicate the findings of subgroup or moderation analyses (again using effect size and confidence interval statistics).

Please provide information about the study or studies being referred to for each point.

You should describe the most scientifically relevant as well as the most clinically relevant findings.

3. **Population Analysis**:

For each demographic category, specify which articles (by number/name) include this information:

- Gender distribution and representation (specify which articles report gender data)

- Socioeconomic status of participants (specify which articles include SES data)

- Race and ethnicity breakdown (specify which articles report race/ethnicity data)

- Any notable demographic gaps or biases across the studies

4. **Study Comparison**:

- Similarities in study designs, methodologies, and approaches (specify which articles share these features)

- Differences in populations, settings, and methods (specify which articles differ and how)

- Convergent vs. divergent findings across the collection of studies (specify which articles support or contradict each finding)

- Describe differences in the conclusions of the studies, and similarities in the conclusions.

5. **Article-Specific Information**:

For each article, provide a brief summary including:

- Main research question/objective

- Study design and methodology

- Key population characteristics

- Primary findings (give effect size and confidence intervals)

- Outcomes of subgroup or moderator analyses (give effect size and confidence intervals)

- Notable limitations

“

Prompt to produce a short summary of articles within a given category (Phase 2 summary)

"*You are reviewing a text summary of a collection of scientific articles. The articles have been obtained from a systematic search and were grouped into this category based on their similarity. Article similarity was determined using a neural topic model.*

IMPORTANT: You need to read the summary carefully and extract the key points across all of the articles. You can include key statistics if they seem particularly relevant - in this case please cite the paper from which you have taken the statistics (i.e., give the details of the author and the year). If there are any novel findings, or new ideas for future research identified in the summaries, please describe them and indicate the article that the conclusion has been drawn from by citing the author.

In total the summary should be no more than two paragraphs. The output will be included in a scientific journal article that will be read by mental health researchers and practitioners."

Table S1: Summary of Decisions made during the Review:

Date	Area	Decision	Status
**06/23**	Inclusion	Umbrella reviews and network meta-analyses are excluded	Current
**06/23**	Inclusion	Orthorexia included; food addiction excluded	Superseded 07/02
**06/23**	Inclusion	Studies of broader populations (e.g., psychiatric diagnoses) are included if they report a separate analysis of the eating disorder group	Current (reaffirmed 07/02, 07/16)
**06/23**	Inclusion	Any study with some form of meta-analysis and a systematic search is included for now	Current
**06/23**	Inclusion	Population must be diagnosed or meet a clinical threshold of psychopathology, or diagnosis/clinically significant pathology must be an outcome	Current
**06/23**	Screening	Everyone resolves conflicts at title/abstract screening; a dedicated role will be assigned for full-text screening	Current
**07/02**	Inclusion	Studies with multiple psychiatric populations are included	Current
**07/02**	Inclusion	Prevention studies are included if meeting a clinical threshold or diagnosis is an outcome	Current
**07/02**	Inclusion	Studies of ED prevalence are included	Current
**07/02**	Inclusion	Orthorexia excluded (not in search terms, not a DSM-5 ED), unless studied within an ED population	Current (clarified 07/16)
**07/02**	Search	Pica and rumination disorder added to search terms; searches to be run to include these terms	Current
**07/07**	Screening	Use tags to record exclusion reasons and each study’s broad topic; create new tags as needed	Current
**07/16**	Inclusion	Orthorexia-only studies excluded; included if a diagnosed ED group is studied for orthorexia symptoms or prevalence	Current
**07/16**	Inclusion	Studies with a subgroup analysis of an ED group are included (identifiable from the methods section)	Current
**07/16**	Inclusion	Authors’ reports of a diagnosed ED sample are accepted, but ED status inferred from a non-validated tool is excluded	Current
**07/16**	Extraction	Explore automated data extraction (GPT-4 and Claude)	Current
**07/16**	Extraction	Validate automated extraction against manual extraction of 10% of studies	Current

Table S2: Topic Modeling Settings

Step	Setting
**Software**	Python; BERTopic 0.17.3, sentence-transformers 5.1.0, umap-learn 0.5.9, scikit-learn 1.7.1
**Input**	Abstracts of the 220 included reviews
**Preprocessing**	Abstracts were embedded as written, with no stop-word removal, stemming or lemmatization before embedding

**Embedding model**	pritamdeka/S-PubMedBert-MS-MARCO, a biomedical sentence-transformer. It was compared with all-MiniLM-L6-v2
**Dimensionality reduction**	UMAP: n_neighbors = 15, n_components = 4, min_dist = 0.0, metric = cosine, random_state = 42
**Clustering**	k-means; k = 7, 10, 13 and 16 tested; final k = 16
**Topic keywords**	CountVectorizer (English stop words removed, min_df = 2, unigrams and bigrams); class-based TF-IDF; KeyBERTInspired representation; top 20 words per topic
**Model selection**	UMass coherence (top 12 words per topic) and topic diversity (share of unique words among each topic's top 10), across both embedding models and all values of k
**Outliers**	k-means assigns every abstract to a cluster, so there is no outlier topic and no abstracts were excluded. HDBSCAN, which creates an outlier category, was not used
**Topic labels**	Assigned by the authors from each topic's keywords, representative abstracts and LLM-generated summaries
**Visualization**	Separate 2D UMAP (n_neighbors = 10, min_dist = 0.0, cosine), used only for the figure. Stop words were removed for the word clouds only
**Final solution**	16 topics, containing 3 to 26 abstracts each

Table S3: Summary of Prompt Changes Over Time

Prompt	Version and details
**Data Extraction Prompt for AMSTAR2 assessment**	**v1.0**<br><br>Rates the 16 AMSTAR 2 items, each in one line, as Yes / No / Partial / Not applicable, with evidence.<br><br>**v2.0**<br><br>• Allows "Partial Yes" only for items 2, 4, 7, 8 and 9.<br>• Asks for fuller explanations.<br><br>**v3.0**<br><br>• Reads the supplements and protocol as well as the main article.<br>• Cites which document each piece of evidence came from.<br>• Gives each item the full AMSTAR 2 guidance wording.<br>• Adds exact Partial Yes vs. Yes criteria for items 2, 4, 7, 8 and 9.
**Data Extraction Prompt for Study Data**	**v1.0**<br><br>Uses a generic checklist: study ID, objectives, sample, methods, results, quality and clinical significance.<br><br>Asks for exact numbers.

<table>
<tr><td></td><td>Adds the QC-sheet field names at the end.

v2.0

Replaces the generic checklist with the QC-sheet fields as a required list.

Adds short rules:
exact OR, CI and p-values
describe the population
number of studies in each meta-analysis
"Not reported" for missing data
Yes / No / Unclear for yes/no questions
v3.0

Asks for the primary, second and third outcomes, each with effect size, CI and p-value.

Uses the corresponding author's country.

Names the eating disorder(s) of focus.

Widens the population fields to sex, gender, race, ethnicity, age and SES.</td></tr>
<tr><td>Phase One Summary prompt</td><td>v1.0

Covers four sections: overall findings, population, study comparison and synthesis. All are pooled across the cluster.

v2.0

Insists all articles be analyzed.

Adds a cluster label (max 6 words), subcategories, per-article summaries and per-article demographics.

v3.0

Asks for effect sizes and CIs for main, subgroup and moderator results.

Asks that source article is named for every claim.

Adds a fixed per-article template: question, design, population, findings and limitations.

v4.0</td></tr>
</table>

	Asks for the most clinically relevant findings and author names.<br><br>Splits large clusters into batches, each summarized with its own prompt, then combines the results with a final merging prompt.
**Phase Two Summary Prompt**	**v1.0**<br><br>Asks for the key points across all the articles.<br><br>Allows key statistics, but each one must be cited by author and year.<br><br>Asks for novel findings and future research ideas, each cited to its source article.<br><br>Sets the length at two paragraphs at most**.**<br><br>Names the audience as mental health researchers and practitioners.<br><br>**v2.0**<br><br>Asks for the most consistent findings rather than all key points.<br><br>Focuses on conclusions that matter for future research and clinical practice.<br><br>Drops the separate request for novel findings.<br><br>Simplifies the citation rule to "cite the relevant article".<br><br>Cuts the length to one short paragraph.

1.

Table S4: Topic Model Comparison

Sentence Transformer	K number of clusters	Topic Coherence	Topic Diversity
pritamdeka/S-PubMedBert-MS-MARCO	7	-4.241599867	0.5142857143
pritamdeka/S-PubMedBert-MS-MARCO	10	-4.907678246	0.55
pritamdeka/S-PubMedBert-MS-MARCO	13	-5.087779404	0.6153846154
pritamdeka/S-PubMedBert-MS-MARCO	16	-5.437630026	0.61875
all-MiniLM-L6-v2	7	-4.732979681	0.5714285714
all-MiniLM-L6-v2	10	-4.664575758	0.5
all-MiniLM-L6-v2	13	-5.062274729	0.5846153846
all-MiniLM-L6-v2	16	-4.64931547	0.54375

Table S5: Corrections of Phase Two Summaries

Topic	Type	Phase 2 summary	Manuscript
**All**	Content removed	Closing sentence on demographic limitations and/or clinical implications	Removed
**2, 10**	Clarification added	"Abstinence rates"	"Abstinence (from binge eating) rates"
**2, 5, 6, 8, 9, 14, 16**	Count added	No article count, or described as "systematic reviews"/"meta-analyses"	"This cluster of [n] articles"
**6***	Number mismatch	Sleep time reduced by 21 min; sleep efficiency down 3.3%	Sleep time reduced by 32.1 min; no sleep efficiency
**6***	Number mismatch	ASD traits: effect sizes 0.77–0.79; 18% above cutoff	g = 0.88; 29% above cutoff
**7**	Content moved	Impulse control disorders (22%) listed with binge-purge comorbidities	Moved into main comorbidity list
**8**	Content removed	Preterm birth → ADHD (5×) and autism (10×) risk (Anderson 2021); autism comorbidities (Micai 2023)	Removed
**13**	Content removed	SCOFF screening: 84% sensitivity, 80% specificity (Feltner 2022)	Removed
**14**	Statistic label	MD = 0.44, no CI	Standardized mean difference = 0.44 (95% CI −1.30, 2.18)
**15***	Meaning changed	Oxytocin dysregulation in "restricting and binge-eating/purging subtypes"; oxytocin helps non-psychiatric populations	"Both subtypes"; non-psychiatric contrast removed
**16*+**	Number mismatch	Heart rate WMD = 12.82 bpm (95% CI 7.58–18.12)	WMD = 12.93 bpm (95% CI 8.65–17.21)
**16**	Content removed	Quality-effects method; 15-fold higher than prior estimates; male or equal sex ratio	Removed
***Considered an error; + counted as 3 errors**			

Table S6: Quality Assessment (AMSTAR 2) of all included Studies

Study	Cluster	AMSTAR 2 RATING (ITEMS 1-16)	
Breuer2024 [46]	1		CRITICALLY LOW
Bronleigh2021 [47]	1		CRITICALLY LOW
Christensen2023 [48]	1		LOW
Cobbaert2024 [49]	1		LOW
Colton2023 [50]	1		LOW
Cury2020 [51]	1		CRITICALLY LOW
Datta2025 [52]	1		CRITICALLY LOW
Dolan2022 [53]	1		CRITICALLY LOW
Fortea2025 [54]	1		LOW
Gao2025 [55]	1		CRITICALLY LOW
Iceta2021 [56]	1		MODERATE
Lang2021 [57]	1		LOW
Li2024 [58]	1		CRITICALLY LOW
Norrlin2025 [59]	1		CRITICALLY LOW
Preti2022 [60]	1		MODERATE
Prunell-Castane2021 [61]	1		CRITICALLY LOW
Rye2025 [62]	1		CRITICALLY LOW
Sader2022 [63]	1		CRITICALLY LOW
Simonsen2020 [64]	1		CRITICALLY LOW
Stedal2021 [65]	1		LOW
Su2021 [66]	1		CRITICALLY LOW
Walton2022 [67]	1		CRITICALLY LOW
Wan2025 [68]	1		CRITICALLY LOW
Watford2020 [69]	1		CRITICALLY LOW
Yu2024 [70]	1		CRITICALLY LOW
Zhong2024 [71]	1		CRITICALLY LOW
Alserihi2024 [72]	2		CRITICALLY LOW
Austin2025 [73]	2		LOW

Buerger2021 [74]	2	CRITICALLY LOW
Cuijpers2024 [75]	2	MODERATE
Everhart2025 [76]	2	LOW
Fuhrmann2024 [77]	2	MODERATE
Haderlein2022 [78]	2	CRITICALLY LOW
Hagan2020 [79]	2	CRITICALLY LOW
Hamid2024 [80]	2	CRITICALLY LOW
Keegan2022 [81]	2	CRITICALLY LOW
Linardon2020 [82]	2	CRITICALLY LOW
Low2021 [83]	2	CRITICALLY LOW
Millard2023 [84]	2	LOW
Moberg2021 [85]	2	LOW
Moghimi2021 [86]	2	CRITICALLY LOW

Ost2024 [87]	2	LOW
Rozakou-Soumalia2021 [88]	2	LOW
Seidel2024 [89]	2	LOW
Thomas2024 [90]	2	LOW
Wang2024 [91]	2	CRITICALLY LOW
Wergeland2025 [92]	2	MODERATE
Zandieh2024 [93]	2	LOW
Zhong2024 [94]	2	CRITICALLY LOW
Zinser2022 [95]	2	CRITICALLY LOW
Argyrou2023 [96]	3	LOW
Bahadori2025 [97]	3	CRITICALLY LOW
Bilbao2022 [98]	3	CRITICALLY LOW
Bravo2024 [99]	3	CRITICALLY LOW
Cavicchioli2022 [100]	3	LOW
Duggan2025 [101]	3	MODERATE
Fang2025 [102]	3	LOW
Fornaro2023 [103]	3	LOW
Gan2022 [104]	3	CRITICALLY LOW

Gay2022 [105]	3	CRITICALLY LOW
Grohmann2021 [106]	3	MODERATE
Karaszewska2022 [107]	3	MODERATE
Martinez-Calderon2025 [108]	3	MODERATE
Moawad2024 [109]	3	CRITICALLY LOW
Nourredine2021 [110]	3	LOW
Pennesi2024 [111]	3	CRITICALLY LOW
QuilesMarcos2021 [112]	3	CRITICALLY LOW
Radkhah2025 [113]	3	CRITICALLY LOW
Samara2024 [114]	3	CRITICALLY LOW
Schneider2021 [115]	3	LOW
Sioziou2024 [116]	3	LOW
Xu2022 [117]	3	CRITICALLY LOW
Yu2023 [118]	3	CRITICALLY LOW
Bailly2021 [119]	4	CRITICALLY LOW
Bevilacqua2024 [120]	4	MODERATE
Brennan2023 [121]	4	CRITICALLY LOW
Dani2024 [122]	4	CRITICALLY LOW
Gherbesi2024 [123]	4	CRITICALLY LOW
Karageorgiou2020 [124]	4	MODERATE
Keeler2022 [125]	4	CRITICALLY LOW
Li2022 [126]	4	CRITICALLY LOW
Lin2022 [127]	4	CRITICALLY LOW
Mai2022 [128]	4	CRITICALLY LOW
Salari2025 [129]	4	CRITICALLY LOW
Seidel2021 [130]	4	LOW
Shobeiri2022 [131]	4	CRITICALLY LOW
Smythe2021 [132]	4	MODERATE
Thavaraputta2023 [133]	4	CRITICALLY LOW

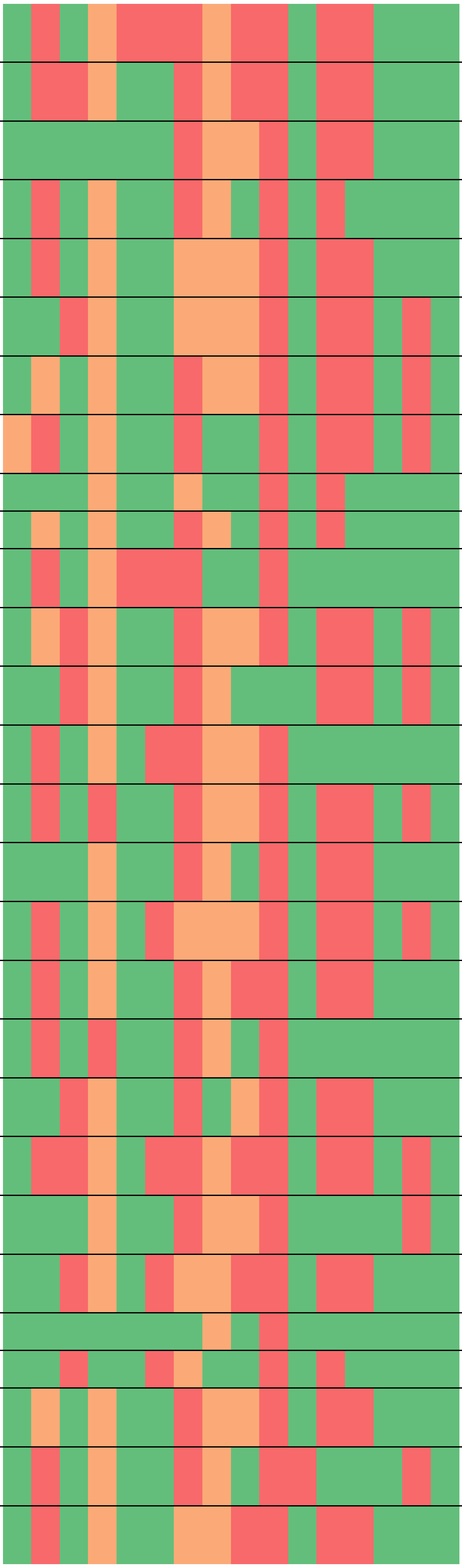

Tural2022 [134]	4	CRITICALLY LOW
Wu2024 [135]	4	CRITICALLY LOW
Xu2025 [136]	4	CRITICALLY LOW
Yan2021 [137]	4	CRITICALLY LOW
Amiri2025 [138]	5	CRITICALLY LOW
Benton2023 [139]	5	CRITICALLY LOW
Dean2024 [140]	5	CRITICALLY LOW
Fillipponi2022 [141]	5	CRITICALLY LOW
Fornaro2021 [142]	5	MODERATE
Haworth2023 [143]	5	LOW
Kjedberg2023 [144]	5	CRITICALLY LOW
Milembamane2024 [145]	5	CRITICALLY LOW
Miller2022 [146]	5	CRITICALLY LOW
Neale2020 [147]	5	CRITICALLY LOW
Nikniaz2021 [148]	5	CRITICALLY LOW
Ozturk2024 [149]	5	CRITICALLY LOW
Rasmussen2023 [150]	5	CRITICALLY LOW
Salari2025 [151]	5	CRITICALLY LOW
Sanjari2023 [152]	5	CRITICALLY LOW
Senra2024 [153]	5	CRITICALLY LOW
Trott2022 [154]	5	CRITICALLY LOW
Wilson2022 [155]	5	CRITICALLY LOW
Burger2025 [156]	6	CRITICALLY LOW
Chan2024 [157]	6	MODERATE
Dang2024 [158]	6	MODERATE
Degasperi2024 [159]	6	CRITICALLY LOW
Martinez-Gonzalez2020 [160]	6	CRITICALLY LOW
Inal-Kaleli2025 [161]	6	CRITICALLY LOW

Jenkins2024 [162]	6	CRITICALLY LOW
Johnson-Munguia2024 [163]	6	CRITICALLY LOW
Lopes2022 [164]	6	CRITICALLY LOW
Mulligan2021 [165]	6	LOW
Niederau2025 [166]	6	CRITICALLY LOW
Nijakowski2023 [167]	6	CRITICALLY LOW
Pagador2025 [168]	6	CRITICALLY LOW
Puttevils2021 [169]	6	CRITICALLY LOW
Saure2020 [170]	6	CRITICALLY LOW
Walsh2024 [171]	6	CRITICALLY LOW
Wilkop2023 [172]	6	CRITICALLY LOW
Alfalahi2022 [173]	7	CRITICALLY LOW
Amiri2023 [174]	7	CRITICALLY LOW
Amiri2024 [175]	7	CRITICALLY LOW
Devoe2022 [176]	7	CRITICALLY LOW
Drymonitou2025[177]	7	LOW
Hashemi2022 [178]	7	LOW
Kirkpatrick2024 [179]	7	CRITICALLY LOW
Krug2025 [2]	7	CRITICALLY LOW
Lo Buglio2020 [180]	7	CRITICALLY LOW
Meier2025 [181]	7	CRITICALLY LOW
Qian2022 [182]	7	CRITICALLY LOW
Sahu2025 [183]	7	LOW
Siddiqi2024 [184]	7	CRITICALLY LOW
Sohn2023 [185]	7	LOW
Anderson2021 [186]	8	CRITICALLY LOW
Chan2023 [187]	8	HIGH
Cooney2024 [188]	8	MODERATE
Drakes2021 [189]	8	CRITICALLY LOW

Faria2025 [189]	8	CRITICALLY LOW
Kwon2024 [191]	8	MODERATE
Mandelli2023 [192]	8	CRITICALLY LOW
Micai2023 [193]	8	LOW
Sacco2024 [194]	8	CRITICALLY LOW
Sharma2021 [195]	8	MODERATE
Solmi2022 [196]	8	CRITICALLY LOW
Uher2023 [197]	8	LOW
Yadav2023 [198]	8	CRITICALLY LOW
Abene2023 [199]	9	CRITICALLY LOW
Amiri2025 [200]	9	CRITICALLY LOW
Bogusz2020 [201]	9	LOW
Devoe2022 [202]	9	CRITICALLY LOW
diGiacomi2022 [203]	9	CRITICALLY LOW
Flores2025 [204]	9	CRITICALLY LOW
Jewell2023 [205]	9	MODERATE
Praxedes2022 [206]	9	LOW
Serra2022 [207]	9	CRITICALLY LOW
Taba2021 [208]	9	LOW
Trott2021 [209]	9	CRITICALLY LOW
Atti2021 [210]	10	CRITICALLY LOW
Forman2023 [211]	10	CRITICALLY LOW
Hilbert2020 [212]	10	LOW
Keegan2021 [213]	10	CRITICALLY LOW
Kops2020 [214]	10	CRITICALLY LOW
Mourilhe2021 [215]	10	CRITICALLY LOW
Radunz2020 [216]	10	CRITICALLY LOW
Raisi2023 [217]	10	LOW
Romano2020 [218]	10	CRITICALLY LOW
Tsompanaki2025 [219]	10	MODERATE
Chang2021 [220]	11	MODERATE

Davey2023 [221]	11	LOW
deBoer2023 [222]	11	LOW
Fetahi2022 [223]	11	CRITICALLY LOW
Harrer2020 [224]	11	LOW
Linardon2025_1 [225]	11	LOW
Linardon2025_2 [226]	11	LOW
Sansfacon2020 [227]	11	LOW
Stice2021 [228]	11	CRITICALLY LOW
Tuncer2025 [229]	11	CRITICALLY LOW
Alcaraz-Ibanez2020 [230]	12	LOW
Alcaraz-Ibanez2023 [231]	12	LOW
Bektas2022 [232]	12	CRITICALLY LOW
Bills2023 [233]	12	LOW
Galloway2021 [234]	12	CRITICALLY LOW
Nechita2021 [235]	12	MODERATE
Paranjothy2024 [236]	12	LOW
Stackpole2023 [237]	12	LOW
Turk2020 [238]	12	LOW
Ali2025 [239]	13	CRITICALLY LOW
Chaer2020 [240]	13	CRITICALLY LOW
Feltner2022 [241]	13	CRITICALLY LOW
Kan2021 [242]	13	CRITICALLY LOW
Kutz2020 [243]	13	MODERATE
Radunz2023 [244]	13	LOW
De Rijk2024 [245]	13	MODERATE
Sala2023 [246]	13	CRITICALLY LOW
Solmi2024 [247]	13	CRITICALLY LOW
Güzel I2023 [248]	14	MODERATE
Haghshomar2022 [249]	14	CRITICALLY LOW
Khraisat2022 [250]	14	CRITICALLY LOW
Madigan2025 [251]	14	MODERATE
Palmieri2021 [252]	14	CRITICALLY LOW
Rickerby2024 [253]	14	LOW

Sideli2021 [254]	14	LOW
vanReekum2023 [255]	14	CRITICALLY LOW
Cassioli2020 [11]	15	LOW
Chen2021 [256]	15	CRITICALLY LOW
Ferreira2022 [257]	15	MODERATE
Han2022 [10]	15	CRITICALLY LOW
Hasselbalch2020 [258]	15	CRITICALLY LOW
Kan2020 [259]	15	CRITICALLY LOW
James2024[260]	16	LOW
Nicholls-Clow2024 [261]	16	MODERATE
Sader2025 [262]	16	LOW

Table S7: AMSTAR 2 data validation

AMSTAR 2 assessment	Items assessed per reviewer	Reviewer 1, n/N (%)	Reviewer 2, n/N (%)
Overall	384	371/384 (96.6)	371/384 (96.6)
Critical domains	168	161/168 (95.8)	160/168 (95.2)
Non-critical domains	216	210/216 (97.2)	211/216 (97.7)
