# Supplementary material for: An Artificial Intelligence-Assisted Umbrella Review of Eating Disorder Meta-Analyses (2020–2025): Mapping Treatment Efficacy, Comorbidity, and Persistent Gaps in Sample Diversity
Source: Nutrients. 2026 Nov 3;18(21):3650. doi: 10.3390/nu18213650 (PMC13765187; doi:10.3390/nu18213650)
Supplement: Supplementary file 1 [file nutrients-18-03650-s001.zip › Phase1_summaries.pdf]

SUMMARY FOR ARTICLE GROUP 1

Cluster Analysis Results: articles
Number of articles: 26
==

SYSTEMATIC UMBRELLA REVIEW CLUSTER SYNTHESIS

1. Overall Cluster Label: **Neurocognitive Mechanisms in Eating Disorders**

This cluster contains **two distinct subcategories**:
- **Primary subcategory (22 articles)**: Eating disorders research examining cognitive, neural, and physiological mechanisms
- **Secondary subcategory (4 articles)**: Transdiagnostic studies examining inhibitory control, gray matter alterations, and cognitive processes across multiple psychiatric conditions including eating disorders

2. Overall Findings Summary

Primary Eating Disorders Findings

Cognitive and Executive Function Deficits:
- **Inhibitory Control**: Moderate impairments across eating disorders (Iceta2021: β = -0.134, 95% CI [-0.262, -0.007] for BED; Cury2020: working memory SMD = -0.32, 95% CI [-0.60, -0.03])
- **Cognitive Flexibility**: Largest deficits in BED (Iceta2021: β = -0.399, 95% CI [-0.569, -0.229]; Colton2023: g = -0.21, 95% CI [-0.35, -0.07])
- **Decision-Making**: Mixed findings with uncertainty evaluation showing medium deficits in BED (Colton2023: g = -0.48, 95% CI [-0.77, -0.21])
- **Global Processing**: Significant deficits in both BDD (Lang2021: g = -0.44, 95% CI [-0.70, -0.17]) and AN (g = -0.63, 95% CI [-0.77, -0.49])

Neuroimaging Findings:
- **Structural Alterations**: Widespread gray matter reductions in AN, with largest effects in ENIGMA study (Walton2022: Cohen's d up to -0.95 for cortical thickness). Age-related vulnerability with younger patients showing greater reductions (Gao2025: -5.39% vs -3.09% in older patients)
- **Functional Alterations**: Distinct patterns between AN and BN during food processing (Bronleigh2021), with AN showing hypoactivation in reward regions and hyperactivation in cognitive control areas, while BN showed opposite patterns
- **Reward Processing**: Evidence for reward contamination rather than anhedonia in AN (Rye2025), with different patterns across illness

stages

Sensory and Interoceptive Processing:
- **Interoceptive Deficits**: Large effect sizes across all eating disorders (Cobbaert2024: AN d=1.57, BN d=2.61, BED d=1.96), with purging behaviors associated with greatest impairments
- **Sensory Processing**: Heightened external sensory sensitivity combined with impaired internal bodily awareness (Cobbaert2024)

Physiological Markers:
- **Heart Rate Variability**: No significant differences in BED (Christensen2023: g = 0.08, 95% CI [-0.62, 0.78]), but positive associations with disordered eating overall (Watford2020: r = 0.12, 95% CI [0.04, 0.29])
- **Anhedonia**: Large effect sizes in eating disorders vs controls (Dolan2022: g = 1.08, 95% CI [0.67, 1.49])

Transdiagnostic Findings

Inhibitory Control as Universal Deficit:
- Significant impairments across 8/10 psychiatric disorders (Breuer2024), with eating disorders showing medium effect (g=0.48, 95% CI [0.10-0.86])
- Distinct neural signatures: OCD showed ACC hypoactivation while eating disorders showed insula hypoactivation (Li2024)

Gray Matter Alterations:
- Novel methodology accounting for comorbidity revealed more precise disorder-specific patterns (Fortea2025), with anorexia showing highest effect intensity (g = 0.94)

3. Population Analysis

Gender Distribution:
- **Eating Disorders Studies**: Predominantly female (Articles 2,3,5,6,8,11,12,14,17,18,20,21,22,24,26: 87-100% female)
- **Mixed Gender Studies**: Articles 1,4,9,13,16,19,23 included both genders
- **Notable Gap**: Severe underrepresentation of males in eating disorders research

Socioeconomic Status:
- **Limited Reporting**: Only Articles 4,5,20,24 provided any SES data
- **Available Data**: Education years ranged 10.3-15.95 years (Article 4); one study noted 40% "upper middle class" (Article 8)

Race and Ethnicity:
- **Minimal Reporting**: Only Articles 8,15,24 provided race/ethnicity data
- **Available Data**: Predominantly White participants (75-93.6%

across reporting studies)
- **Geographic Bias**: Article 15 noted no studies from Asia, Africa, or Oceania

Demographic Gaps:
- Systematic underrepresentation of males, ethnic minorities, and non-Western populations
- Limited SES diversity reporting across all studies
- Age bias toward younger adults in most studies

4. Study Comparison

Similarities in Design and Methodology:
- **Meta-analytic Approach**: All 26 articles employed systematic review and meta-analysis methodology
- **Neuroimaging Focus**: Articles 2,7,9,10,14,17,18,21,22,23,26 used advanced neuroimaging meta-analysis techniques (ALE, SDM, VBM)
- **Quality Assessment**: Articles 5,11,15,16,20 employed rigorous quality assessment tools (Newcastle-Ottawa Scale, custom checklists)

Methodological Differences:
- **Sample Sizes**: Ranged from small (Article 3: n=277) to very large (Article 22: n=1,648; Article 1: n=13,807)
- **Statistical Approaches**: Traditional random-effects models (Articles 3,8,12) vs. advanced techniques like three-level meta-analysis (Article 11) and Bayesian modeling (Article 7)
- **Scope**: Single-disorder focus (Articles 3,6,17,22) vs. transdiagnostic approaches (Articles 1,4,9)

Convergent Findings:
- **Cognitive Impairments**: Articles 5,6,11,16,20 consistently found executive function deficits in eating disorders
- **Brain Structure**: Articles 9,10,18,21,22,23 converged on gray matter reductions in eating disorders
- **Sensory Processing**: Articles 4,7,12 supported altered sensory and interoceptive processing
- **Neural Dysfunction**: Articles 2,14,17,26 identified consistent limbic-prefrontal circuit alterations

Divergent Findings:
- **Heart Rate Variability**: Article 3 found no differences in BED while Article 24 found positive associations with disordered eating
- **Executive Functions**: Article 6 found minimal deficits while Articles 5,11 found significant impairments
- **Theory of Mind**: Article 15 found inconsistent deficits while Article 19 found preserved other-mentalization

Conclusion Similarities:
- Eating disorders involve multisystem dysfunction affecting cognitive, neural, and physiological processes

- Deficits are partially state-dependent and may improve with treatment
- Transdiagnostic approaches reveal shared mechanisms across psychiatric conditions

Conclusion Differences:
- Debate over whether deficits represent trait markers vs. state-dependent changes
- Disagreement on clinical utility of cognitive assessments and interventions
- Varying emphasis on disorder-specific vs. transdiagnostic treatment approaches

5. Article-Specific Information

Article 1 (Breuer2024)
- **Objective**: Examine inhibitory control as transdiagnostic dimension across mental disorders
- **Design**: Meta-analysis of 146 studies using antisaccade task
- **Population**: 13,807 participants across 10 diagnostic categories
- **Key Findings**: Significant elevations in 8/10 disorders; eating disorders g=0.48 (95% CI: 0.10-0.86)
- **Limitations**: Limited demographic diversity, cross-sectional design

Article 2 (Bronleigh2021)
- **Objective**: Neural correlates of food stimulus processing in eating disorders
- **Design**: ALE meta-analysis of 14 fMRI studies
- **Population**: 470 participants (AN, BN, healthy controls)
- **Key Findings**: Opposing neural patterns between AN (hypoactivation in reward regions) and BN (hyperactivation in reward regions)
- **Limitations**: Small number of studies, no separation of AN subtypes

Article 3 (Christensen2023)
- **Objective**: Heart rate variability in binge-eating disorder
- **Design**: Meta-analysis of 5 studies
- **Population**: 153 BED, 124 controls (all female)
- **Key Findings**: No significant difference (g = 0.08, 95% CI [-0.62, 0.78])
- **Limitations**: High heterogeneity (I^2=87%), methodological inconsistencies

Article 4 (Cobbaert2024)
- **Objective**: Sensory processing patterns across eating disorders
- **Design**: Meta-analysis of 19 studies
- **Population**: 6,382 participants across ED subtypes
- **Key Findings**: Large effect sizes for interoceptive difficulties

(AN: d=1.57, BN: d=2.61, BED: d=1.96)
- **Limitations**: Limited exteroception data, predominantly female samples

Article 5 (Colton2023)
- **Objective**: Decision-making dysfunction in binge-eating disorder
- **Design**: Meta-analysis of 50 studies using multi-stage framework
- **Population**: 4,088 participants
- **Key Findings**: Significant deficits in uncertainty evaluation (g = -0.48), attentional inhibition (g = -0.61), choice consistency (g = -0.61)
- **Limitations**: Limited demographic reporting, cross-sectional design

Article 6 (Cury2020)
- **Objective**: Executive functioning in binge eating disorder
- **Design**: Meta-analysis of 28 studies
- **Population**: 1,983 participants (87% female)
- **Key Findings**: Only working memory showed significant impairment (SMD = -0.32, 95% CI [-0.60, -0.03])
- **Limitations**: Small number of studies per domain, methodological heterogeneity

Article 7 (Datta2025)
- **Objective**: Interoception in eating disorders using novel Bayesian approach
- **Design**: Bayesian Author-Topic Model meta-analysis of 25 fMRI studies
- **Population**: 463 ED patients, 450 controls
- **Key Findings**: Three distinct neural components with cingulate cortex abnormalities
- **Limitations**: Novel methodology limits comparability, cross-sectional design

Article 8 (Dolan2022)
- **Objective**: Anhedonia in eating disorders
- **Design**: Meta-analysis of 21 studies
- **Population**: Predominantly female samples
- **Key Findings**: Large effect size (g = 1.08, 95% CI [0.67, 1.49]) for ED vs controls
- **Limitations**: Limited demographic diversity, potential publication bias

Article 9 (Fortea2025)
- **Objective**: Gray matter alterations accounting for psychiatric comorbidity
- **Design**: Novel multidisorder meta-analysis of 433 studies
- **Population**: 19,718 patients, 16,441 controls (51% female)
- **Key Findings**: Anorexia showed highest effect intensity (g = 0.94) when accounting for comorbidity

- **Limitations**: Cross-sectional design, limited demographic reporting

Article 10 (Gao2025)
- **Objective**: Age-related gray matter changes in anorexia nervosa
- **Design**: Age-stratified meta-analysis of 44 studies
- **Population**: 1,391 AN patients, 1,566 controls
- **Key Findings**: Younger patients showed greater GM reduction (−5.39% vs −3.09%)
- **Limitations**: Cross-sectional design, limited male representation

Article 11 (Iceta2021)
- **Objective**: Cognitive function in binge eating disorder and food addiction
- **Design**: Three-level meta-analysis of 42 studies
- **Population**: 3,973 participants
- **Key Findings**: Significant BED impairments in cognitive flexibility (β = −0.399), inhibitory control (β = −0.134)
- **Limitations**: Limited food addiction studies, predominantly female samples

Article 12 (Lang2021)
- **Objective**: Global visual processing in BDD and anorexia nervosa
- **Design**: Meta-analysis of 34 studies
- **Population**: 817 BDD participants, 1,075 AN participants, 883 controls
- **Key Findings**: Both disorders showed significant deficits (BDD: g = −0.44; AN: g = −0.63)
- **Limitations**: Cross-sectional design, task heterogeneity

Article 13 (Li2024)
- **Objective**: Inhibitory control in OCD and eating disorders using fMRI
- **Design**: SDM meta-analysis of 26 studies
- **Population**: 575 patients, 530 controls
- **Key Findings**: Distinct neural signatures (OCD: ACC hypoactivation; ED: insula hypoactivation)
- **Limitations**: Small number of ED studies, task heterogeneity

Article 14 (Norrlin2025)
- **Objective**: Neural correlates of body image processing in eating disorders
- **Design**: ALE meta-analysis of 14 fMRI studies
- **Population**: 556 participants (AN, BN, controls)
- **Key Findings**: Disorder-specific activation patterns in body estimation and satisfaction tasks
- **Limitations**: Task heterogeneity, small individual study samples

Article 15 (Preti2022)
- **Objective**: Theory of mind in eating disorders using RMET

- **Design**: Meta-analysis of 21 studies
- **Population**: 96% female across studies
- **Key Findings**: Inconsistent evidence for theory of mind deficits; only active AN showed some impairment
- **Limitations**: High heterogeneity, geographic bias, potential publication bias

Article 16 (Prunell-Castane2021)
- **Objective**: Executive functions across binge eating spectrum
- **Design**: Two separate meta-analyses (clinical BED vs subclinical)
- **Population**: Meta-analysis 1: 1,742 participants; Meta-analysis 2: 889 participants
- **Key Findings**: Small deficits in clinical BED (d = -0.22) but no association in subclinical samples
- **Limitations**: Limited statistical power for second meta-analysis, comorbidity confounds

Article 17 (Rye2025)
- **Objective**: Reward processing in restrictive anorexia nervosa
- **Design**: SDM meta-analysis of 18 datasets across illness stages
- **Population**: 339 AN-r patients, 307 controls (all female)
- **Key Findings**: Evidence for reward contamination rather than anhedonia; stage-specific patterns
- **Limitations**: All female samples, inconsistent recovery definitions

Article 18 (Sader2022)
- **Objective**: Structural brain differences between AN and ASD
- **Design**: ALE meta-analysis of 70 publications
- **Population**: 5,484 subjects across AN and ASD studies
- **Key Findings**: No neuroanatomical overlap between conditions despite behavioral similarities
- **Limitations**: Gender imbalances, cross-sectional design, methodological heterogeneity

Article 19 (Simonsen2020)
- **Objective**: Mentalization abilities in eating disorders
- **Design**: Meta-analysis of 44 studies
- **Population**: Predominantly female samples
- **Key Findings**: Significant self-mentalization deficits (SMD = 1.566, 95% CI [1.175-1.958])
- **Limitations**: High heterogeneity, limited high-quality studies

Article 20 (Stedal2021)
- **Objective**: Comprehensive neuropsychological functioning in adult AN
- **Design**: Multilevel meta-analysis of 50 studies
- **Population**: 1,778 AN patients, 2,279 controls (predominantly female)
- **Key Findings**: Moderate underperformance across all domains (g =

-0.43, 95% CI [-0.50, -0.36])
- **Limitations**: Limited male representation, methodological heterogeneity

Article 21 (Su2021)
- **Objective**: Multimodal brain alterations in anorexia nervosa
- **Design**: Combined VBM and resting-state meta-analysis
- **Population**: 1,085 AN patients, 1,201 controls (99%+ female)
- **Key Findings**: Converging structural and functional reductions in cingulate cortex
- **Limitations**: Cross-sectional design, predominantly female samples

Article 22 (Walton2022)
- **Objective**: Largest structural brain imaging study of anorexia nervosa
- **Design**: ENIGMA coordinated meta-analysis across 22 sites
- **Population**: 685 AN patients, 963 controls (all female)
- **Key Findings**: Largest psychiatric brain effects in ENIGMA (d up to -0.95); partial reversibility with weight restoration
- **Limitations**: Female-only samples, cross-sectional design

Article 23 (Wan2023)
- **Objective**: Common and distinct gray matter alterations in AN and OCD
- **Design**: SDM meta-analysis comparing disorders
- **Population**: 322 AN patients, 1,135 OCD patients, 1,636 controls
- **Key Findings**: Shared abnormalities in right insula, superior temporal gyrus, cognitive control regions
- **Limitations**: Gender imbalances between conditions, cross-sectional design

Article 24 (Watford2020)
- **Objective**: Heart rate variability and disordered eating behaviors
- **Design**: Meta-analysis of 31 studies
- **Population**: 1,898 participants (94% female, 75% Caucasian)
- **Key Findings**: Small positive association overall (r = 0.12); large effect in bulimia nervosa (r = 0.60)
- **Limitations**: Limited demographic diversity, methodological heterogeneity

Article 25 (Yu2023)
- **Objective**: Altered anticipatory brain responses in eating disorders
- **Design**: ALE meta-analysis of 21 fMRI studies
- **Population**: Predominantly female across studies
- **Key Findings**: Hyperactivation in right putamen, hypoactivation in left inferior parietal lobule
- **Limitations**: Predominantly female samples, task heterogeneity

Article 26 (Zhong2024)
- **Objective**: Comprehensive task-based fMRI alterations in anorexia nervosa
- **Design**: SDM meta-analysis across five task domains
- **Population**: All female participants across 82 studies
- **Key Findings**: Consistent limbic-prefrontal-cerebellar network dysfunction across task types
- **Limitations**: Female-only samples, cross-sectional design, task heterogeneity

SUMMARY FOR ARTICLE GROUP 2

Cluster Analysis Results: articles
Number of articles: 24
==

SYSTEMATIC UMBRELLA REVIEW CLUSTER SYNTHESIS: EATING DISORDER INTERVENTIONS

1. Overall Cluster Label
Eating Disorder Treatment Interventions Research

Subcategories Identified:
- **Psychotherapy Interventions** (CBT, Family-Based Therapy, DBT, Third-Wave Therapies)
- **Digital/Technology-Based Interventions** (eHealth, apps, virtual reality)
- **Specialized Approaches** (Cognitive Remediation, Multi-Family Therapy, Compassion-Focused Therapy)
- **Biomarker Research** (Ghrelin levels, neuroimaging)
- **Treatment Delivery Modalities** (Remote vs. in-person, higher levels of care)

2. Overall Findings Summary

Most Scientifically Relevant Findings:

Cognitive Behavioral Therapy Effectiveness:
- CBT shows robust effectiveness across eating disorders with large effect sizes: g = 0.88 (95% CI: 0.71–1.04) in controlled trials (Cuijpers2024) and g = 1.12 (95% CI: 0.98–1.25) in routine clinical care (Öst2023)
- CBT-T (brief 10-session format) demonstrates comparable effectiveness to standard 20-session treatment with very large effect sizes: g = −1.49 (95% CI: −1.68 to −1.31) for eating disorder psychopathology (Keegan2022)

Family-Based Treatment Superiority:
- FT-ED shows superior weight outcomes compared to individual therapy:

g = 0.46 (95% CI: 0.17–0.75) at end of treatment (Austin2024)
- Separated/parent-focused approaches outperform conjoint formats: g = −0.42 (95% CI: −0.73 to −0.11) for weight outcomes (Austin2024)
- Family therapy maintains effectiveness in routine clinical care with large effect sizes: g = 0.80 (95% CI: 0.69–0.91) for ED psychopathology (Wergeland2025)

Digital Intervention Efficacy:
- Technology-based interventions show moderate effectiveness: g = 0.58 (95% CI: 0.27–0.88) for global eating pathology (Haderlein2022)
- Internet-based CBT demonstrates significant benefits for BED: SMD = −0.77 (95% CI: −1.38 to −0.16) for binge episodes (Moghimi2021)
- Self-monitoring emerges as critical behavior change technique present in 100% of effective digital interventions (Thomas2024)

Most Clinically Relevant Findings:

Treatment Response Rates:
- CBT achieves 36% abstinence rates vs. 10% in controls, with Number Needed to Treat = 2.8 (Cuijpers2024)
- Multi-family therapy shows excellent acceptability with 86–100% completion rates (Zinser2022)
- Third-wave interventions demonstrate good acceptability with <18% dropout rates compared to ~30% for traditional treatments (Buerger2021)

Specialized Population Findings:
- Dialectical Behavior Therapy shows large effects on emotion regulation: g = −0.69 (95% CI: −1.22 to −0.16) particularly for general rather than eating-specific emotion regulation (Rozakou-Soumalia2021)
- Cognitive Remediation Therapy shows limited effectiveness for core AN symptoms but may reduce treatment dropout (Hagan2023, Alserihi2024)

Treatment Delivery Innovations:
- Remote CBT demonstrates equivalent effectiveness to in-person delivery: SMD = −0.02 (95% CI: −0.12 to 0.07) (Zandieh2024)
- FBT adaptations to higher levels of care show promising outcomes: 6% increase in expected body weight per month (Everhart2025)

3. Population Analysis

Gender Distribution:
Articles reporting gender data: Austin2024, Buerger2021, Cuijpers2024, Fuhrmann2024, Haderlein2022, Hagan2023, Hamid2023, Keegan2022, Low2020, Millard, Moghimi2021, Öst2023, Rozakou-Soumalia2021, Seidel2021, Thomas2024, Wang2024, Wergeland2025, Zandieh2024, Zhong2024, Zinser2022

Findings: Overwhelming female predominance across studies (90–100%

female in most studies). Only Fuhrmann2024 approached gender parity (49% female). Austin2024 notably included gender-diverse participants (2.4% transgender) in one study.

Socioeconomic Status:
Articles including SES data: Austin2024 (39% of included studies), Zinser2022 (limited reporting)

Notable gaps: 22 of 24 articles (92%) did not report SES data, representing a significant demographic bias limiting generalizability to diverse socioeconomic populations.

Race and Ethnicity:
Articles reporting race/ethnicity: Austin2024, Fuhrmann2024, Hamid2023, Keegan2022, Millard, Wang2024, Zinser2022

Findings: Predominantly White/Caucasian samples when reported. Millard reported 74.4% White participants across studies. Most studies conducted in Western countries (US, UK, Australia, Europe).

Notable demographic gaps: 17 of 24 articles (71%) did not adequately report race/ethnicity data, indicating significant representation bias toward Western, White populations.

4. Study Comparison

Similarities in Study Designs:
Shared methodological features (Articles 1-24): All articles employed systematic review and meta-analysis methodology following PRISMA guidelines. Common features include random-effects modeling, comprehensive database searches, and quality assessment using standardized tools (Cochrane Risk of Bias, GRADE).

Common outcome measures: Eating Disorder Examination Questionnaire (EDE-Q), body weight measures (%EBW, BMI), depression/anxiety scales, and dropout/completion rates across multiple articles (Austin2024, Cuijpers2024, Haderlein2022, Keegan2022, Öst2023, Wergeland2025).

Differences in Populations and Settings:
Age groups: Adolescent-focused studies (Austin2024, Buerger2021, Everhart2025, Wergeland2025) vs. adult-focused studies (Cuijpers2024, Haderlein2022, Hamid2023, Moghimi2021, Öst2023) vs. mixed-age studies (Zinser2022).

Treatment settings: Outpatient studies (Austin2024, Cuijpers2024, Keegan2022) vs. higher levels of care (Everhart2025) vs. mixed settings (Öst2023, Wergeland2025).

Diagnostic focus: Anorexia nervosa-specific (Alserihi2024, Austin2024, Hagan2023, Seidel2021, Zhong2024) vs. bulimia/binge eating

focus (Keegan2022, Low2020, Moghimi2021) vs. transdiagnostic approaches (Cuijpers2024, Öst2023, Thomas2024).

Convergent vs. Divergent Findings:

Convergent findings:
- **CBT effectiveness:** Cuijpers2024, Öst2023, Keegan2022, Haderlein2022, Hamid2023, and Moghimi2021 all support CBT's effectiveness across different delivery modalities and populations
- **Family therapy benefits:** Austin2024, Wergeland2025, Everhart2025, and Zinser2022 consistently demonstrate family-based approaches' effectiveness
- **Digital intervention promise:** Haderlein2022, Hamid2023, Moghimi2021, Thomas2024, and Fuhrmann2024 support technology-enhanced treatments

Divergent findings:
- **Cognitive Remediation Therapy:** Alserihi2024 and Hagan2023 both found limited effectiveness for core AN symptoms, contradicting earlier optimism about CRT
- **Third-wave therapies:** Buerger2021 found promising but limited evidence, while Rozakou-Soumalia2021 demonstrated significant effects for DBT, and Millard showed strong effects for CFT
- **Adjunctive interventions:** Fuhrmann2024 found no benefit for eating disorder apps, while other digital intervention studies showed positive effects

Similarities and Differences in Conclusions:

Similar conclusions:
- Need for improved demographic diversity in research (noted in 18/24 articles)
- Call for standardized outcome measures and longer follow-up periods (noted in 16/24 articles)
- Recognition that substantial proportions of patients remain unremitted even with effective treatments (Cuijpers2024, Moberg2022, Öst2023)

Different conclusions:
- **Treatment intensity:** Some studies support brief interventions (Keegan2022), while others emphasize longer-term approaches (Austin2024, Zinser2022)
- **Technology integration:** Optimistic conclusions about digital interventions (Thomas2024, Hamid2023) vs. cautious conclusions about app-based adjuncts (Fuhrmann2024)
- **Specialized approaches:** Mixed conclusions about cognitive remediation (Alserihi2024, Hagan2023) vs. strong support for emotion-focused approaches (Rozakou-Soumalia2021, Millard)

5. Article-Specific Information

Article 1: Alserihi2024
Research Question: Effectiveness of Cognitive Remediation Therapy for anorexia nervosa
Design: Systematic review and meta-analysis of 6 RCTs (n=413-556)
Population: Mixed gender (12% male), international samples
Key Findings: No significant improvements in cognitive flexibility (MD = -0.21, 95% CI [-0.44, 0.02]) or eating disorder symptoms (MD = -0.25, 95% CI [-0.76, 0.27])
Limitations: Language restrictions, lack of ethnic diversity reporting

Article 2: Austin2024
Research Question: Efficacy of family-based treatment for adolescent anorexia nervosa
Design: Systematic review and meta-analysis of 18 RCTs (n=1,138)
Population: Predominantly female adolescents, majority White samples
Key Findings: FT-ED superior to individual therapy for weight outcomes (g = 0.46, 95% CI [0.17, 0.75]); separated approaches superior to conjoint (g = -0.42, 95% CI [-0.73, -0.11])
Limitations: Inconsistent outcome measurement, limited demographic diversity

Article 3: Buerger2021
Research Question: Efficacy of third-wave interventions for adolescent eating disorders
Design: Systematic review and meta-analysis of 12 studies (n=487)
Population: 97.3% female, ages 11-21
Key Findings: Moderate effects on EDE total score (d = -0.67, 95% CI [-0.83, -0.59])
Limitations: Predominantly weak-quality studies, limited to DBT approaches

Article 4: Cuijpers2024
Research Question: Absolute and relative outcomes of CBT for adult eating disorders
Design: Meta-analysis of 36 trials (n=2,809)
Population: Predominantly female (>90% in most studies)
Key Findings: Large effect size (g = 0.88, 95% CI [0.71, 1.04]); 36% abstinence rate vs. 10% controls; NNT = 2.8
Subgroup Analyses: No significant difference between BED and BN; effects smaller in low risk of bias studies
Limitations: Limited AN representation, high heterogeneity

Article 5: Everhart2025
Research Question: FBT adaptations to higher levels of care for youth eating disorders
Design: Systematic review and meta-analysis of 35 studies
Population: Predominantly female, majority White samples

Key Findings: Weight restoration effect = 1.20 (95% prediction interval: 0.10, 2.30); EDE-Q symptoms effect = -0.61 per month
Limitations: Predominantly observational studies, limited diversity, lack of RCTs

Article 6: Fuhrmann2024
Research Question: Additive effects of adjunctive app-based interventions for mental disorders
Design: Meta-analysis of 46 RCTs (n=4,869)
Population: 49% female, mixed mental health conditions
Key Findings: Depression g = 0.17 (95% CI [0.02, 0.33]); eating disorders g = -0.02 (95% CI [-0.44, 0.40], non-significant)
Subgroup Analyses: Higher theoretical grounding associated with better outcomes
Limitations: 78% of studies had moderate-high risk of bias

Article 7: Haderlein2022
Research Question: Efficacy of technology-based interventions for threshold eating disorders
Design: Meta-analysis of 11 controlled trials (n=1,106)
Population: 98% female, mean age 31.75 years
Key Findings: Global eating pathology g = 0.58 (95% CI [0.27, 0.88]); objective binge episodes g = 0.72 (95% CI [0.31, 1.14])
Limitations: Limited AN representation, high heterogeneity

Article 8: Hagan2023
Research Question: Effectiveness of cognitive remediation therapy for anorexia nervosa
Design: Meta-analysis of 8 RCTs/quasi-RCTs
Population: 86.7-100% female across studies
Key Findings: Central coherence g = 0.25 (95% CI [-0.35, 0.85], non-significant); no significant differences in BMI or EDE scores
Limitations: Small sample sizes, heterogeneous measures

Article 9: Hamid2023
Research Question: Efficacy of Internet-based CBT for eating disorders
Design: Meta-analysis of 14 RCTs (n=2,076)
Population: 96.4-100% female, mixed ethnic reporting
Key Findings: Treatment studies showed large effects (0.30-1.11 SMD); prevention studies showed medium effects (0.31-0.47 SMD)
Limitations: Small evidence base, high heterogeneity, very low to low GRADE ratings

Article 10: Keegan2022
Research Question: Effectiveness of CBT-T (10-session CBT) for non-underweight eating disorders
Design: Meta-analysis of 10 studies (n=565)
Population: 77-97% female, predominantly Caucasian
Key Findings: Large effect sizes for ED psychopathology (g =

-1.49, 95% CI [-1.68, -1.31]); 65% good outcome rate; 39% dropout
Limitations: Limited controlled studies, heterogeneity in some outcomes

Article 11: Linardon2020
Research Question: E-mental health interventions for eating disorder treatment and prevention
Design: Meta-analysis of 36 RCTs (28 prevention, 8 treatment)
Population: Mixed, not specifically reported
Key Findings: Prevention trials: small-moderate effects (g = 0.19-0.43); Treatment trials: moderate-large effects (g = 0.29-0.69)
Limitations: Poor study quality, high dropout rates (21-25%), limited user-centered design

Article 12: Low2020
Research Question: VR-enhanced CBT vs. traditional CBT for binge-purging eating disorders
Design: Meta-analysis of 6 RCTs (n=297)
Population: Not reported
Key Findings: Significant reduction in binge frequency (MD = 0.29, 95% CI [0.01-0.57]) and situation-induced body dissatisfaction (SMD = 0.72, 95% CI [0.25-1.20])
Limitations: Limited studies, moderate-high risk of bias, insufficient follow-up

Article 13: Millard
Research Question: Effectiveness of Compassion Focused Therapy in clinical populations
Design: Meta-analysis of 15 studies (n=625)
Population: 74.88% female, 74.4% White, 9 countries
Key Findings: Self-compassion g = 1.12 (95% CI [0.61-1.63]); self-reassurance g = 0.51 (95% CI [0.19-0.83])
Subgroup Analyses: Group-based delivery most common; 84.6% retention rate
Limitations: Mixed quality ratings, gender imbalance, geographic concentration in Iran

Article 14: Moberg2022
Research Question: Comparison of CBT and psychodynamic-interpersonal therapy for eating disorders
Design: Meta-analysis of 62 studies, 76 samples
Population: Not reported
Key Findings: CBT remission rates: BED 50%, AN 33%, BN 28%; PIT showed lower rates with wide confidence intervals
Moderation Analyses: ED diagnosis predicted CBT outcomes (29% variance explained)
Limitations: Limited high-quality PIT studies, substantial non-remission rates

Article 15: Moghimi2021

Research Question: eHealth interventions for binge eating disorder
Design: Meta-analysis of 3 RCTs (n=298)
Population: 98.3% female, ages 35.1-40.2 years
Key Findings: Objective binge episodes SMD = -0.77 (95% CI [-1.38, -0.16]); EDE-Q total SMD = -0.71 (95% CI [-1.20, -0.22])
Limitations: Very small evidence base, high heterogeneity despite similar interventions

Article 16: Öst2023
Research Question: CBT effectiveness for adult eating disorders in routine clinical care
Design: Meta-analysis of 50 studies (n=4,299)
Population: 95.1% female, limited race/ethnicity reporting
Key Findings: Large within-group effects for ED psychopathology (g = 1.12, 95% CI [0.98-1.25]); comparable to efficacy studies
Subgroup Analyses: No significant differences between ED types; 25.5% dropout rate
Limitations: Considerable risk of bias, insufficient demographic reporting

Article 17: Rozakou-Soumalia2021
Research Question: DBT effectiveness for emotion dysregulation in eating disorders
Design: Meta-analysis of 11 studies (n=669)
Population: Predominantly female (85-100%), multiple countries
Key Findings: Overall emotion regulation g = -0.46 (95% CI [-0.67, -0.26]); general ER improved (g = -0.70) but not eating-specific ER
Subgroup Analyses: Waitlist controls showed larger effects than active therapy; BED studies showed larger effects
Limitations: High heterogeneity, limited generalizability beyond BED and female participants

Article 18: Seidel2021
Research Question: Ghrelin levels in anorexia nervosa as biomarker
Design: Meta-analysis of 49 studies (n=2,033 total)
Population: Primarily female (all but 2 studies), mean age 23.15 years
Key Findings: All ghrelin forms significantly elevated in acute AN: total ghrelin SMD = 2.51 (95% CI [1.68, 3.33]); longitudinal decrease during treatment but remained elevated
Limitations: High heterogeneity, variable assay methods, limited long-term follow-up

Article 19: Thomas2024
Research Question: Behavior change techniques in digital eating disorder interventions
Design: Meta-analysis of 17 RCTs examining digital interventions
Population: 53% all-female studies, poor ethnicity reporting (71% not mentioned)
Key Findings: Pooled effect size MD = -0.57 (95% CI [-0.80,

-0.39]); self-monitoring in 100% of effective interventions
Subgroup Analyses: Higher theoretical grounding associated with greater effects (high TCS = -0.86 vs. low TCS = -0.36)
Limitations: High heterogeneity, limited smartphone app evaluation

Article 20: Wang2024
Research Question: Written emotional disclosure interventions for eating disorders
Design: Meta-analysis of 13 RCTs (n=1,444)
Population: 74.4% female, predominantly White/European, university students
Key Findings: Eating disorder symptoms SMD = -0.20 (95% CI [-0.34, -0.05]); body dissatisfaction SMD = 0.37 (95% CI [0.21, 0.52])
Limitations: Limited to university populations, non-significant effects on general psychological distress

Article 21: Wergeland2025
Research Question: Family therapy and CBT effectiveness in routine clinical care for youth eating disorders
Design: Meta-analysis of 44 effectiveness studies (n=3,251)
Population: 94.1% female
Key Findings: Large effects for ED-psychopathology (g = 0.80, 95% CI [0.69-0.91]); CBT superior to family therapy (g = 1.05 vs 0.68)
Moderation Analyses: Older age associated with better outcomes; moderate risk of bias studies showed higher effects
Limitations: Considerable risk of bias across studies

Article 22: Zandieh2024
Research Question: Remote vs. in-person therapist-guided CBT across clinical conditions
Design: Meta-analysis of 54 RCTs (n=5,463)
Population: 61.4% female, multiple clinical conditions
Key Findings: No meaningful difference between delivery modalities: SMD = -0.02 (95% CI [-0.12, 0.07])
Subgroup Analyses: No credible subgroup effects by condition or therapy format
Limitations: Limited eating disorder-specific data within broader mental health focus

Article 23: Zhong2024
Research Question: Task-based fMRI patterns in anorexia nervosa
Design: Meta-analysis of 82 fMRI studies (n=3,857 total)
Population: 100% female across all studies
Key Findings: Consistent alterations across task domains in limbic system, prefrontal cortex, temporal regions, and cerebellum
Limitations: Female-only samples, no demographic diversity data, neuroimaging methodology limitations

Article 24: Zinser2022
Research Question: Multi-family therapy effectiveness for eating

disorders
Design: Meta-analysis of 15 studies (n=850 patients)
Population: 90-100% female, Western/European populations exclusively
Key Findings: Significant pre-post improvements: weight gain SMD = 0.85 (95% CI [0.46-1.25]); ED symptoms MD = -0.83 (95% CI [-1.23, -0.44])
Subgroup Analyses: Adolescents showed significant weight improvements (SMD = 1.15) while adults did not
Limitations: No superiority over active controls, limited to Western populations, variable intervention structures

SUMMARY FOR ARTICLE GROUP 3

Cluster Analysis Results: articles
Number of articles: 10
==

SYSTEMATIC UMBRELLA REVIEW CLUSTER SYNTHESIS

1. Overall Cluster Label
Eating Disorders Treatment and Prevention Interventions

Subcategories Identified:
- **Treatment Interventions** (6 studies): Early response prediction, low-intensity interventions, motivational interventions, self-guided interventions, intensive treatment settings, eHealth interventions
- **Prevention Programs** (2 studies): University-based prevention, eating disorder onset prevention
- **Treatment Predictors/Mechanisms** (2 studies): Early response patterns, pretreatment motivation

2. Overall Findings Summary

Treatment Effectiveness Findings

Early Response as Treatment Predictor (Chang2021):
- Moderate association between early response and treatment outcomes: **r = 0.41 [95% CI: 0.32-0.48]**
- Early response rate: 54.4% of participants by session 4
- Effect consistent across eating disorder types (AN, BN, BED) and treatment modalities

Low-Intensity Interventions (Davey2023):
- Equivalent to high-intensity therapy for eating disorder psychopathology: **g = -0.13 [95% CI: -0.30, 0.04]**
- Superior to non-eating disorder interventions: **g = -0.35 [95% CI: -0.49, -0.22]**
- Moderate effects vs. waitlist: **g = -0.68 [95% CI: -0.90, -0.46]**
- Lower remission rates compared to high-intensity therapy: **RR =

0.70 [95% CI: 0.56, 0.87]**

Self-Guided Interventions (Linardon2025_1):
- Most effective in pre-selected samples: **g = 0.42 [95% CI: 0.27, 0.56]** for eating disorder psychopathology
- Clinical samples showed moderate effects: **g = 0.40 [95% CI: 0.20, 0.60]**
- Small advantage for guided over unguided: **g = -0.26 [95% CI: -0.39, -0.13]**

Intensive Treatment Settings (deBoer2023):
- Large improvements in BMI for underweight patients: **g = -1.81 [95% CI: -2.32, -1.31]**
- Large improvements in disordered eating: **g = 0.97 [95% CI: 0.85, 1.09]**
- Large improvements in depression: **g = 0.94 [95% CI: 0.79, 1.08]**
- Residential treatment superior to day programs and inpatient settings

eHealth Interventions for BED (Tuncer2025):
- Moderate effects on binge eating psychopathology: **SMD = 0.53 [95% CI: 0.20-0.86]**
- Moderate effects on objective binge eating days: **SMD = 0.49 [95% CI: 0.12-0.85]**

Ineffective Interventions:
- Motivational interventions showed no significant effects across all outcomes (Fetahi2022): motivation **SMD = 0.03 [95% CI: -0.25, 0.31]**, eating disorder psychopathology **SMD = 0.02 [95% CI: -0.20, 0.24]**

Prevention Effectiveness Findings

University Prevention Programs (Harrer2020):
- 38% reduction in eating disorder incidence: **IRR = 0.62 [95% CI: 0.44, 0.87]**
- Moderate effects on global eating disorder symptoms: **g = 0.35 [95% CI: 0.24, 0.46]**
- Strong effects on dieting behaviors: **g = 0.43 [95% CI: 0.29, 0.57]**

Eating Disorder Onset Prevention (Stice2021):
- Significant prevention effects: **OR = 1.64 [95% CI: 1.09, 2.46]**
- Single risk factor recruitment more effective: **OR = 2.00 [95% CI: 1.31, 3.06]**
- Healthy lifestyle modification programs superior: **OR = 2.93 [95% CI: 1.52, 5.67]**
- Peer-led interventions more effective than clinician-led: **OR = 2.70 [95% CI: 1.29, 5.67]**

Secondary Mental Health Benefits

Self-Help Mental Health Effects (Linardon2025_2):
- Small but significant effects on depression: **g = 0.24 [95% CI: 0.15-0.34]**
- Small effects on anxiety: **g = 0.23 [95% CI: 0.15-0.32]**
- Effects most robust in pre-selected samples

Treatment Motivation as Predictor (Sansfacon2020):
- Small but significant association with overall ED symptoms: **r = 0.17 [95% CI: 0.09-0.24]**
- Moderate-large effect on binge-eating: **r = 0.48 [95% CI: 0.38-0.58]**
- No association with anxiety/depression outcomes

3. Population Analysis

Gender Distribution
- **Predominantly female samples across all studies**
- **Articles with gender data:** Chang2021 (nearly all female), Davey2023 (>75% female in most studies), Fetahi2022 (885 women, 31 men), Harrer2020 (88.9% female-only studies), Linardon2025_2 (51-100% female), Sansfacon2020 (79-100% female), Stice2021 (13/15 trials female-only), Tuncer2025 (86-98.6% female), deBoer2023 (90.4% female)
- **Articles lacking gender data:** Linardon2025_1

Socioeconomic Status
- **Articles with SES data:** Linardon2025_2 (varied education/ employment levels), deBoer2023 (limited income data from one study)
- **Articles lacking SES data:** Chang2021, Davey2023, Fetahi2022, Harrer2020, Linardon2025_1, Sansfacon2020, Stice2021, Tuncer2025

Race and Ethnicity
- **Articles with race/ethnicity data:** Harrer2020 (23-92% White), Stice2021 (65.2% White, 13.1% Hispanic/Latino), Sansfacon2020 (92-97% Caucasian in individual studies), deBoer2023 (71-96% White)
- **Articles lacking race/ethnicity data:** Chang2021, Davey2023, Fetahi2022, Linardon2025_1, Linardon2025_2, Tuncer2025

Notable Demographic Gaps
- **Severe underrepresentation of males** across all intervention types
- **Limited racial/ethnic diversity** in most studies
- **Minimal SES reporting** despite known socioeconomic barriers to eating disorder treatment
- **Geographic bias** toward Western, developed countries

4. Study Comparison

Methodological Similarities

- **All studies used systematic review and meta-analysis methodology**
- **Shared features:** Chang2021, Davey2023, Fetahi2022, Harrer2020, Linardon2025_1, Linardon2025_2, Sansfacon2020, Stice2021, Tuncer2025, deBoer2023
- **Random-effects models predominant** across analyses
- **PROSPERO registration common** (Harrer2020, deBoer2023, Chang2021)
- **Comprehensive database searches** across multiple platforms

Population and Setting Differences
- **University populations:** Harrer2020, Stice2021 (prevention focus)
- **Clinical populations:** Chang2021, deBoer2023, Tuncer2025 (intensive treatment focus)
- **Mixed populations:** Davey2023, Linardon2025_1, Linardon2025_2 (prevention through treatment continuum)
- **Specific disorder focus:** Tuncer2025 (BED only) vs. others (multiple eating disorders)

Convergent Findings
- **Self-help interventions effective:** Supported by Davey2023, Linardon2025_1, Linardon2025_2, Tuncer2025
- **Prevention programs work:** Confirmed by Harrer2020, Stice2021
- **Treatment intensity matters:** Supported by Davey2023 (low-intensity effective), deBoer2023 (intensive treatment effective)
- **Early intervention important:** Supported by Chang2021, Harrer2020, Stice2021

Divergent Findings
- **Motivational interventions:** Fetahi2022 found no effects, contradicting individual studies
- **Guided vs. unguided interventions:** Linardon2025_1 found small differences, while Tuncer2025 focused only on guided approaches
- **Treatment setting effects:** deBoer2023 found residential superior, while other studies showed equivalence across settings

Conclusion Similarities
- **Stepped-care models supported** by multiple studies
- **Accessibility and scalability emphasized** across intervention types
- **Need for early intervention** consistently highlighted
- **Digital delivery viable** across multiple studies

Conclusion Differences
- **Resource allocation:** Some studies favor intensive treatment (deBoer2023) while others emphasize low-intensity approaches (Davey2023, Linardon2025_1)
- **Target populations:** Prevention studies emphasize universal/selective approaches while treatment studies focus on clinical populations
- **Implementation priorities:** Vary from peer-led prevention (Stice2021) to professional-guided treatment (deBoer2023)

5. Article-Specific Information

Chang2021
- **Objective:** Examine early response as predictor of eating disorder treatment outcomes
- **Design:** Meta-analysis of 33 studies (25 in meta-analysis, N=2,740)
- **Population:** Predominantly female, multiple eating disorder types
- **Key Findings:** Moderate association r = 0.41 [95% CI: 0.32-0.48], consistent across disorders
- **Limitations:** High heterogeneity (I^2 = 80.2%), limited demographic diversity

Davey2023
- **Objective:** Evaluate low-intensity psychological interventions for eating disorders
- **Design:** Meta-analysis of 33 RCTs (N=3,665)
- **Population:** Predominantly female across age ranges
- **Key Findings:** Equivalent to high-intensity therapy for symptoms, superior to non-specific interventions
- **Subgroup Analyses:** Effects varied by comparison condition, strongest vs. waitlist
- **Limitations:** Limited data for children/adolescents and restrictive eating disorders

Fetahi2022
- **Objective:** Assess effectiveness of motivational interventions in eating disorders
- **Design:** Meta-analysis of 13 RCTs (N=1,322)
- **Population:** 885 women, 31 men
- **Key Findings:** No significant effects on motivation, eating disorder psychopathology, or BMI
- **Moderation Analyses:** Age, BMI, sample size showed no moderation effects (all R^2 = 0)
- **Limitations:** High study heterogeneity, moderate-high risk of bias across studies

Harrer2020
- **Objective:** Examine eating disorder prevention programs in university students
- **Design:** Meta-analysis of 27 RCTs (N=4,856)
- **Population:** 88.9% female-only studies, university students
- **Key Findings:** 38% reduction in eating disorder incidence, moderate symptom effects
- **Subgroup Analyses:** Selective prevention more effective than universal
- **Limitations:** Moderate heterogeneity, limited male representation

Linardon2025_1

- **Objective:** Evaluate self-guided psychological interventions across prevention-treatment continuum
- **Design:** Meta-analysis of 46 trials across three sample types
- **Population:** Varied across unselected, pre-selected, and clinical samples
- **Key Findings:** Most effective in pre-selected samples (g = 0.42), small guided advantage
- **Moderation Analyses:** Waitlist controls and intervention type moderated effects
- **Limitations:** Limited demographic reporting, heterogeneous interventions

Linardon2025_2
- **Objective:** Examine mental health impacts of eating disorder self-help interventions
- **Design:** Meta-analysis of 27 RCTs examining secondary mental health outcomes
- **Population:** Predominantly female (51-100%), diverse ethnic representation
- **Key Findings:** Small but significant effects on depression, anxiety, distress in pre-selected samples
- **Subgroup Analyses:** Effects robust across intervention types and delivery methods
- **Limitations:** Predominant use of waitlist controls, small effect sizes

Sansfacon2020
- **Objective:** Quantify relationship between pretreatment motivation and therapy outcomes
- **Design:** Meta-analysis of 42 studies using combined probability and effect size analyses
- **Population:** Predominantly female across diverse treatment settings
- **Key Findings:** Small but significant association with overall ED symptoms (r = 0.17)
- **Moderation Analyses:** Effects varied by outcome type, strongest for binge-eating
- **Limitations:** Publication bias, methodological quality variations

Stice2021
- **Objective:** Examine prevention programs' effects on eating disorder onset
- **Design:** Meta-analysis of 15 RCTs (N=5,080)
- **Population:** 13/15 trials female-only, diverse ethnic representation
- **Key Findings:** Significant prevention effects (OR = 1.64), peer-led programs superior
- **Subgroup Analyses:** Single risk factor recruitment and lifestyle modification most effective
- **Limitations:** Limited male inclusion, heterogeneous intervention

approaches

Tuncer2025
- **Objective:** Evaluate eHealth-based guided self-help interventions for BED
- **Design:** Meta-analysis of 8 RCTs (N=1,575)
- **Population:** 86-98.6% female, adults with BED
- **Key Findings:** Moderate effects on binge eating psychopathology (SMD = 0.53) and frequency
- **Limitations:** High heterogeneity (I^2 = 89-91%), limited to BED only

deBoer2023
- **Objective:** Examine effectiveness of intensive eating disorder treatments
- **Design:** Meta-analysis of 62 studies (N=13,137)
- **Population:** 90.4% female, predominantly Caucasian, adult clinical samples
- **Key Findings:** Large improvements across BMI, eating symptoms, depression, quality of life
- **Moderation Analyses:** Residential treatment and longer stays (>94.4 days) superior
- **Limitations:** High heterogeneity (I^2 = 80.6-97.9%), predominantly naturalistic studies, publication bias detected

SUMMARY FOR ARTICLE GROUP 4

Cluster Analysis Results: articles
Number of articles: 9
==

SYNTHESIZED CLUSTER ANALYSIS: PSYCHOLOGICAL FACTORS IN EATING DISORDERS

1. Overall Cluster Label
Psychological Risk Factors in Eating Disorders

Subcategories Identified:
- **Perfectionism and Self-Criticism** (Articles 4, 5, 7, 8)
- **Body-Related Psychological Factors** (Articles 1, 2, 3, 6)
- **Self-Compassion as Protective Factor** (Articles 7, 9)

2. Overall Findings Summary

Primary Associations with Eating Disorders:

Perfectionism:
- **Adults**: Perfectionistic concerns r = 0.33 (95% CI: 0.30-0.37), perfectionistic strivings r = 0.20 (95% CI: 0.14-0.25) (Stackpole2023)
- **Youth**: Perfectionistic concerns r = 0.31 (95% CI: 0.26-0.36),

perfectionistic strivings r = 0.21 (95% CI: 0.15-0.26) (Bills2023)
- **Clinical samples**: Stronger effects for both dimensions in clinical populations (Stackpole2023, Bills2023)

Self-Criticism and Shame:
- **Self-criticism**: Overall r = 0.37 (95% CI: 0.36-0.42), with strongest effects for SCS self-criticism subscale r = 0.52 (Paranjothy2024)
- **Shame**: Very large association r = 0.498 (95% CI: 0.474-0.522), with body shame r = 0.546 and eating-related shame r = 0.589 showing strongest effects (Nechita2021)

Body-Related Psychological Factors:
- **Social physique anxiety**: Very large effect r = 0.51 (95% CI: 0.475-0.539) (Alcaraz-Ibanez2023)
- **Morbid exercise behavior**: Medium effect r = 0.35 (95% CI: 0.30-0.40) overall, with dietary restraint showing strongest association r = 0.42 (95% CI: 0.34-0.49) (Alcaraz-Ibanez2020)
- **Disgust**: Self-disgust showed very large effect g = 1.90 (95% CI: 1.51-2.29), food-related disgust g = 1.32 (95% CI: 1.05-1.59) (Bektas2022)

Protective Factors:
- **Self-compassion**: Medium negative association with eating pathology r = -0.34 (95% CI: -0.40 to -0.28) and body image concerns r = -0.45 (95% CI: -0.50 to -0.39) (Turk2020)

Treatment Efficacy:
- **CBT for perfectionism**: Large effects on perfectionism measures (g = 0.57-0.89) and medium effects on eating disorders g = 0.61 (95% CI: 0.36-0.87) (Galloway2021)
- **Self-compassion interventions**: Medium effects for eating pathology g = 0.58 (95% CI: 0.16-1.00) (Turk2020)

Most Clinically Relevant Findings:
Self-disgust (g = 1.90) and eating-related shame (r = 0.589) showed the largest effect sizes, suggesting these may be priority treatment targets. Cultural factors moderate social physique anxiety effects, with stronger associations in Western countries.

3. Population Analysis

Gender Distribution:
- **Articles reporting gender data**: All 9 articles
- **Predominant pattern**: Heavily female-skewed across all studies (82-95% female in most samples)
- **Male representation**: Limited across all studies; only Bills2023 and Stackpole2023 included meaningful male samples
- **Notable gaps**: Severe underrepresentation of males and non-binary individuals

Socioeconomic Status:
- **Articles including SES data**: Only Nechita2021 (noted "mostly educated, middle-class")
- **Major limitation**: 8 of 9 articles did not report SES data

Race and Ethnicity:
- **Articles reporting race/ethnicity**: Only Nechita2021 and Paranjothy2024
- **Predominant pattern**: Where reported, 75-76% White participants
- **Geographic diversity**: Studies conducted across Western countries primarily (USA, Canada, Europe, Australia)
- **Major gap**: Limited representation of non-Western populations and ethnic minorities

4. Study Comparison

Similarities in Design and Methodology:
- **Meta-analytic approach**: All 9 articles used systematic review and meta-analysis
- **Random effects models**: Consistently used across studies (Articles 1-9)
- **PRISMA compliance**: Most studies followed PRISMA guidelines (Articles 2, 3, 4, 5, 7, 8, 9)
- **Quality assessment**: Systematic quality evaluation of included studies (Articles 3, 4, 5, 7, 8, 9)

Differences in Populations and Settings:
- **Age focus**: Bills2023 examined youth (0-18 years), Stackpole2023 focused on adults, others included mixed ages
- **Clinical vs. non-clinical**: Alcaraz-Ibanez2020, Bills2023, Stackpole2023 examined clinical status as moderator; others primarily non-clinical samples
- **Sample sizes**: Ranged from 912 (Galloway2021) to 64,267 participants (Nechita2021)

Convergent Findings:
- **Consistent medium-large effects**: All psychological risk factors showed significant positive associations with eating disorders
- **Clinical sample effects**: Studies examining clinical populations found stronger associations (Alcaraz-Ibanez2020, Bills2023, Stackpole2023)
- **Age moderation**: Younger individuals showed stronger associations across multiple studies (Alcaraz-Ibanez2020, Nechita2021)
- **Gender effects**: Where examined, females showed stronger associations (Alcaraz-Ibanez2020)

Divergent Findings:
- **Effect size magnitude**: Self-disgust and shame showed largest effects ($g = 1.90$, $r = 0.589$) while perfectionism showed more moderate

effects (r = 0.20–0.37)
- **Cultural differences**: Only Alcaraz-Ibanez2023 found significant cultural moderation (Western vs. non-Western countries)
- **Measurement effects**: Alcaraz-Ibanez2020 found substantial variation by assessment instrument, while other studies found more consistent effects across measures

Conclusion Similarities:
All studies concluded that psychological factors represent important transdiagnostic mechanisms in eating disorders warranting clinical attention and intervention development.

Conclusion Differences:
- **Treatment implications**: Galloway2021 and Turk2020 provided direct intervention evidence, while others focused on risk factor identification
- **Developmental considerations**: Bills2023 emphasized early intervention, while adult-focused studies emphasized maintenance factors
- **Cultural considerations**: Only Alcaraz-Ibanez2023 emphasized cultural adaptation of interventions

5. Article-Specific Information

Article 1: Alcaraz-Ibanez2020
- **Objective**: Meta-analyze relationships between morbid exercise behavior and eating disorders
- **Design**: Meta-analysis of 66 studies (N = 21,816)
- **Population**: Mixed gender, primarily non-clinical samples
- **Primary findings**: Overall r = 0.35 (95% CI: 0.30–0.40); dietary restraint r = 0.42 (95% CI: 0.34–0.49)
- **Subgroup analyses**: Clinical samples showed larger effects (r = 0.60 vs 0.33); age and BMI moderated relationships
- **Limitations**: Cross-sectional data, limited clinical samples, measurement inconsistencies

Article 2: Alcaraz-Ibanez2023
- **Objective**: Examine social physique anxiety-eating disorder relationships
- **Design**: Meta-analysis of 69 studies with 170 effect sizes (N = 41,257)
- **Population**: Mixed gender (predominantly female), international samples
- **Primary findings**: Very large effect r = 0.51 (95% CI: 0.475–0.539)
- **Subgroup analyses**: Stronger effects in Western countries (r = 0.593) vs non-Western (r = 0.370)
- **Limitations**: Cross-sectional data, unbalanced representation across ED types and regions

Article 3: Bektas2022
- **Objective**: Systematic review and meta-analysis of disgust in eating disorders
- **Design**: Meta-analysis of 19 studies across three disgust domains
- **Population**: Predominantly female ED patients vs. healthy controls
- **Primary findings**: Self-disgust g = 1.90 (95% CI: 1.51–2.29); food-related disgust g = 1.32 (95% CI: 1.05–1.59)
- **Subgroup analyses**: Consistent effects across AN and BN diagnoses
- **Limitations**: Limited male representation, few studies for some ED diagnoses

Article 4: Bills2023
- **Objective**: First meta-analysis of perfectionism-eating disorder relationships in youth
- **Design**: Meta-analysis of 39 studies (N = 13,954)
- **Population**: Children and adolescents (0–18 years), 82% female
- **Primary findings**: Perfectionistic concerns r = 0.31 (95% CI: 0.26–0.36); perfectionistic strivings r = 0.21 (95% CI: 0.15–0.26)
- **Subgroup analyses**: Clinical status moderated perfectionistic strivings
- **Limitations**: High heterogeneity, predominantly cross-sectional, limited diversity

Article 5: Galloway2021
- **Objective**: Evaluate efficacy of CBT for perfectionism interventions
- **Design**: Meta-analysis of 15 RCTs (N = 912)
- **Population**: 82% female, mean age 23 years
- **Primary findings**: Large effects on perfectionism (g = 0.57–0.89); medium effect on eating disorders g = 0.61 (95% CI: 0.36–0.87)
- **Subgroup analyses**: Face-to-face and self-help formats equally effective
- **Limitations**: Small number of face-to-face studies, limited clinical samples

Article 6: Nechita2021
- **Objective**: Comprehensive meta-analysis of shame-eating disorder relationships
- **Design**: Meta-analysis of 195 studies with 243 samples (N = 64,267)
- **Population**: >95% female, predominantly White, educated samples
- **Primary findings**: Overall r = 0.498 (95% CI: 0.474–0.522); body shame r = 0.546; eating-related shame r = 0.589
- **Subgroup analyses**: Age negatively moderated relationships; type of shame significantly moderated effects
- **Limitations**: Cross-sectional data, limited diversity, poor study quality

Article 7: Paranjothy2024
- **Objective**: First meta-analysis of self-criticism, self-compassion, and disordered eating relationships
- **Design**: Meta-analysis of 135 studies (N = 42,952)
- **Population**: 87.68% female, 75.66% White (where reported)
- **Primary findings**: Self-criticism r = 0.37 (95% CI: 0.36–0.42); self-compassion r = -0.42 (95% CI: -0.54 to -0.36)
- **Subgroup analyses**: Self-criticism measures stronger than perfectionism measures; longitudinal effects maintained
- **Limitations**: High heterogeneity, predominantly cross-sectional, limited diversity

Article 8: Stackpole2023
- **Objective**: Meta-analyze perfectionism-eating disorder relationships in adults
- **Design**: Meta-analysis of 95 studies (N = 32,840)
- **Population**: 43% female-only studies, international samples
- **Primary findings**: Perfectionistic concerns r = 0.33 (95% CI: 0.30–0.37); perfectionistic strivings r = 0.20 (95% CI: 0.14–0.25)
- **Subgroup analyses**: Clinical samples showed stronger effects for both dimensions
- **Limitations**: Small number of clinical studies, publication bias, high heterogeneity

Article 9: Turk2020
- **Objective**: Examine self-compassion relationships and intervention effects for eating/body image concerns
- **Design**: Meta-analysis of 59 studies examining correlations and interventions
- **Population**: Predominantly female, international samples
- **Primary findings**: Self-compassion negatively correlated with eating pathology r = -0.34 (95% CI: -0.40 to -0.28); intervention effects g = 0.58 (95% CI: 0.16–1.00)
- **Subgroup analyses**: Study quality, age, and BMI moderated some relationships
- **Limitations**: Heavily female samples, variable intervention approaches

SUMMARY FOR ARTICLE GROUP 5

Cluster Analysis Results: articles
Number of articles: 9
==

SYSTEMATIC UMBRELLA REVIEW CLUSTER ANALYSIS

1. Overall Cluster Label
Eating Disorders Treatment and Recovery Outcomes

Subcategories Identified:

- **Screening and Help-Seeking Studies** (Ali2024, Feltner2022, Kutz2020, Radunz2022)
- **Treatment Outcomes and Recovery** (Solmi2024, Chaer2020, Kan2021)
- **Relapse Prediction Studies** (Rijk, Sala2023)

2. Overall Findings Summary

Most Scientifically Relevant Findings:

Screening Accuracy:
- SCOFF questionnaire demonstrates pooled sensitivity of 84% (95% CI: 74-90%) and specificity of 80% (95% CI: 65-89%) in adults (Feltner2022), with overall diagnostic accuracy AUC = 0.91 (95% CI: 0.88-0.93) (Kutz2020)

Help-Seeking Barriers:
- Only 30% (95% CI: 22-38%) of individuals with eating disorders seek any help, with 32% (95% CI: 24-41%) seeking formal treatment, indicating **68% unmet treatment need** (Ali2024)
- Two primary barriers significantly predict reduced help-seeking: denial/failure to perceive severity (r = -0.19, 95% CI: -0.33 to -0.05) and perceived inability of others to help (r = -0.15, 95% CI: -0.30 to -0.01) (Radunz2022)

Treatment Outcomes:
- Overall recovery rate across eating disorders: **46% (95% CI: 44-49%)**, improving from 42% at <2 years to 67% at ≥10 years follow-up (Solmi2024)
- Mortality rate: 0.4% (95% CI: 0.2-0.7) overall, with 5.2 deaths/ 1,000 person-years in observational studies (Solmi2024)

Most Clinically Relevant Findings:

Relapse Predictors:
- **Post-treatment BMI** (SMD = -0.35, 95% CI: -0.63 to -0.07) and **pre-treatment depression** (SMD = 0.40, 95% CI: 0.21-0.59) are the most consistent predictors of anorexia nervosa relapse (Rijk)
- Average relapse rate across eating disorders: **36-37%** (Rijk, Sala2023)

Treatment Effectiveness:
- **Lisdexamfetamine** for binge eating disorder: mean difference -5.75 (95% CI: -8.32 to -3.17) on YBOCS-BE scale (Feltner2022)
- **Guided self-help** shows significant improvement: SMD -0.96 (95% CI: -1.26 to -0.67) (Feltner2022)
- **Children/adolescents have significantly better outcomes** than adults across all measures (Solmi2024)

Recovery Outcomes:
- Reproductive function normalizes after anorexia nervosa recovery

with no significant difference in childbirth rates compared to general population (OR = 0.75, 95% CI: 0.43–1.29, p = 0.41) (Chaer2020)

3. Population Analysis

Gender Distribution:
- **Articles reporting gender data:** All 9 articles
- **Pattern:** Consistently female-predominant across all studies (ranging from 51–100% female, with most >75% female)
- **Notable:** Only Kan2021 included 2 male-only studies; Chaer2020 was female-only by design

Socioeconomic Status:
- **Articles including SES data:** Limited reporting across studies
- **Specific mentions:** Rijk (middle-high SES when reported), others mostly "not reported"
- **Gap identified:** Systematic under-representation of diverse SES populations

Race and Ethnicity:
- **Articles reporting race/ethnicity:** Ali2024, Feltner2022, Kutz2020, Radunz2022, Sala2023
- **Pattern:** Predominantly White/Caucasian samples (64.6–98% when reported)
- **Most diverse:** Ali2024 included one study with 72.4% ethnic-racial minority participants
- **Significant gap:** Consistent under-representation of racial/ethnic minorities across all study types

Notable Demographic Biases:
- Systematic exclusion of males, racial/ethnic minorities, and diverse SES populations
- Geographic concentration in high-income Western countries
- Limited adolescent representation except in Solmi2024

4. Study Comparison

Similarities in Design and Methodology:
- **Systematic review methodology:** All 9 articles used rigorous systematic review approaches with PRISMA guidelines
- **Meta-analytic techniques:** 8/9 studies employed meta-analysis (Chaer2020 used narrative synthesis)
- **Quality assessment:** All studies included formal quality assessment of included studies
- **Database searches:** Consistent use of PubMed/MEDLINE, PsycINFO, and additional databases

Differences in Populations and Settings:
- **Geographic focus:** Kan2021 emphasized international variation; others primarily Western samples

- **Age ranges:** Solmi2024 and Feltner2022 included adolescents; others focused on adults
- **Clinical settings:** Kan2021 (inpatient), Feltner2022 (primary care screening), others (mixed settings)
- **Diagnostic focus:** Solmi2024 and Sala2023 (all EDs), others (specific disorders or populations)

Convergent Findings:
- **Supported by multiple studies:** Low help-seeking rates (Ali2024, Radunz2022), high relapse rates ~37% (Rijk, Sala2023), female predominance across all studies
- **Treatment effectiveness:** Multiple studies support CBT and guided self-help effectiveness
- **Recovery patterns:** Longer follow-up associated with better outcomes (Solmi2024, Chaer2020)

Divergent Findings:
- **SCOFF accuracy:** Kutz2020 found excellent accuracy (AUC=0.91) vs. Feltner2022 showing more modest performance in diverse populations
- **Treatment duration:** Kan2021 showed dramatic international variation (US: 49 days vs. Europe: 106 days)
- **Recovery definitions:** Significant heterogeneity in outcome definitions across studies

Conclusion Similarities:
- All studies emphasize need for earlier intervention
- Consistent calls for more diverse, representative samples
- Recognition of significant treatment gaps and barriers
- Emphasis on evidence-based treatment approaches

Conclusion Differences:
- **Screening recommendations:** Kutz2020 cautious about SCOFF use vs. Feltner2022 supporting its utility
- **Treatment focus:** Some emphasize psychological interventions, others highlight pharmacological options
- **Research priorities:** Vary from screening implementation to relapse prevention to treatment accessibility

5. Article-Specific Information

Article 1 (Ali2024)
- **Objective:** Update 2011 review on unmet treatment need for eating disorders
- **Design:** Systematic review and meta-analysis of 21 studies (N=37,423)
- **Population:** Community samples, predominantly female (51-100%)
- **Key Findings:** 30% help-seeking rate (95% CI: 22-38%), indicating 68% unmet need
- **Limitations:** Significant publication bias, high heterogeneity (I^2>99%)

Article 2 (Chaer2020)
- **Objective:** Examine fertility outcomes after anorexia nervosa recovery
- **Design:** Systematic review and meta-analysis of 5 longitudinal studies (N=353)
- **Population:** Females of fertile age, 6-18 year follow-up
- **Key Findings:** No significant difference in childbirth rates vs. general population (OR=0.75, 95% CI: 0.43-1.29)
- **Limitations:** Small sample sizes, unclear recovery status in some participants

Article 3 (Feltner2022)
- **Objective:** USPSTF evidence review on eating disorder screening
- **Design:** Systematic review of 57 studies (N=10,773)
- **Population:** Adolescents and adults, predominantly female and White
- **Key Findings:** SCOFF sensitivity 84% (95% CI: 74-90%), specificity 80% (95% CI: 65-89%)
- **Subgroup Findings:** Lisdexamfetamine effective for BED (-5.75, 95% CI: -8.32 to -3.17)
- **Limitations:** No direct screening benefit evidence, limited adolescent data

Article 4 (Kan2021)
- **Objective:** Determine global length of stay patterns for anorexia nervosa treatment
- **Design:** Meta-analysis of 71 studies (111 datasets, N=3,258)
- **Population:** Predominantly female (78-100%), global sample
- **Key Findings:** Pooled mean LOS = 76.3 days (95% CI: 73.3-79.4)
- **Subgroup Findings:** Europe 105.6 days vs. US 49.1 days; Age β=3.25 days/year
- **Limitations:** High heterogeneity (I^2=99.6%), publication bias evident

Article 5 (Kutz2020)
- **Objective:** Evaluate SCOFF diagnostic accuracy for DSM-5 eating disorders
- **Design:** Meta-analysis of 25 validation studies (N=11,531)
- **Population:** Varied gender distribution, 13 countries, primarily Caucasian
- **Key Findings:** Sensitivity 0.86 (95% CI: 0.78-0.91), specificity 0.83 (95% CI: 0.77-0.88), AUC=0.91
- **Subgroup Findings:** Reduced effectiveness for BED and male populations
- **Limitations:** High heterogeneity (I^2>97%), limited diversity in validation samples

Article 6 (Radunz2022)
- **Objective:** Identify factors associated with eating disorder

help-seeking behavior
- **Design:** Meta-analysis of 19 studies examining 24 unique themes
- **Population:** 52.4–100% female, varied racial/ethnic composition
- **Key Findings:** Only denial (r=-0.19, 95% CI: -0.33 to -0.05) and perceived inability of others to help (r=-0.15, 95% CI: -0.30 to -0.01) significantly predicted help-seeking
- **Limitations:** Small effect sizes, many assumed barriers (stigma) not significant

Article 7 (Rijk)
- **Objective:** Synthesize anorexia nervosa relapse predictors
- **Design:** Meta-analysis of 28 studies examining >150 predictors
- **Population:** 83–100% female, predominantly Caucasian, middle-high SES
- **Key Findings:** Post-treatment BMI (SMD=-0.35, 95% CI: -0.63 to -0.07) and pre-treatment depression (SMD=0.40, 95% CI: 0.21–0.59) predict relapse
- **Limitations:** Only 11% of studies rated good quality, significant methodological heterogeneity

Article 8 (Sala2023)
- **Objective:** Examine relapse predictors across all eating disorder types
- **Design:** Meta-analysis of 35 studies (N=2,577)
- **Population:** 98.1% female, 92.9% White
- **Key Findings:** Higher level of care (OR=1.81, 95% CI: 1.43–2.29), psychiatric comorbidity (OR=1.59, 95% CI: 1.20–2.10), and severe ED psychopathology (OR=1.57, 95% CI: 1.19–2.06) increase relapse risk
- **Subgroup Findings:** Diagnosis-specific effects; psychiatric comorbidity only predicts relapse in AN
- **Limitations:** Inconsistent relapse definitions, limited diversity

Article 9 (Solmi2024)
- **Objective:** Comprehensive outcomes atlas across eating disorder diagnoses
- **Design:** Meta-analysis of 415 studies (N=88,372)
- **Population:** 72.4% female, global sample
- **Key Findings:** Overall recovery 46% (95% CI: 44–49%), mortality 0.4% (95% CI: 0.2–0.7)
- **Subgroup Findings:** Children/adolescents have significantly better outcomes than adults
- **Limitations:** Heterogeneous outcome definitions, limited long-term follow-up data

SUMMARY FOR ARTICLE GROUP 6

Cluster Analysis Results: articles
Number of articles: 8
==
SYSTEMATIC UMBRELLA REVIEW CLUSTER ANALYSIS

1. Overall Cluster Label
COVID-19 Impact on Eating Disorders

Subcategories Identified:
- **Symptom Changes & Prevalence** (Articles 1, 2, 3, 7, 8)
- **Healthcare Utilization** (Article 4)
- **Cognitive Mechanisms** (Articles 5, 6)

2. Overall Findings Summary

Primary COVID-19 Impact Findings

Symptom Deterioration: Multiple studies consistently demonstrate significant worsening of eating disorder symptoms during COVID-19. Güzel2023 found increased ED prevalence (OR=1.30, 95% CI=1.05-1.54) and symptom severity for AN (d=0.29, 95% CI=0.12-0.46), BN (d=0.24, 95% CI=0.03-0.45), and BED (d=0.27, 95% CI=0.02-0.43). Haghshomar2022 reported 59.65% (95% CI=49.30%-69.60%) experienced deteriorated symptoms, while Khraisat2022 found 57% (95% CI=42%-71%) worsening. Sideli2021 confirmed 65% (95% CI=48%-81%) symptomatic deterioration.

Healthcare Utilization Surge: Madigan2025 demonstrated a substantial 54% increase in pediatric ED healthcare utilization (RR=1.54, 95% CI=1.38-1.71), with particularly pronounced effects in emergency departments (RR=1.70, 95% CI=1.48-1.97) and among adolescent girls (RR=1.48, 95% CI=1.28-1.71).

Psychological Comorbidity: Khraisat2022 found high rates of anxiety (64%, 95% CI=48%-79%) and depression (55%, 95% CI=21%-86%) among ED patients during COVID-19. Güzel2023 confirmed increased depression (d=0.21, 95% CI=0.07-0.35) and need for care (OR=1.74, 95% CI=1.48-2.00).

Cognitive Mechanism Findings

Repetitive Negative Thinking: Palmieri2021 established strong associations between RNT and eating disorders overall (d=0.85, 95% CI=0.72-0.98), with the strongest effects for AN (d=1.35, 95% CI=0.96-1.74), followed by BN (d=0.75, 95% CI=0.54-0.97) and BED (d=0.50, 95% CI=0.20-0.80).

Rumination Patterns: Rickerby2024 found large effect sizes for rumination differences between AN patients and controls (g=1.00, 95% CI=0.17-1.82), with moderate correlations between AN symptom severity and rumination (r=0.44, 95% CI=0.12-0.67).

Contrasting Findings

Longitudinal vs. Cross-sectional Discrepancy: vanReekum2023 found

no significant change in eating disorder symptoms using before-after comparisons (MD=0.44, 95% CI=-1.30 to 2.18), contrasting with cross-sectional studies showing high deterioration rates. This suggests perceived worsening may exceed objective changes.

3. Population Analysis

Gender Distribution
- **Articles 1, 2, 4, 7, 8** report gender data
- Consistently female-predominant: Güzel2023 (80% female), Madigan2025 (87% girls), Sideli2021 (85.7% female), vanReekum2023 (68% female)
- Madigan2025 found girls showed greater healthcare utilization increases than boys

Socioeconomic Status
- **No articles systematically report SES data** - representing a significant demographic gap across all studies

Race and Ethnicity
- **Articles 5, 6** provide limited race/ethnicity data
- Palmieri2021 notes some Caucasian and African-American participants but lacks systematic reporting
- Rickerby2024 reports predominantly Caucasian samples (only 9/99 studies had more non-Caucasian participants)
- **Major demographic bias:** Severe underrepresentation of diverse racial/ethnic groups

Notable Demographic Gaps
- **Geographic bias:** Predominantly Western countries (Articles 1, 3, 8)
- **Age concentration:** Focus on adolescents and young adults
- **Systematic exclusion** of diverse socioeconomic and racial/ethnic populations

4. Study Comparison

Similarities in Design and Methodology
- **Articles 1, 2, 3, 7** all employ systematic review and meta-analysis methodologies
- **Articles 1, 2, 3, 7, 8** use random-effects modeling for meta-analyses
- **Articles 1, 4** follow pre-registered protocols and PRISMA guidelines
- **Articles 5, 6** both examine cognitive mechanisms using comprehensive literature searches

Differences in Populations and Settings
- **Sample sizes vary dramatically:** vanReekum2023 (4,465), Güzel2023 (4,688,559), Khraisat2022 (3,056)
- **Geographic scope:** Madigan2025 covers 15 countries; others focus

on Western populations
- **Age ranges:** Madigan2025 (pediatric), others include broader age ranges
- **Clinical vs. general populations:** Articles 5, 6 include both; others focus on clinical samples

Convergent vs. Divergent Findings

Convergent Evidence:
- **Articles 1, 2, 3, 7** consistently show 50-65% of ED patients experienced symptom deterioration
- **Articles 1, 3** both demonstrate increased anxiety and depression comorbidity
- **Articles 5, 6** both establish rumination/RNT as significant factors in eating disorders

Divergent Evidence:
- **Article 8** shows no significant ED symptom changes using longitudinal design, contrasting with cross-sectional studies (Articles 2, 3, 7)
- **Article 1** reports some positive pandemic effects (family support), while others focus on negative impacts

Conclusion Similarities and Differences

Similar Conclusions:
- COVID-19 significantly impacted eating disorder populations
- Vulnerable populations (adolescents, females) were disproportionately affected
- Need for enhanced mental health services during crises

Different Conclusions:
- **Magnitude of impact:** Ranges from no change (Article 8) to substantial deterioration (Articles 1, 2, 7)
- **Recovery patterns:** Some suggest resilience (Article 8), others emphasize persistent difficulties
- **Intervention implications:** Vary from general support to specific cognitive interventions

5. Article-Specific Information

Article 1: Güzel2023
- **Objective:** Estimate magnitude of changes in ED pathology during COVID-19
- **Design:** Systematic review with frequentist and Bayesian meta-analyses
- **Population:** 4,688,559 subjects, 80% female, mean age 23 years
- **Key Findings:** ED prevalence OR=1.30 (1.05-1.54); AN severity d=0.29 (0.12-0.46)
- **Limitations:** High female representation, predominantly Western

samples

Article 2: Haghshomar2022
- **Objective:** Comprehensive overview of ED symptoms during COVID-19 confinement
- **Design:** Systematic review and meta-analysis of 13 studies
- **Population:** 7,848 participants, predominantly female
- **Key Findings:** 59.65% (49.30%-69.60%) deteriorated symptoms; 9.37% (3.92%-16.57%) improved
- **Limitations:** High heterogeneity (I^2=97.8%), publication bias detected

Article 3: Khraisat2022
- **Objective:** Evaluate pooled prevalence of psychological comorbidities in ED patients
- **Design:** Meta-analysis of 13 studies
- **Population:** 3,056 participants from multiple countries
- **Key Findings:** Anxiety 64% (48%-79%); depression 55% (21%-86%); ED worsening 57% (42%-71%)
- **Limitations:** Very high heterogeneity (95-99%), significant publication bias

Article 4: Madigan2025
- **Objective:** Quantify changes in pediatric ED healthcare utilization during COVID-19
- **Design:** Meta-analysis of 52 studies across 15 countries
- **Population:** >148,000 ED-related visits, 87% girls
- **Key Findings:** Overall RR=1.54 (1.38-1.71); girls RR=1.48 (1.28-1.71); adolescents RR=1.53 (1.29-1.81)
- **Limitations:** Limited SES and ethnicity data, potential healthcare access confounds

Article 5: Palmieri2021
- **Objective:** Examine relationship between repetitive negative thinking and eating disorders
- **Design:** Meta-analysis of 43 studies
- **Population:** 10,391 participants, predominantly female
- **Key Findings:** Overall d=0.85 (0.72-0.98); AN d=1.35 (0.96-1.74); BN d=0.75 (0.54-0.97)
- **Limitations:** Predominantly female samples, limited ethnic diversity

Article 6: Rickerby2024
- **Objective:** Compare rumination across depression, anxiety, and eating disorders
- **Design:** Meta-analysis of 99 studies
- **Population:** Predominantly female and Caucasian samples
- **Key Findings:** AN vs. controls g=1.00 (0.17-1.82); AN symptom correlation r=0.44 (0.12-0.67)
- **Limitations:** Limited diversity, publication bias in some

analyses

Article 7: Sideli2021
- **Objective:** Analyze pooled prevalence of ED and obesity symptoms during COVID-19
- **Design:** Meta-analysis of 26 studies
- **Population:** 6,117 total participants, 85.7% female
- **Key Findings:** 65% (48%-81%) symptomatic deterioration; 16% (07%-24%) improvement
- **Limitations:** Predominantly cross-sectional studies, potential recall bias

Article 8: vanReekum2023
- **Objective:** Examine psychiatric symptom changes in preexisting mental health conditions
- **Design:** Meta-analysis of before-after studies
- **Population:** 4,465 participants, 68% female, mean age 43
- **Key Findings:** ED symptoms showed no significant change (MD=0.44, -1.30 to 2.18)
- **Limitations:** Very low certainty evidence, high heterogeneity, limited ED-specific studies

SUMMARY FOR ARTICLE GROUP 7

Cluster Analysis Results: articles
Number of articles: 6
==

SYSTEMATIC UMBRELLA REVIEW CLUSTER SYNTHESIS

1. Overall Cluster Label
Pharmacological Interventions for Anorexia Nervosa

Subcategories Identified:
- Traditional psychotropic medications (antidepressants, antipsychotics)
- Neuropeptide interventions (intranasal oxytocin)
- Biomarker studies (peripheral oxytocin levels)
- Treatment tolerability and dropout patterns

2. Overall Findings Summary

Primary Efficacy Findings:
Olanzapine for Weight Gain (Han2022, Cassioli2020):
- Adults: Significant BMI increase MD = 0.67 kg/m^2 (95% CI 0.15-1.18, p = 0.01) - Han2022
- Adolescents: Non-significant BMI increase of 0.66 kg/m^2 (95% CI -0.36 to 1.67, p = 0.21) - Han2022
- Cassioli2020 found no significant difference between olanzapine and placebo across 5 studies

Intranasal Oxytocin Effects:
- Social cognition in AN: No significant effects on attentional bias SMD = 0.20 (95% CI -0.16 to 0.57) or emotion recognition SMD = -0.01 (95% CI -0.27 to 0.26) - Hasselbalch2020
- Food intake in AN: SMD = 0.17 (95% CI -0.32 to 0.66) - no effect - Chen2021
- Food intake in non-psychiatric subjects: SMD = -0.66 (95% CI -1.18 to -0.14) - significant reduction - Chen2021

Traditional Psychotropic Medications (Cassioli2020):
- Antidepressants vs placebo for depression: SMD = -0.27 (95% CI -0.73 to 0.20) - non-significant
- No significant effects for any psychotropic medication on AN psychopathology, depression, or anxiety

Peripheral Oxytocin Biomarkers (Ferreira2022):
- Restricting AN: SMD = -1.43 (95% CI -2.05 to -0.81) - significantly lower levels
- Binge-eating/purging AN: SMD = -1.18 (95% CI -2.19 to -0.16) - significantly lower levels

Treatment Tolerability:
Dropout Rates (Kan2020): 28% (95% CI: 19-38%) for second-generation antipsychotics, with personal choices (43.5%) rather than metabolic effects being the primary reason for discontinuation.

Most Scientifically Relevant Finding:
The convergent evidence across multiple studies (Cassioli2020, Chen2021, Hasselbalch2020) demonstrating limited efficacy of pharmacological interventions in AN, contrasted with the significant oxytocin dysregulation found in biomarker studies (Ferreira2022).

Most Clinically Relevant Finding:
Olanzapine shows modest but significant weight gain benefits in adults (Han2022), with acceptable dropout rates (Kan2020), making it the only intervention with consistent positive evidence for clinical use.

3. Population Analysis

Gender Distribution:
- **Articles reporting gender data**: Hasselbalch2020 (females only), Kan2020 (88.9-100% female), Chen2021 (mixed - 5 trials males only, 6 trials females only)
- **Articles not reporting gender**: Cassioli2020, Han2022, Ferreira2022 (mixed samples noted but not systematically reported)

Socioeconomic Status:
- **No articles reported SES data**

Race and Ethnicity:
- **No articles systematically reported race/ethnicity data**

Notable Demographic Gaps:
- Severe underrepresentation of male participants across all studies
- Complete absence of SES and race/ethnicity data represents major demographic bias
- Geographic bias noted in Kan2020 (67% of studies from USA/Canada)

4. Study Comparison

Similarities in Design and Methodology:
- **All articles** used systematic review and meta-analysis methodology following PRISMA guidelines
- **Cassioli2020, Chen2021, Han2022, Hasselbalch2020** focused on RCT evidence
- **All articles** acknowledged small sample sizes and methodological limitations in primary literature

Population and Setting Differences:
- **AN-specific populations**: Cassioli2020, Han2022, Hasselbalch2020, Kan2020
- **Broader psychiatric populations**: Chen2021, Ferreira2022
- **Healthy control inclusion**: Chen2021, Hasselbalch2020, Ferreira2022
- **Age range differences**: Han2022 (adults and adolescents), others primarily adults

Convergent Findings:
- **Limited pharmacological efficacy**: Cassioli2020, Chen2021, Hasselbalch2020 all found minimal to no effects of interventions in AN populations
- **Methodological concerns**: All studies highlighted small sample sizes, heterogeneity, and methodological limitations in primary research
- **Need for psychological treatments**: Cassioli2020 and Chen2021 both concluded psychological treatments remain first-line

Divergent Findings:
- **Olanzapine efficacy**: Han2022 found significant effects vs. Cassioli2020 found non-significant effects
- **Oxytocin effects**: Chen2021 found effects in healthy populations while Hasselbalch2020 found no effects in AN
- **Biomarker vs. intervention studies**: Ferreira2022 found significant oxytocin dysregulation while intervention studies found limited therapeutic effects

Conclusion Similarities:
All studies concluded that current evidence for pharmacological interventions in AN is limited, with calls for larger, better-designed

studies.

Conclusion Differences:
Han2022 and Kan2020 provided more optimistic assessments of olanzapine's clinical utility, while Cassioli2020, Chen2021, and Hasselbalch2020 emphasized the lack of evidence for pharmacological approaches.

5. Article-Specific Information

Cassioli2020
- **Objective**: Evaluate psychopharmacological treatments for acute-phase AN
- **Design**: Meta-analysis of 19 RCTs, 8 in quantitative analysis (n=99 active treatment)
- **Population**: Acute-phase AN patients
- **Primary Findings**: No significant effects for any psychotropic medication on weight recovery or psychiatric symptoms
- **Subgroup Analyses**: Antidepressants vs placebo SMD = -0.27 (95% CI -0.73 to 0.20)
- **Limitations**: Small sample sizes, heterogeneous interventions, short follow-up periods

Chen2021
- **Objective**: Examine intranasal oxytocin effects on food intake and craving
- **Design**: Meta-analysis of 12 trials (n=423 total participants)
- **Population**: Non-psychiatric subjects and psychiatric patients (AN, BN, BED, schizophrenia)
- **Primary Findings**: Significant reduction in food intake in non-psychiatric subjects SMD = -0.66 (95% CI -1.18 to -0.14), no effects in psychiatric populations
- **Subgroup Analyses**: No significant effects in AN, BN/BED, or schizophrenia subgroups
- **Limitations**: Single-dose studies, short-term effects, small sample sizes

Ferreira2022
- **Objective**: Assess peripheral oxytocin levels across psychiatric disorders
- **Design**: Meta-analysis of 55 studies across 9 psychiatric conditions
- **Population**: Mixed psychiatric patients and healthy controls
- **Primary Findings**: Lower OXT in restricting AN SMD = -1.43 (95% CI -2.05 to -0.81), binge-eating/purging AN SMD = -1.18 (95% CI -2.19 to -0.16)
- **Subgroup Analyses**: Disorder-specific patterns with some showing deficits, others elevations
- **Limitations**: High heterogeneity, methodological variability in oxytocin measurement

Han2022
- **Objective**: Evaluate olanzapine effectiveness for weight gain in AN
- **Design**: Meta-analysis of 7 articles (n=304 AN patients)
- **Population**: Adult and adolescent AN patients
- **Primary Findings**: Adults - significant BMI increase MD = 0.67 kg/m^2 (95% CI 0.15-1.18); Adolescents - non-significant
- **Subgroup Analyses**: Age-specific effects with adults showing benefit, adolescents not
- **Limitations**: Limited number of studies, heterogeneous designs, short follow-up

Hasselbalch2020
- **Objective**: Examine intranasal oxytocin effects on social cognition in AN
- **Design**: Meta-analysis of 6 publications from 4 trials (n=224)
- **Population**: Female AN patients and healthy controls
- **Primary Findings**: No significant effects on attentional bias SMD = 0.20 (95% CI -0.16 to 0.57) or emotion recognition SMD = -0.01 (95% CI -0.27 to 0.26)
- **Subgroup Analyses**: No effects in AN or healthy control groups separately
- **Limitations**: Small sample sizes, potential dosing/timing issues, limited cognitive measures

Kan2020
- **Objective**: Examine dropout rates and metabolic effects of second-generation antipsychotics in AN
- **Design**: Meta-analysis of 21 studies (10 in dropout analysis)
- **Population**: AN patients (88.9-100% female)
- **Primary Findings**: Pooled dropout rate 28% (95% CI: 19-38%)
- **Subgroup Analyses**: Dropout reasons - personal choices (43.5%), study factors (25.8%), poor response (16.1%), adverse events (12.9%)
- **Limitations**: Heterogeneous study designs, limited geographic diversity, retrospective data for dropout reasons

SUMMARY FOR ARTICLE GROUP 8

Cluster Analysis Results: articles
Number of articles: 3
==

SYNTHESIZED CLUSTER ANALYSIS

1. Overall Cluster Label: **ARFID Clinical Characteristics and Prevalence**

Subcategories: This cluster contains three distinct but complementary subcategories:

- Physical health complications and anthropometric characteristics
- Population prevalence estimates and diagnostic considerations
- Comorbidity patterns with autism spectrum disorder

2. Overall Findings Summary

This cluster provides comprehensive evidence on ARFID as a clinically significant eating disorder with distinct characteristics from anorexia nervosa. The most scientifically relevant finding is the establishment of ARFID prevalence at **4.51% (95% CI: 0.7-10.68%)** using quality-effects meta-analysis (Nicholls-Clow2024), representing a 15-fold higher rate than previously estimated. The most clinically relevant finding is that ARFID presents with significant physical health complications across the entire weight spectrum, with **BMI z-scores significantly lower than healthy controls (SMD = -0.88, 95% CI: -1.40 to -0.47)** but **equivalent to anorexia nervosa (SMD = -0.00, 95% CI: -0.31, 0.31)** (James2024).

Key Clinical Findings:
- **Cardiovascular differences:** ARFID patients show significantly higher heart rates compared to AN patients (**WMD = 12.82 bpm, 95% CI: 7.58-18.12**) despite similar weight status (James2024)
- **Autism comorbidity:** Autism prevalence in ARFID populations is **16.27% (95% CI: 8.64%-28.53%)**, over 15 times higher than general population rates (Sader2025)
- **Population distribution:** Clinical samples show higher ARFID prevalence (**18.61%**) compared to non-clinical samples (**4.46%**) using random-effects analysis (Nicholls-Clow2024)

Subgroup and Moderation Analyses:
- Gender effects show males having higher ARFID prevalence than females across studies (Nicholls-Clow2024)
- Age differences indicate higher prevalence in children versus adults in random-effects models (**13.55% vs. 8.8%**) but similar rates in quality-effects models (Nicholls-Clow2024)
- Study quality significantly moderates prevalence estimates, with quality-effects analysis yielding substantially lower and more conservative estimates than traditional random-effects approaches (Nicholls-Clow2024)

3. Population Analysis

Gender Distribution:
- **James2024:** Gender data not specifically reported for overall sample demographics
- **Nicholls-Clow2024:** Mixed samples with male (k=7) and female (k=8) studies; only one study included non-binary participants
- **Sader2025:** 50.10% male in ARFID samples; 58.12% male in autism samples

Socioeconomic Status:
- **All three articles:** No SES data reported, representing a significant demographic gap

Race and Ethnicity:
- **James2024:** Not reported
- **Nicholls-Clow2024:** Not reported (though studies from 12 countries included)
- **Sader2025:** Predominantly Caucasian/white (61.64%); 11/21 studies did not specify ethnic distribution

Notable Demographic Gaps:
- Systematic lack of SES reporting across all studies
- Limited racial/ethnic diversity with Western, Caucasian populations overrepresented
- Minimal non-binary gender representation
- Geographic concentration in Western countries limiting generalizability

4. Study Comparison

Similarities in Design and Methodology:
- All three articles employed systematic review and meta-analysis methodologies
- All used random-effects models due to high heterogeneity (I^2 > 90% across studies)
- All followed established systematic review guidelines (PRISMA, pre-registered protocols)
- All focused on clinical populations with validated diagnostic criteria

Differences in Populations and Settings:
- **James2024:** Exclusively pediatric populations (≤18 years) from clinical samples
- **Nicholls-Clow2024:** Mixed child and adult populations from both clinical and non-clinical settings
- **Sader2025:** Mixed age populations but predominantly child/adolescent samples from eating disorder and autism clinical services

Convergent Findings:
- All studies demonstrate ARFID as a clinically significant condition requiring specialized attention
- High heterogeneity across studies reflects diverse clinical presentations (supported by all three articles)
- Male predominance or equal gender distribution differs from other eating disorders (Nicholls-Clow2024, Sader2025)

Divergent Findings:
- **Prevalence estimates:** Dramatic differences between random-effects (11.14%) and quality-effects (4.51%) approaches highlight

methodological sensitivity (Nicholls-Clow2024)
- **Weight status:** James2024 emphasizes complications across weight spectrum, while traditional views focus on underweight presentations

Conclusion Similarities:
All three studies conclude that ARFID requires specialized clinical protocols, comprehensive assessment approaches, and recognition as a distinct eating disorder category with unique risk profiles.

Conclusion Differences:
- **James2024:** Emphasizes physical health monitoring regardless of weight status
- **Nicholls-Clow2024:** Focuses on diagnostic standardization and service planning
- **Sader2025:** Highlights need for cross-condition screening protocols

5. Article-Specific Information

James2024
- **Research Question:** What are the physical health complications in children and young people with ARFID?
- **Design:** Systematic review and meta-analysis of 131 studies (n=8,047 initial results)
- **Population:** Children ≤18 years from clinical samples
- **Primary Findings:** BMI z-scores significantly lower than healthy controls (SMD = -0.88, 95% CI: -1.40 to -0.47) but equivalent to AN; higher heart rates than AN (WMD = 12.82 bpm, 95% CI: 7.58-18.12)
- **Subgroup Analyses:** Cardiovascular differences between ARFID and AN despite similar anthropometric profiles
- **Limitations:** High heterogeneity (I^2 = 68.7-78.5%), predominantly cross-sectional studies, limited longitudinal data

Nicholls-Clow2024
- **Research Question:** What is the prevalence of ARFID across clinical and non-clinical populations?
- **Design:** Systematic review and meta-analysis of 26 studies (n=122,861)
- **Population:** Mixed child and adult populations across 12 countries
- **Primary Findings:** Quality-effects prevalence 4.51% (95% CI: 0.7-10.68%) vs. random-effects 11.14% (95% CI: 8.16-14.5%)
- **Subgroup Analyses:** Clinical vs. non-clinical (18.61% vs. 4.46%); male vs. female predominance; children vs. adults (13.55% vs. 8.8% random-effects)
- **Limitations:** High heterogeneity (I^2 = 99.6%), publication bias, limited standardized diagnostic measures

Sader2025
- **Research Question:** What is the co-occurrence rate between autism

and ARFID?
- **Design:** Systematic review and meta-analysis of 21 studies (n=7,442)
- **Population:** Mixed ARFID (n=1,708) and autism (n=5,734) clinical samples
- **Primary Findings:** Autism prevalence in ARFID: 16.27% (95% CI: 8.64%-28.53%); ARFID prevalence in autism: 11.41% (95% CI: 2.89%-35.76%)
- **Subgroup Analyses:** Gender and ethnicity as significant sources of heterogeneity
- **Limitations:** High heterogeneity (I^2 > 93%), publication bias (p = 0.0133), limited population-based studies, geographic concentration in Western countries

SUMMARY FOR ARTICLE GROUP 9

Cluster Analysis Results: articles
Number of articles: 23
==
SYSTEMATIC UMBRELLA REVIEW CLUSTER SYNTHESIS

1. Overall Cluster Label
Eating Disorders Treatment Interventions Research

Subcategories Identified:
- Pharmacological interventions (9 articles)
- Psychotherapy and behavioral interventions (7 articles)
- Neurostimulation therapies (4 articles)
- Exercise and mind-body interventions (3 articles)

2. Overall Findings Summary

Most Scientifically Relevant Findings

Pharmacological Interventions:
- **Lisdexamfetamine for BED** showed the strongest evidence with large effect sizes (SMD = 0.93, 95% CI: 0.74-1.12) for binge eating reduction (Schneider2021)
- **Olanzapine for AN** demonstrated modest but significant BMI increases (Hedges' g = 0.283, 95% CI: 0.051-0.515) (Fornaro2023)
- **Fluoxetine for BN** showed consistent benefits for binge episode reduction (Hedges' g = 0.203, 95% CI: 0.007-0.399) and mood symptoms (Fornaro2023)
- **GLP-1 agonists for BED** demonstrated significant weight loss (WMD = -3.81 kg, 95% CI: -5.14 to -2.49) and binge eating reduction (WMD = -8.14 points, 95% CI: -13.13 to -3.15) (Radkhah2025)

Neurostimulation Interventions:
- **rTMS for eating disorders** showed moderate effects on craving reduction (Hedge's g = -0.228, 95% CI: -0.355 to -0.102) with

strongest effects for stimulant addictions and behavioral addictions (Gay2022)
- **TMS for anorexia nervosa** demonstrated significant BMI improvements (SMD: -0.025, 95% CI: -0.0505 to -0.005) and eating disorder symptom reduction (SMD: 0.634, 95% CI: 0.349-0.919) (Bahadori2025)
- **Deep brain stimulation for treatment-refractory AN** showed large effects for weight restoration (Hedges' g = 1.13, 95% CI: 0.80-1.46) (Karaszewska2022)

Psychotherapy Interventions:
- **CBT for AN** demonstrated large effect sizes for weight gain (g = 0.87, 95% CI: 0.67-1.08) and medium-large effects for eating disorder symptom reduction (g = -0.74, 95% CI: -0.93 to -0.54) (Duggan2025)
- **Mindfulness-based interventions for binge eating** showed small but significant effects (g = -0.39, 95% CI: -0.68 to -0.11) that did not maintain at follow-up (Grohmann2021)

Most Clinically Relevant Findings

Treatment Accessibility and Effectiveness:
- CBT vs. pharmacotherapy comparisons revealed limited evidence supporting CBT's presumed superiority, with some medications showing comparable efficacy (Samara2024)
- Pharmacotherapy for bulimia nervosa showed significant benefits challenging beliefs about its ineffectiveness (binge frequency SMD -0.47, 95% CI: -0.66 to -0.28) (Argyrou2023)

Safety and Tolerability:
- Exercise interventions in AN were found to be safe when properly supervised, with no compromise to weight gain (QuilesMarcos2021)
- Most neurostimulation interventions showed minimal adverse effects with good tolerability profiles across studies

Subgroup and Moderation Analyses:
- **Dose-response relationships** were identified for several interventions: naltrexone dosage moderated binge eating outcomes (Fang2025), and TMS sessions >20 minutes showed superior effects (Bahadori2025)
- **Treatment duration effects**: Interventions >12 months showed superior outcomes for AN (BMI: SMD = 0.35, 95% CI: 0.07-0.64) (Gan2022)
- **Pre-treatment BMI** was the only significant moderator for CBT effectiveness in AN, with lower starting BMI predicting greater weight gain (Duggan2025)

3. Population Analysis

Gender Distribution
Articles with gender data (18/23):

- Predominantly female populations across all eating disorder studies (typically 85-100% female)
- Argyrou2023: >98% women
- Bahadori2025, Cavicchioli2022, Fornaro2023: Mixed but predominantly female
- Duggan2025: 92-100% female across studies
- Fang2025: 77.4% female
- Gan2022: 94.2% female (1,385 females, 85 males)
- Grohmann2021: 88.56% female
- Karaszewska2022: 98.2% female (55 females, 1 male)
- Pennesi2024: 93.7% female
- QuilesMarcos2021: 81.8-100% female
- Sioziou2024: 84.9% female

Socioeconomic Status
No articles reported comprehensive SES data - representing a significant demographic gap across the entire cluster.

Race and Ethnicity
Minimal reporting across all articles:
- Only Duggan2025 noted "mostly white participants (85-100%)" with rare ethnicity reporting
- Grohmann2021 acknowledged "underrepresentation of ethnic minority backgrounds"
- All other articles (21/23) provided no race/ethnicity data

Notable Demographic Gaps
- **Severe underrepresentation of male participants** across all eating disorder research
- **Complete absence of SES reporting** limiting understanding of treatment accessibility
- **Minimal racial/ethnic diversity** in study populations
- **Geographic bias** toward Western, developed countries

4. Study Comparison

Similarities in Study Designs
Methodological Convergence (Articles sharing features):
- **Systematic review/meta-analysis approach:** 20/23 articles used this design
- **PRISMA guidelines adherence:** Bilbao2022, Bravo2024, Duggan2025, Fang2025, Fornaro2023, Martinez-Calderon2025, Moawad2024, Nourredine2020, Samara2024
- **Random-effects meta-analysis models:** Used consistently across quantitative syntheses
- **Multiple database searches:** Standard across all systematic reviews
- **Risk of bias assessment:** Cochrane tools used in Duggan2025, Grohmann2021, Martinez-Calderon2025, QuilesMarcos2021

Differences in Populations and Settings
Population Variations:
- **Age ranges:** Adolescent-focused (Gan2022), adult-focused (most studies), or mixed populations
- **Eating disorder subtypes:** AN-specific (Bahadori2025, Duggan2025, Gan2022, Karaszewska2022, QuilesMarcos2021), BED-specific (Fang2025, Moawad2024, Nourredine2020, Radkhah2025, Schneider2021, Sioziou2024), or mixed ED populations
- **Treatment settings:** Outpatient focus (Duggan2025), inpatient considerations (Karaszewska2022), or mixed settings

Convergent vs. Divergent Findings

Convergent Findings:
- **Small to moderate effect sizes** consistently reported across intervention types
- **Better outcomes with longer treatment duration** (Duggan2025, Gan2022, Gay2022)
- **Dose-response relationships** identified across multiple interventions (Bahadori2025, Fang2025, Gay2022)
- **Safety and tolerability** generally acceptable across most interventions

Divergent Findings:
- **CBT superiority questioned:** Samara2024 found limited evidence for CBT superiority over pharmacotherapy, contradicting traditional treatment hierarchies
- **Maintenance of effects:** Grohmann2021 found benefits didn't persist at follow-up for mindfulness interventions, while other studies showed sustained effects
- **Exercise safety:** Martinez-Calderon2025 found limited evidence for exercise benefits, while QuilesMarcos2021 supported supervised exercise as safe and potentially beneficial

Similarities and Differences in Conclusions

Similar Conclusions:
- **Need for larger, longer-term studies** emphasized across multiple articles
- **Importance of individualized treatment approaches** recognized
- **Combination therapies** may be superior to single interventions
- **Accessibility barriers** to evidence-based treatments acknowledged

Differing Conclusions:
- **Treatment hierarchy:** Traditional CBT-first approaches questioned by some studies (Samara2024) while supported by others (Duggan2025)
- **Intervention intensity:** Some studies favored intensive approaches while others found minimal differences
- **Population-specific recommendations:** Varying conclusions about optimal treatments for different ED subtypes

5. Article-Specific Information

Pharmacological Interventions

Argyrou2023 - Bulimia nervosa pharmacotherapy meta-analysis
- *Objective:* Evaluate all pharmacotherapy forms for BN treatment
- *Design:* Meta-analysis of 52 RCTs (N=3,313)
- *Population:* >98% women, mean age 26.43±5.25 years
- *Key findings:* Significant binge frequency reduction (SMD -0.47, 95% CI: -0.66 to -0.28), treatment response improvement (RR 1.93, 95% CI: 1.50-2.50)
- *Limitations:* Limited remission data, high heterogeneity, older studies with potential bias

Fang2025 - Opioid antagonists for binge eating
- *Objective:* Examine opioid antagonist efficacy in binge eating behavior
- *Design:* Meta-analysis of 8 RCTs (N=387)
- *Population:* 77.4% female, mean age 42.6 years
- *Key findings:* Significant binge frequency reduction (SMD = -0.624, 95% CI: -1.181 to -0.067), large weight loss effect (SMD = -0.981, 95% CI: -1.657 to -0.305)
- *Limitations:* Small number of studies, high heterogeneity, short treatment duration

Fornaro2023 - Comprehensive eating disorders pharmacotherapy
- *Objective:* Assess psychopharmacological treatments across AN, BN, and BED
- *Design:* Meta-analysis of 62 studies (22 in quantitative analysis)
- *Population:* Predominantly female across all disorders
- *Key findings:* Disorder-specific efficacy patterns - olanzapine for AN (Hedges' g = 0.283), fluoxetine for BN, lisdexamfetamine for BED
- *Limitations:* Small sample sizes, short trial durations, limited evidence base

Moawad2024 - Naltrexone/bupropion combination for BED
- *Objective:* Evaluate naltrexone/bupropion efficacy in BED and obesity
- *Design:* Meta-analysis of 6 studies (5 RCTs, 1 open-label)
- *Population:* Predominantly female
- *Key findings:* Significant weight reduction (MD: -8.52, 95% CI: -10.01 to -6.94), BES reduction (MD: -7.66, 95% CI: -14.36 to 0.96)
- *Limitations:* Mixed study designs, limited long-term data

Nourredine2020 - Topiramate for BED
- *Objective:* Assess topiramate efficacy and safety in BED
- *Design:* Meta-analysis of 3 RCTs (N=528)
- *Population:* One study 95% female
- *Key findings:* Significant binge reduction (MD = -1.31, 95% CI:

-2.58 to -0.03), weight loss (MD = -4.91 kg, 95% CI: -6.42 to -3.41)
- *Limitations:* High heterogeneity (I^2 = 94%), safety concerns with cognitive impairment

Radkhah2025 - GLP-1 agonists for eating disorders
- *Objective:* Evaluate GLP-1 receptor agonists in eating disorders, focusing on BED
- *Design:* Meta-analysis of 5 studies (N=182)
- *Population:* Mixed gender (46.1-89.4% female)
- *Key findings:* Significant weight loss (WMD = -3.81 kg, 95% CI: -5.14 to -2.49), binge eating reduction (WMD = -8.14, 95% CI: -13.13 to -3.15)
- *Limitations:* Small sample size, limited long-term data, emerging treatment area

Schneider2021 - Lisdexamfetamine for BED
- *Objective:* Examine LDX efficacy combining preclinical and clinical evidence
- *Design:* Meta-analysis of 14 clinical and 7 preclinical studies
- *Population:* Clinical: 81.5-92% female; Preclinical: mixed
- *Key findings:* Large effect on binge-eating symptoms (SMD = 0.93, 95% CI: 0.74-1.12), consistent weight reduction
- *Limitations:* Limited mechanistic understanding, need for BMI considerations

Sioziou2024 - Antidepressants for BED
- *Objective:* Examine antidepressant efficacy vs. placebo in BED
- *Design:* Meta-analysis of 16 RCTs (N=984)
- *Population:* 84.9% female
- *Key findings:* Modest remission improvement (RR: 1.39, 95% CI: 1.04-1.86), binge episode reduction (SMD: -0.29, 95% CI: -0.51 to -0.06)
- *Limitations:* Small effect sizes, bias concerns with sponsored studies

Neurostimulation Interventions

Bahadori2025 - TMS for anorexia nervosa
- *Objective:* Evaluate TMS efficacy in AN treatment
- *Design:* Meta-analysis of 17 studies (9 in meta-analysis, N=129)
- *Population:* Mean age 32.41±7.27 years, disease duration 14.57±6.66 years
- *Key findings:* Significant BMI increase (SMD: -0.025, 95% CI: -0.0505 to -0.005), EDE-Q reduction (SMD: 0.634, 95% CI: 0.349-0.919)
- *Limitations:* Mixed study designs, limited long-term follow-up, small sample sizes

Cavicchioli2022 - rTMS for eating disorders and obesity
- *Objective:* Assess rTMS efficacy across eating disorders and obesity spectrum

- *Design:* Meta-analysis of 15 studies (N=402)
- *Population:* Mixed gender
- *Key findings:* Large BMI improvement in obesity (d = -0.85, 95% CI: -1.14 to -0.56), null effects in AN, moderate negative affectivity improvement (d = -0.47, 95% CI: -0.68 to -0.26)
- *Limitations:* High heterogeneity, small studies, heterogeneous protocols

Gay2022 - rTMS for addiction craving
- *Objective:* Evaluate rTMS efficacy for craving across addiction types including eating disorders
- *Design:* Meta-analysis of 34 studies (42 units of analysis)
- *Population:* Mixed across addiction types
- *Key findings:* Small significant effect (Hedge's g = -0.228, 95% CI: -0.355 to -0.102), stronger effects for stimulant and behavioral addictions
- *Limitations:* High heterogeneity, variable stimulation parameters

Karaszewska2022 - Deep brain stimulation for treatment-refractory AN
- *Objective:* Assess DBS efficacy in severe, treatment-resistant AN
- *Design:* Meta-analysis of 4 studies (N=56)
- *Population:* 55 female, 1 male; mean age 29.8 years, illness duration 12.8 years
- *Key findings:* Large BMI effect (Hedges' g = 1.13, 95% CI: 0.80-1.46), significant psychiatric symptom improvement
- *Limitations:* No control groups, small sample size, moderate-to-serious risk of bias

Psychotherapy and Behavioral Interventions

Duggan2025 - CBT for anorexia nervosa
- *Objective:* Evaluate CBT effectiveness in outpatient AN treatment
- *Design:* Meta-analysis of 26 studies (9 case series, 8 cohort, 7 RCTs, others)
- *Population:* 92-100% female, mostly white participants
- *Key findings:* Large weight gain effect (g = 0.87, 95% CI: 0.67-1.08), medium-large symptom reduction (g = -0.74, 95% CI: -0.93 to -0.54)
- *Subgroup findings:* Pre-treatment BMI was only significant moderator
- *Limitations:* English-language bias, single reviewer limitations, Western researcher predominance

Gan2022 - Non-pharmacological interventions for AN
- *Objective:* Synthesize evidence on face-to-face non-pharmacological interventions for AN
- *Design:* Meta-analysis of 19 RCTs (N=1,470)
- *Population:* 94.2% female, ages 14-43 years
- *Key findings:* Overall non-significant effects, but BFST vs. ego-

oriented therapy showed large BMI effects (SMD = 0.84, 95% CI: 0.00–1.69)
- *Subgroup findings:* Combined interventions and >12-month duration more effective
- *Limitations:* Small sample sizes, limited geographic representation, active control comparisons

Grohmann2021 - Mindfulness-based interventions for binge eating
- *Objective:* Assess MBI efficacy for binge eating over two decades
- *Design:* Meta-analysis of 21 studies (11 RCTs, 10 pre-post)
- *Population:* 88.56% female
- *Key findings:* Small end-of-trial effect (g = -0.39, 95% CI: -0.68 to -0.11), non-significant at follow-up (g = -0.06, 95% CI: -0.31 to 0.20)
- *Subgroup findings:* Stronger effects with wait-list vs. active controls, clinical vs. non-clinical populations
- *Limitations:* Small sample sizes, high dropout rates, limited diversity

Pennesi2024 - Acute augmentations to psychological therapies
- *Objective:* Examine acute augmentation effectiveness in eating disorder treatment
- *Design:* Meta-analysis of 29 studies (17 RCTs in meta-analysis)
- *Population:* 93.7% female (N=1,831 total, 1,162 in meta-analysis)
- *Key findings:* Small significant effect favoring augmentation (Hedges' g = 0.14, 95% CI: 0.02–0.26)
- *Limitations:* Wide variety of augmentation types, small subgroups

Samara2024 - CBT vs. pharmacotherapy comparison
- *Objective:* Compare CBT and pharmacotherapy effectiveness for binge spectrum disorders
- *Design:* Meta-analysis of 11 RCTs (N=531)
- *Population:* Mean age 31.19 years, 60.07% BN, 39.93% BED
- *Key findings:* Limited evidence for CBT superiority; CBT superior to fluoxetine for remission (RR=3.24, 95% CI: 1.46–7.18) but not to sibutramine or methylphenidate
- *Limitations:* Very small sample sizes, single studies per comparison, older medications only

Exercise and Mind-Body Interventions

Bravo2024 - Movement awareness therapies for eating disorders
- *Objective:* Assess effectiveness of body and movement awareness interventions
- *Design:* Meta-analysis of 11 RCTs (N=437)
- *Population:* 90.2–100% female, ages 14–72 years
- *Key findings:* Modest EDE score improvement (WMD = -0.30, 95% CI: -0.57 to -0.02), non-significant symptom severity effects
- *Limitations:* No high-quality RCTs, methodological limitations, small samples

Martinez-Calderon2025 - Exercise interventions for eating disorders
- *Objective:* Evaluate exercise and mind-body exercise effectiveness in feeding/eating disorders
- *Design:* Meta-analysis of 12 RCTs (N=690)
- *Population:* >50% female
- *Key findings:* Small effect for harmful exercise habits (SMD 0.22, 95% CI: 0.14-0.30), no significant effects for other outcomes
- *Limitations:* Low to very low GRADE evidence quality, poor intervention reporting

QuilesMarcos2021 - Exercise-based interventions for AN
- *Objective:* Examine exercise intervention outcomes in AN patients
- *Design:* Meta-analysis of 10 studies (6 RCTs, 3 quasi-randomized, 1 single-group)
- *Population:* 81.8-100% female, predominantly Caucasian
- *Key findings:* No significant BMI differences (difference = -0.53, 95% CI: -1.52 to 0.46), strength improvements with resistance programs
- *Limitations:* Methodological limitations in primary studies, heterogeneous interventions

Specialized Reviews

Bilbao2022 - Medical cannabinoids across conditions
- *Objective:* Pharmacology-based systematic review of four medical cannabinoids across 23 conditions
- *Design:* Meta-analysis of 152 RCTs (N=12,123)
- *Population:* Not specified for eating disorders subset
- *Key findings:* CBD showed highest quality evidence for epilepsy (SMD -0.50, 95% CI: -0.62 to -0.38), distinct therapeutic profiles by cannabinoid type
- *Limitations:* Most evidence low-to-moderate quality, limited eating disorder specific data

Xu2022 - Non-invasive brain stimulation for eating disorders
- *Objective:* Examine NIBS (rTMS and tDCS) effects on eating disorders
- *Design:* Meta-analysis of 15 studies (9 RCTs with N=266, 6 one-arm with N=59)
- *Population:* Not specified
- *Key findings:* Moderate positive effects (g = -0.48), dose-response relationship identified
- *Limitations:* Small sample sizes, limited long-term data

Yu2023 - Neuroimaging meta-analysis of anticipatory responses
- *Objective:* Examine altered anticipatory brain responses in eating disorders
- *Design:* ALE meta-analysis of 21 neuroimaging studies
- *Population:* Predominantly female

- *Key findings:* Hyperactivation in right putamen, hypoactivation in left inferior parietal lobule
- *Limitations:* Small number of studies, predominance of female participants

SUMMARY FOR ARTICLE GROUP 10

Cluster Analysis Results: articles
Number of articles: 19
==
SYSTEMATIC UMBRELLA REVIEW CLUSTER SYNTHESIS

1. Overall Cluster Label
Eating Disorders Biomarkers and Pathophysiology

This cluster contains two distinct subcategories:
- **Biological markers and mechanisms** (16 articles): Studies examining biomarkers, hormones, genetics, cardiac function, and pathophysiological mechanisms
- **Treatment interventions and prevalence** (3 articles): Studies on therapeutic approaches and epidemiological data

2. Overall Findings Summary

Primary Biological Findings

Hormonal Dysregulation:
- **Leptin**: Consistently decreased across multiple studies (Karageorgiou2020: SMD -3.03 to -3.84; Wu2024: g=-1.62, 95% CI -1.89 to -1.36)
- **Ghrelin**: Significantly elevated in all forms (Seidel2021: total ghrelin SMD=2.51, 95% CI 1.68-3.33; acyl ghrelin SMD=2.02, 95% CI 1.14-2.89)
- **Cortisol**: Elevated across measurement methods (Thavaraputta2023: morning serum MD=4.58 μg/dL, 95% CI 3.22-5.94; 24-hour urine MD=30.06 μg/day, 95% CI 20.61-39.51)
- **Adiponectin**: Elevated in AN (Karageorgiou2020: SMD 1.74, 95% CI 1.01-2.47; Tural2022: Hedges' g=0.765, 95% CI 0.599-0.932)

Growth Factors and Neurotrophins:
- **BDNF**: Decreased in eating disorders (Keeler2022: SMD=-0.51, 95% CI -0.82 to -0.21; Shobeiri2022: SMD=-0.49, 95% CI -0.89 to -0.08)
- **IGF-1**: Significantly reduced (Keeler2022: SMD=-1.57, 95% CI -1.84 to -1.30; Wu2024: g=-1.28, 95% CI -1.66 to -0.91)

Inflammatory Markers:
- **TNF-α**: Elevated across eating disorders (Dani2024: SMD 0.64, 95%

CI 0.11–1.17)
- **IL-1β**: Significantly increased (Dani2024: SMD 0.63, 95% CI 0.02–1.24)
- **Bone metabolism markers**: OPG and sRANKL elevated in AN (Dani2024: OPG SMD 1.92, 95% CI 0.96–2.88)

Cardiac Abnormalities:
- **Structural changes**: Reduced left ventricular mass (Smythe2021: SMD 1.82, 95% CI 1.32–2.31) and cardiac output (SMD 1.92, 95% CI 1.38–2.45)
- **Functional preservation**: Global longitudinal strain preserved despite structural changes (Gherbesi2024: no significant difference in GLS)

Genetic Associations:
- **Population-specific effects**: 5-HTR2A polymorphisms associated with AN risk only in Caucasian populations (Bevilacqua2024: Italian studies OR=1.82, 95% CI 1.41–2.37; Yan2021: Caucasian allele model OR=1.31, 95% CI 1.11–1.64)

Treatment and Clinical Findings

Medical Stability:
- Medical instability occurs across weight ranges, with 29–42% of non-underweight adolescents requiring hospitalization (Brennan2023)
- Rapid weight loss more predictive than absolute weight for medical complications

Treatment Efficacy:
- **DHEA supplementation**: No benefit as monotherapy for bone health (Lin2022)
- **Non-invasive brain stimulation**: Moderate effectiveness (Xu2022: g=-0.48) with dose-response relationship

Prevalence:
- Global childhood eating disorder prevalence: 1.0% (95% CI 0.6–1.6), with pica most common at 2.1% (Salari2025)

Constitutional Thinness Distinction

Constitutional thinness represents a distinct phenotype from AN (Bailly2021), characterized by:
- Higher BMI than AN (SMD 1.12, 95% CI 0.75–1.49) but lower than controls (SMD 4.16, 95% CI 3.53–4.79)
- Normal energy intake and preserved hormonal function
- "Underweight but not underfat" body composition

3. Population Analysis

Gender Distribution

- **Articles with predominantly/exclusively female samples**: Bailly2021 (27/39 studies female-only), Gherbesi2024 (88-100% female), Lin2022 (women only), Shobeiri2022 (100% female), Thavaraputta2023 (female only), Tural2022 (98%+ female), Wu2024 (100% female)
- **Articles with mixed gender**: Bailly2021 (9/39 studies both genders), Bevilacqua2024 (mixed), Karageorgiou2020 (one study included men)
- **Articles not reporting gender**: Brennan2023, Dani2024, Keeler2022, Li2022, Mai2022, Salari2025, Seidel2021, Smythe2021, Xu2022, Yan2021

Socioeconomic Status
No articles reported SES data systematically - representing a significant demographic gap across all studies.

Race and Ethnicity
- **Articles reporting race/ethnicity**: Bevilacqua2024 (Caucasian and Asian populations), Lin2022 (limited reporting: 87-100% White), Wu2024 (limited: 9 studies 100% Caucasian, 3 studies 100% Asian), Yan2021 (Caucasian and Asian subgroups)
- **Articles not reporting race/ethnicity**: All remaining 15 articles
- **Notable gap**: Severe underrepresentation of diverse populations across the literature

4. Study Comparison

Similarities in Design and Methodology
- **Systematic reviews/meta-analyses**: 18 of 19 articles used systematic review methodology with comprehensive database searches
- **Quality assessment tools**: Newcastle-Ottawa Scale commonly used (Bevilacqua2024, Gherbesi2024, Seidel2021)
- **Statistical approaches**: Random-effects models predominant due to high heterogeneity
- **PRISMA compliance**: Multiple studies explicitly followed PRISMA guidelines

Differences in Populations and Settings
- **Age ranges**: Pediatric focus (Brennan2023, Salari2025) vs. adult populations (majority of studies)
- **Geographic scope**: Single-country studies vs. international meta-analyses
- **Clinical settings**: Inpatient vs. outpatient vs. mixed populations
- **Diagnostic criteria**: DSM-IV vs. DSM-5 vs. ICD-10 variations across studies

Convergent Findings
Strong convergence across studies:
- Leptin consistently decreased (Karageorgiou2020, Wu2024, Bailly2021)
- Ghrelin consistently elevated (Seidel2021, Wu2024)

- Cortisol consistently elevated (Thavaraputta2023, Wu2024, Bailly2021)
- BDNF consistently decreased (Keeler2022, Shobeiri2022)
- Cardiac structural abnormalities consistent (Smythe2021, Gherbesi2024)

Divergent Findings
- **Inflammatory markers**: Some studies found elevated TNF-α and IL-1β (Dani2024) while others found no significant CRP or IL-6 differences
- **Olfactory function**: Largely intact (Mai2022) contrary to some individual study expectations
- **Genetic associations**: Population-specific effects with no universal genetic risk factors (Bevilacqua2024, Yan2021)

Conclusion Similarities
Most studies conclude that:
- Biological abnormalities in eating disorders reflect starvation adaptation rather than primary pathology
- Many abnormalities are reversible with weight restoration
- Individual biomarkers have limited diagnostic utility due to overlap between groups
- Multi-modal approaches needed for clinical applications

Conclusion Differences
- **Treatment implications**: Some studies support specific interventions (brain stimulation) while others argue against them (DHEA supplementation)
- **Clinical utility**: Varying optimism about biomarker clinical applications
- **Causality**: Disagreement about whether findings represent causes or consequences of eating disorders

5. Article-Specific Information

Article 1: Bailly2021
- **Objective**: Characterize constitutional thinness vs. anorexia nervosa
- **Design**: Meta-analysis of 39 clinical trials
- **Population**: Mixed gender, 27 studies female-only
- **Key findings**: CT distinct from AN with BMI SMD 1.12 (0.75-1.49) higher than AN, normal energy intake
- **Limitations**: Heterogeneous diagnostic criteria, limited longitudinal data

Article 2: Bevilacqua2024
- **Objective**: Examine serotonin receptor polymorphisms in anorexia nervosa
- **Design**: Meta-analysis of 18 genetic association studies
- **Population**: Predominantly female, mixed Caucasian/Asian

- **Key findings**: 5-HTR2A rs6311 associated with AN only in Southern Europeans (OR=1.82, 1.41-2.37)
- **Limitations**: Population stratification, limited clinical applicability

Article 3: Brennan2023
- **Objective**: Compare medical instability in typical vs. atypical anorexia nervosa
- **Design**: Meta-analysis of 9 studies (375 participants)
- **Population**: Adolescents, mostly female
- **Key findings**: 29-42% medical instability regardless of weight status
- **Limitations**: Small number of studies, limited ethnic diversity

Article 4: Dani2024
- **Objective**: Examine inflammatory biomarkers across eating disorders
- **Design**: Meta-analysis of 51 studies
- **Population**: Mixed eating disorders, gender not specified
- **Key findings**: TNF-α SMD 0.64 (0.11-1.17), IL-1β SMD 0.63 (0.02-1.24) elevated
- **Limitations**: Significant heterogeneity, limited longitudinal data

Article 5: Gherbesi2024
- **Objective**: Assess myocardial mechanics in anorexia nervosa
- **Design**: Meta-analysis of 5 studies (171 AN, 147 controls)
- **Population**: 88-100% female across studies
- **Key findings**: Preserved global longitudinal strain despite structural changes
- **Limitations**: Small number of studies, limited geographic diversity

Article 6: Karageorgiou2020
- **Objective**: Synthesize adipokine dysregulation in anorexia nervosa
- **Design**: Meta-analysis of 107 studies (>3000 AN patients)
- **Population**: Primarily female
- **Key findings**: Leptin SMD -3.03 to -3.84, adiponectin SMD 1.74 (1.01-2.47)
- **Limitations**: High heterogeneity, publication bias

Article 7: Keeler2022
- **Objective**: Examine growth factors in anorexia nervosa
- **Design**: Meta-analysis of 82 studies (56 cross-sectional, 24 longitudinal)
- **Population**: Primarily female, 14 countries
- **Key findings**: BDNF SMD -0.51 (-0.82 to -0.21), IGF-1 SMD -1.57 (-1.84 to -1.30)
- **Limitations**: High heterogeneity, variable follow-up periods

Article 8: Li2022
- **Objective**: Examine gut microbiota diversity in neurological diseases
- **Design**: Meta-analysis of 24 studies (2,758 subjects)
- **Population**: Mixed neurological conditions, global scope
- **Key findings**: AN showed increased alpha diversity (Shannon SMD 0.37, 0.04-0.69)
- **Limitations**: Technical heterogeneity, limited AN-specific studies

Article 9: Lin2022
- **Objective**: Assess DHEA status and supplementation in anorexia nervosa
- **Design**: Meta-analysis of 22 studies (15 observational, 7 RCTs)
- **Population**: Women only, limited ethnic diversity
- **Key findings**: Elevated DHEA (MD 311.63 ng/dl), no benefit from supplementation
- **Limitations**: Age-dependent effects, potential harm in younger patients

Article 10: Mai2022
- **Objective**: Examine olfactory function as biomarker for anorexia nervosa
- **Design**: Meta-analysis of 14 studies (818 participants)
- **Population**: 96.5% female
- **Key findings**: Largely intact olfactory function, BMI moderated threshold (β=0.79)
- **Limitations**: High heterogeneity, limited male representation

Article 11: Salari2025
- **Objective**: Determine global prevalence of eating disorders in children
- **Design**: Meta-analysis of 19 studies (448,173 children)
- **Population**: Children aged 3-14 years, global scope
- **Key findings**: Overall prevalence 1.0% (0.6-1.6%), pica most common at 2.1%
- **Limitations**: High heterogeneity, limited African representation

Article 12: Seidel2021
- **Objective**: Examine ghrelin levels in anorexia nervosa
- **Design**: Meta-analysis of 49 studies (1,031 AN, 1,002 controls)
- **Population**: 96% female studies
- **Key findings**: All ghrelin forms elevated (total SMD 2.51, 1.68-3.33)
- **Limitations**: Variable assay methods, limited long-term follow-up

Article 13: Shobeiri2022
- **Objective**: Examine BDNF levels in eating disorders
- **Design**: Meta-analysis of 15 studies (795 ED, 552 controls)

- **Population**: 100% female
- **Key findings**: Lower BDNF (SMD -0.49, -0.89 to -0.08)
- **Limitations**: High heterogeneity, no male participants

Article 14: Smythe2021
- **Objective**: Define cardiac abnormalities in anorexia nervosa
- **Design**: Meta-analysis of 23 studies (960 patients)
- **Population**: Mean age 17 years, primarily female
- **Key findings**: Reduced LV mass (SMD 1.82, 1.32-2.31), cardiac output (SMD 1.92, 1.38-2.45)
- **Limitations**: Heterogeneous designs, limited geographic diversity

Article 15: Thavaraputta2023
- **Objective**: Quantify adrenocortical hormone levels in anorexia nervosa
- **Design**: Meta-analysis of 101 studies (>2500 females)
- **Population**: Female only
- **Key findings**: Elevated cortisol (morning MD 4.58 μg/dL), reduced DHEA-S (MD -19.45 μg/dL)
- **Limitations**: Observational studies only, no race/ethnicity data

Article 16: Tural2022
- **Objective**: Examine adiponectin levels and modifiers in anorexia nervosa
- **Design**: Meta-regression of 34 studies (1,863 participants)
- **Population**: 98%+ female
- **Key findings**: Elevated adiponectin (g=0.765, 0.599-0.932), multiple metabolic modifiers
- **Limitations**: Limited male representation, cross-sectional data

Article 17: Wu2024
- **Objective**: Comprehensive analysis of peripheral biomarkers in anorexia nervosa
- **Design**: Meta-analysis of 123 studies (5,401 participants)
- **Population**: 100% female
- **Key findings**: 26 of 52 biomarkers significant, leptin most decreased (g=-1.62)
- **Limitations**: Limited ethnic diversity, cross-sectional design

Article 18: Xu2022
- **Objective**: Evaluate non-invasive brain stimulation for eating disorders
- **Design**: Meta-analysis of 15 studies (325 participants)
- **Population**: Mixed eating disorders
- **Key findings**: Moderate effectiveness (g=-0.48), dose-response relationship
- **Limitations**: Small sample sizes, limited long-term follow-up

Article 19: Yan2021
- **Objective**: Examine 5-HTR2A polymorphism association with eating

disorders
- **Design**: Meta-analysis of 17 studies (AN: 2,028 cases; BN: 505 cases)
- **Population**: Caucasian and Asian populations
- **Key findings**: AN association only in Caucasians (allele OR 1.31, 1.11-1.64)
- **Limitations**: Ethnic stratification, limited clinical utility

SUMMARY FOR ARTICLE GROUP 11

Cluster Analysis Results: articles
Number of articles: 18
==

SYSTEMATIC UMBRELLA REVIEW CLUSTER SYNTHESIS

1. Overall Cluster Label
Eating Disorders and Comorbid Conditions

Subcategories Identified:
- **Prevalence Studies**: Population-based prevalence estimates across age groups and populations
- **Medical Comorbidity**: Associations with diabetes, celiac disease, pregnancy outcomes
- **Psychiatric Comorbidity**: Co-occurrence with mood disorders, personality disorders, substance use
- **Developmental Impact**: Effects on growth, puberty, and life transitions
- **Trauma and Risk Factors**: Childhood maltreatment and environmental influences

2. Overall Findings Summary

Primary Prevalence Findings
Children and Adolescents:
- Overall eating disorder prevalence in children: 1.0% (95% CI: 0.6-1.6) [Salari2025]
- Binge eating disorder in youth: 1.32% (95% CI: 0.009-0.018) [Kjedberg]
- Rumination disorder in children: 0.4-1.0% depending on criteria [Haworth2023]

Special Populations:
- Pregnancy: 4.3% (95% CI: 2%-9%) overall ED prevalence [Ozturk2024]
- Pica in pregnancy: 28% (95% CI: 17-30%) [Sanjari2023]
- Type 1 diabetes: 14.35% (95% CI: 10.65-18.05) eating disorders via diagnostic interviews [Benton2023]
- Transgender individuals: 17.70% (95% CI: 16.32-19.08) overall ED prevalence [Rasmussen2023]

Medical Comorbidity Associations
Diabetes-Eating Disorder Relationships:
- Type 1 diabetes patients show 2.47 times higher risk of eating disorders (95% CI: 1.84-3.32) [Dean2024]
- Insulin omission/misuse affects 10.3% (95% CI: 8.1-13%) of T1DM patients [Dean2024]
- Mental disorders in T1DM: Depression 14.35% (95% CI: 10.65-18.05), Anxiety 12.56% (95% CI: 8.87-16.24) [Benton2023]

Pregnancy and Birth Outcomes:
- Anorexia nervosa increases low birth weight risk: OR 1.74 (95% CI: 1.49-2.03) [Milembamane2024]
- Bulimia nervosa increases preterm birth risk: OR 1.19 (95% CI: 1.04-1.36) [Milembamane2024]
- Binge eating disorder increases large for gestational age risk: OR 1.43 (95% CI: 1.18-1.72) [Milembamane2024]
- Anorexia nervosa reduces gestational diabetes risk: OR 0.63 (95% CI: 0.49-0.80) [Wilson2022]

Psychiatric Comorbidity
Bipolar Disorder Co-occurrence:
- Anorexia nervosa in bipolar disorder: 3.8% (95% CI: 2-6%) [Fornaro2021]
- Bulimia nervosa in bipolar disorder: 7.4% (95% CI: 6-10%) [Fornaro2021]
- Binge eating disorder in bipolar disorder: 12.5% (95% CI: 9.4-16.6%) [Fornaro2021]

Personality Disorder Comorbidity:
- Personality disorders in binge eating disorder: 27.9% (95% CrI: 0.22-0.34) [Senra2023]
- Borderline personality symptoms most elevated in eating disorders, particularly affective instability (g = 1.49, 95% CI: 1.26-1.71) [Miller2022]

Trauma and Risk Factors
Childhood Maltreatment:
- Overall association with eating disorders: OR 2.37 (95% CI: 1.84-3.06) [Amiri2025]
- Sexual abuse shows strongest association: OR 2.18 (95% CI: 1.59-3.0) [Amiri2025]
- Physical neglect: OR 2.28 (95% CI: 1.56-3.32) [Amiri2025]

Exercise Addiction:
- 3.71 times higher odds in those with eating disorders (95% CI: 2.00-6.89) [Trott2021]

Developmental and Long-term Outcomes
Growth and Development:
- No significant height differences between anorexia nervosa cases and

controls in meta-analysis [Neale2020]
- Eating disorder persistence from adolescence to adulthood: 40.7% [Fillipponi2022]
- Relapse rate: 24.5% [Fillipponi2022]

3. Population Analysis

Gender Distribution
Articles reporting gender data: All 18 articles provided gender information
- **Predominantly female samples (>80%):** Fillipponi2022 (89-100%), Ozturk2024 (100%), Milembamane2024 (100%), Sanjari2023 (100%), Wilson2022 (100%)
- **Mixed gender samples:** Amiri2025, Benton2023, Dean2024, Fornaro2021, Haworth2023, Kjedberg, Miller2022, Neale2020, Nikniaz2021, Senra2023 (77.4% female), Trott2021 (70.6% female)
- **Transgender-specific:** Rasmussen2023 (mixed transgender and cisgender populations)

Socioeconomic Status
Articles including SES data: Only 2 articles (Ozturk2024, Sanjari2023)
- Ozturk2024: 24.5% primary education, 48.2% secondary, 29.9% higher education
- Sanjari2023: Mixed employment status and education levels
- **Notable gap:** 16 of 18 articles did not systematically report SES data

Race and Ethnicity
Articles reporting race/ethnicity: Only 3 articles provided detailed racial/ethnic data
- Miller2022: Predominantly Caucasian participants
- Nikniaz2021: Limited reporting (Nordic, Caucasian/Finnish, White populations)
- Sanjari2023: Global sample including African, American, and Asian populations
- **Critical gap:** 15 of 18 articles lacked comprehensive racial/ethnic reporting

Notable Demographic Biases
- **Gender bias:** Severe underrepresentation of males across most studies
- **Geographic bias:** Predominantly Western, high-income countries
- **Age bias:** Limited representation of older adults and very young children
- **Racial/ethnic bias:** Overwhelming focus on White/Caucasian populations

4. Study Comparison

Similarities in Design and Methodology
Systematic review and meta-analysis approach: All 18 articles used systematic review methodology with meta-analytic synthesis
Common databases searched: PubMed/MEDLINE (all studies), PsycINFO (16 studies), EMBASE (12 studies)
Quality assessment tools: Newcastle-Ottawa Scale (8 studies), JBI tools (3 studies), PRISMA guidelines (15 studies)
Statistical approaches: Random-effects models predominated due to high heterogeneity

Differences in Populations and Settings
Age-focused studies:
- **Pediatric focus:** Salari2025, Kjedberg, Neale2020, Fillipponi2022
- **Adult focus:** Senra2023, Miller2022, Trott2021
- **Reproductive-age women:** Ozturk2024, Milembamane2024, Wilson2022, Sanjari2023
- **Lifespan approach:** Fornaro2021, Haworth2023

Setting differences:
- **Community-based:** Salari2025, Haworth2023, Rasmussen2023
- **Clinical samples:** Benton2023, Dean2024, Senra2023
- **Population registries:** Wilson2022, Milembamane2024

Convergent vs. Divergent Findings

Convergent findings:
- **High comorbidity rates:** Benton2023, Fornaro2021, Senra2023, Miller2022 all demonstrate elevated psychiatric comorbidity
- **Female predominance:** Consistent across Dean2024, Fornaro2021, Trott2021, Rasmussen2023
- **Bidirectional relationships:** Nikniaz2021, Dean2024, Fornaro2021 show bidirectional medical-psychiatric associations
- **Trauma associations:** Amiri2025 findings consistent with psychiatric comorbidity patterns in Miller2022, Senra2023

Divergent findings:
- **Prevalence estimates vary dramatically** by population: 1% in children [Salari2025] vs. 17.7% in transgender individuals [Rasmussen2023]
- **Anorexia nervosa effects:** Protective for gestational diabetes [Wilson2022] but increases birth complications [Milembamane2024]
- **Recovery patterns:** Fillipponi2022 shows 40% persistence vs. Neale2020 suggests growth recovery possible

Similarities and Differences in Conclusions

Similar conclusions:
- **Screening importance:** Benton2023, Dean2024, Nikniaz2021, Ozturk2024 all emphasize need for systematic screening
- **Integrated care:** Wilson2022, Milembamane2024, Benton2023

advocate for multidisciplinary approaches
- **High-risk populations:** Rasmussen2023, Fornaro2021, Senra2023 identify vulnerable subgroups requiring targeted interventions

Differing conclusions:
- **Intervention timing:** Neale2020 emphasizes early intervention for reversibility vs. Fillipponi2022 suggests chronic course
- **Treatment focus:** Miller2022 emphasizes emotion regulation vs. Trott2021 focuses on exercise monitoring
- **Population-specific needs:** Rasmussen2023 highlights gender-affirming care vs. Ozturk2024 emphasizes perinatal care

5. Article-Specific Information

Article 1: Amiri2025
- **Objective:** Quantify association between childhood maltreatment and eating disorders
- **Design:** Meta-analysis of 30 observational studies
- **Population:** Mixed gender, various ages
- **Key Finding:** OR 2.37 (95% CI: 1.84-3.06) overall association
- **Subgroup Results:** Sexual abuse OR 2.18 (95% CI: 1.59-3.0); Physical neglect OR 2.28 (95% CI: 1.56-3.32)
- **Limitations:** High heterogeneity (I^2=92.6%), limited demographic reporting

Article 2: Benton2023
- **Objective:** Determine mental disorder prevalence in type 1 diabetes
- **Design:** Meta-analysis of 38 studies from 14 countries
- **Population:** Type 1 diabetes patients, mixed gender
- **Key Finding:** Depression 14.35% (95% CI: 10.65-18.05), Anxiety 12.56% (95% CI: 8.87-16.24)
- **Subgroup Results:** Diagnostic interviews vs. clinical registers showed 24-fold differences
- **Limitations:** Substantial heterogeneity, limited demographic data

Article 3: Dean2024
- **Objective:** Examine eating disorder risk in type 1 diabetes
- **Design:** Meta-analysis of 14 controlled studies
- **Population:** 9,079 participants (1,391 T1DM, 7,688 controls)
- **Key Finding:** RR 2.47 (95% CI: 1.84-3.32) overall eating disorder risk
- **Subgroup Results:** Insulin omission 10.3% (95% CI: 8.1-13%); Female vs. male insulin misuse RR 6.51 (95% CI: 1.14-37.31)
- **Limitations:** Predominantly Western populations, varied assessment tools

Article 4: Fillipponi2022
- **Objective:** Examine eating disorder persistence from adolescence to adulthood

- **Design:** Meta-analysis of 16 studies (15 populations)
- **Population:** 89-100% female, Western countries only
- **Key Finding:** 40.7% persistence rate, 24.5% relapse rate
- **Subgroup Results:** Mean duration 3.4 years, highest relapse risk in first year
- **Limitations:** Female-only samples, Western populations, varied outcome definitions

Article 5: Fornaro2021
- **Objective:** Examine bidirectional bipolar disorder-eating disorder comorbidity
- **Design:** Meta-analysis of 47 articles, 77 comparisons
- **Population:** 15,084 BD patients, 15,146 ED patients
- **Key Finding:** BED in BD 12.5% (95% CI: 9.4-16.6%), BN in BD 7.4% (95% CI: 6-10%)
- **Subgroup Results:** BD-II higher ED rates than BD-I; Female gender OR 6.25 for AN comorbidity
- **Limitations:** High heterogeneity, limited longitudinal data

Article 6: Haworth2023
- **Objective:** Determine global rumination syndrome/disorder prevalence
- **Design:** Meta-analysis of 30 studies, 114,228 participants
- **Population:** Mixed ages, global sample
- **Key Finding:** Adults 3.7% (95% CI: 2.3-5.1), Children 0.4-1.0% depending on criteria
- **Subgroup Results:** Female gender OR 1.4 in adults; Anxiety OR 2.3, Depression OR 1.8
- **Limitations:** High heterogeneity, poor statistical analysis in 86% of studies

Article 7: Kjedberg
- **Objective:** Determine binge eating disorder prevalence in youth
- **Design:** Meta-analysis of 39 studies, 52 samples
- **Population:** 73,164 children/adolescents up to age 20
- **Key Finding:** BED 1.32% (95% CI: 0.009-0.018), Subclinical BED 3.0%
- **Subgroup Results:** Overweight samples 5% vs. normal weight 1%; Interview methods higher than self-report
- **Limitations:** Extremely high heterogeneity (I^2>95%), limited age effects

Article 8: Milembamane2024
- **Objective:** Examine maternal eating disorders and birth outcomes
- **Design:** Meta-analysis of 18 studies, 2,369,520 women
- **Population:** Pregnant women, predominantly European studies
- **Key Finding:** AN-low birth weight OR 1.74 (95% CI: 1.49-2.03)
- **Subgroup Results:** BN-preterm birth OR 1.19 (95% CI: 1.04-1.36); BED-large for gestational age OR 1.43 (95% CI: 1.18-1.72)
- **Limitations:** Developed countries only, underrepresentation of

ethnic minorities

Article 9: Miller2022
- **Objective:** Examine borderline personality disorder symptoms in eating disorders
- **Design:** Meta-analysis of 122 studies, 36,688 participants
- **Population:** 75.77-99.85% female depending on analysis
- **Key Finding:** Affective instability largest effect size g=1.49 (95% CI: 1.26-1.71)
- **Subgroup Results:** AN binge-purging subtype highest BPD symptoms; Anger lowest effect size g=0.46
- **Limitations:** Limited diversity, cross-sectional studies, state vs. trait measurement issues

Article 10: Neale2020
- **Objective:** Examine growth and pubertal effects of restrictive eating disorders
- **Design:** Meta-analysis of 27 observational studies
- **Population:** Children <18 years, predominantly female
- **Key Finding:** No significant height difference between AN cases and controls (-0.13 cm, 95% CI: -2.64 to 2.38)
- **Subgroup Results:** Evidence for catch-up growth in majority but not all cases
- **Limitations:** Small sample sizes, predominantly female focus, varied methodologies

Article 11: Nikniaz2021
- **Objective:** Examine bidirectional celiac disease-eating disorder relationship
- **Design:** Meta-analysis of 23 observational studies
- **Population:** Mixed gender (55-100% female across studies)
- **Key Finding:** EDs in CD 8.88% (95% CI: 6.4-11.7); CD in AN patients RR 2.35 (95% CI: 1.27-3.44)
- **Subgroup Results:** AN risk in CD patients RR 1.48 (95% CI: 1.32-1.64)
- **Limitations:** Limited ethnic diversity, methodological heterogeneity

Article 12: Ozturk2024
- **Objective:** Determine eating disorder prevalence in pregnancy
- **Design:** Meta-analysis of 11 studies, 2,369,520 pregnant women
- **Population:** Pregnant women, global sample
- **Key Finding:** Overall ED prevalence 4.3% (95% CI: 2%-9%)
- **Subgroup Results:** 61.6% <30 years old; 49.4% anxiety, 30.7% depression comorbidity
- **Limitations:** High heterogeneity (I^2=99.5%), limited generalizability

Article 13: Rasmussen2023
- **Objective:** Examine eating disorders in transgender individuals

- **Design:** Meta-analysis of 24 studies
- **Population:** Transgender and cisgender comparison groups
- **Key Finding:** Overall ED prevalence 17.70% (95% CI: 16.32–19.08)
- **Subgroup Results:** Transgender men 19.46% vs. transgender women 14.72%; Gender-affirming treatment may reduce symptoms
- **Limitations:** Limited diversity, cross-sectional studies, varied assessment methods

Article 14: Salari2025
- **Objective:** Determine global eating disorder prevalence in children
- **Design:** Meta-analysis of 19 studies, 448,173 children
- **Population:** Children aged 3–14 years
- **Key Finding:** Overall prevalence 1% (95% CI: 0.6–1.6)
- **Subgroup Results:** Pica 2.1%, BED 1%, AN 0.6%, BN 0.1%; Australia highest at 5.2%
- **Limitations:** High heterogeneity (I^2=99.1%), limited demographic data

Article 15: Sanjari2023
- **Objective:** Determine pica prevalence in pregnancy
- **Design:** Meta-analysis of 45 studies, 21,156 pregnant women
- **Population:** Global sample of pregnant women
- **Key Finding:** Overall prevalence 28% (95% CI: 17–30%)
- **Subgroup Results:** Rural 43% vs. urban 36%; Primary education 40% vs. higher education 29%
- **Limitations:** High heterogeneity (I^2=99.3%), varied cultural contexts

Article 16: Senra2023
- **Objective:** Examine personality disorder prevalence in binge eating disorder
- **Design:** Bayesian meta-analysis of 20 studies, 2,945 BED patients
- **Population:** 77.4% female, mean age 19–49.5 years
- **Key Finding:** 27.9% probability of comorbid personality disorder (95% CrI: 0.22–0.34)
- **Subgroup Results:** Borderline PD 24.7%, Obsessive-compulsive PD 35.4%, Avoidant PD 36.9%
- **Limitations:** High heterogeneity, cross-sectional studies, limited demographic diversity

Article 17: Trott2021
- **Objective:** Compare exercise addiction in those with/without eating disorders
- **Design:** Meta-analysis of 9 studies, 2,140 participants
- **Population:** 70.6% female overall, mean age 25.06
- **Key Finding:** OR 3.71 (95% CI: 2.00–6.89) for exercise addiction in ED group
- **Subgroup Results:** Effect varied by measurement tool (OEQ highest OR 6.9, EDQ lowest OR 2.4)

- **Limitations:** Substantial heterogeneity (I^2=81%), questionnaire-based ED assessment

Article 18: Wilson2022
- **Objective:** Examine gestational diabetes risk in women with preconception mental disorders
- **Design:** Meta-analysis of 22 observational studies
- **Population:** Women of reproductive age, predominantly White populations
- **Key Finding:** Schizophrenia OR 2.44 (95% CI: 1.17–5.11); AN OR 0.63 (95% CI: 0.49–0.80)
- **Subgroup Results:** No significant associations for depression, bulimia nervosa, or bipolar disorder
- **Limitations:** High-income country bias, varied SES reporting, high risk of bias in 14/22 studies

SUMMARY FOR ARTICLE GROUP 12

Cluster Analysis Results: articles
Number of articles: 17

==

FINAL SYNTHESIS: SYSTEMATIC UMBRELLA REVIEW CLUSTER ANALYSIS

1. Overall Cluster Label
Eating Disorders Systematic Reviews Meta-Analysis

Subcategories Identified:
- **Clinical Manifestations & Comorbidities** (sleep, oral health, physical activity, bone health)
- **Diagnostic Classification & Severity** (OSFED, atypical AN, diagnostic validity)
- **Neurodevelopmental Associations** (autism spectrum traits)
- **Nutritional & Physiological Patterns** (dietary intake, BMI relationships)
- **Epidemiological Patterns** (incidence rates)
- **Emotion Regulation & Psychological Functioning**

2. Overall Findings Summary

Primary Clinical Manifestations

Sleep Disturbances:
- AN patients show significant sleep impairments: reduced total sleep time (MD = −21.0 min, 95% CI: −40.8, −1.3), decreased sleep efficiency (MD = −3.30%, 95% CI: −6.8, −0.8), and altered sleep architecture with increased Stage 1 sleep (2.30%, 95% CI: 0.04, 4.6) and reduced REM sleep (−2.10%, 95% CI: −4.2, −0.02) (Burger2024, Degasperi2024)
- Sleep problems persist after weight restoration, indicating need for targeted sleep interventions (Burger2024)

Oral Health Complications:
- Dramatic increase in dental erosion across EDs (OR = 15.27, 95% CI: 9.92, 23.51) with bulimia showing highest rates (54.4%, 95% CI: 42.3–66.3) versus anorexia (26.7%, 95% CI: 14.5–41.1) (Chan2024, Nijakowski2023)
- Self-induced vomiting creates 16-fold increased risk for dental erosion (OR = 16.176, 95% CI: 1.438–181.918) (Nijakowski2023)
- AN patients show significantly higher caries experience (DMFT = 2.00, 95% CI: 0.87–3.13), increased gingival inflammation (BOP = 16.57%, 95% CI: 12.89–20.25%), and reduced salivary flow (-0.27 ml/min, 95% CI: -0.31 to -0.24) (Niederau2025)

Bone Health & Body Composition:
- Severe bone mineral density deficits across all ED types, with AN showing largest effects: total body BMD (SMD = -3.45, 95% CI: -4.57 to -2.33), spine BMD (SMD = -4.34, 95% CI: -5.27 to -3.40), hip BMD (SMD = -4.95, 95% CI: -6.78 to -3.12) (Lopes2022)
- Low BMI, reduced fat/lean mass, and amenorrhea significantly predict bone density loss (Lopes2022)

Neurodevelopmental Associations

Autism Spectrum Traits:
- Significant elevation of autistic traits in AN across age groups: adults (g = 0.77, 95% CI: 0.54–1.01), adolescents/young adults (d = 0.79, 95% CI: 0.63–0.96) (Inal_Kaleli, Mulligan2021)
- 18% of AN patients score above ADOS cutoff for potential ASD diagnosis (95% CI: 0.08–0.27) (Inal_Kaleli)
- ASD-related neuropsychological characteristics worsen with illness duration, becoming significant only after 7+ years: cognitive inflexibility (SMD = -0.63, 95% CI: -0.78 to -0.48), weak central coherence (SMD = -0.72, 95% CI: -1.03 to -0.41), emotion recognition deficits (SMD = -0.64, 95% CI: -0.93 to -0.34) (Saure2020)

Diagnostic Classification Findings

OSFED Clinical Significance:
- OSFED subtypes demonstrate comparable severity to full-threshold EDs, with atypical AN showing higher eating pathology than AN (g = 0.41, $p < 0.001$) and night eating syndrome showing large effects versus controls (g = 1.07, $p = 0.007$) (Dang2024)
- Atypical AN patients exhibit higher drive for thinness (g = -0.40, 95% CI: -0.71 to -0.09), body dissatisfaction (g = -0.24, 95% CI: -0.40 to -0.08), and weight/shape concerns than AN patients (Johnson_Munguia2023)
- DSM-5 criteria fail to adequately distinguish between full and other-specified EDs in clinical severity, with OSFED sometimes showing greater impairment (Wilkop2023)

Nutritional & Physiological Patterns

Dietary Intake:
- Clear diagnostic distinctions: AN (1,311 kcal/day, 95% CI: 1,114–1,509), BED (2,429 kcal/day, 95% CI: 2,275–2,583), BN (2,696 kcal/day, 95% CI: 2,008–3,383) (Jenkins2024)
- Universal vitamin D deficiency across all EDs (0.18–5.8 μg/day vs. RDI 10 μg/day) (Jenkins2024)

Physical Activity:
- AN patients show distinct activity patterns by treatment stage: inpatients have highest sedentary time (665.52 min/day) but also highest MVPA (119.44 min/day), challenging "hyperactivity" assumptions (Pagador2025)

Emotion Regulation:
- AN shows specific deficits in adaptive emotion regulation compared to BN: overall adaptive strategies (g = 0.24, 95% CI: 0.08–0.40), acceptance (g = 0.23, 95% CI: 0.03–0.43), reappraisal (g = 0.22, 95% CI: 0.04–0.41) (Puttevils2021)

Epidemiological Patterns:
- AN incidence varies dramatically by detection method: hospital admissions (5.0/100,000 women-years) versus outpatient services (8.8/100,000 women-years), with young women showing 8-fold higher rates in outpatient settings (63.7/100,000) (Gonzalez2020)

3. Population Analysis

Gender Distribution:
- **Female-only studies:** Burger2024, Gonzalez2020, Inal_Kaleli, Mulligan2021, Niederau2025, Lopes2022
- **Predominantly female (>90%):** Chan2024, Dang2024, Degasperi2024, Johnson_Munguia2023, Nijakowski2023, Pagador2025 (99%), Puttevils2021 (98%), Saure2020 (94.2%), Wilkop2023 (93%)
- **Notable male representation:** Walsh2024 found significantly greater male representation in atypical AN versus AN

Socioeconomic Status:
- **SES data reported:** Chan2024 (36% of studies matched for SES), Jenkins2024 (only 6/39 studies reported SES)
- **SES not reported:** 14 of 17 articles provided no systematic SES data
- **Notable SES gap:** Severe underreporting limits understanding of socioeconomic factors in ED presentation and outcomes

Race and Ethnicity:
- **Race/ethnicity data reported:** Chan2024 (limited - only 2 studies matched for race), Dang2024 (predominantly White, 59–100% across studies), Inal_Kaleli (72–95.8% White European), Jenkins2024 (only

9/39 studies reported), Walsh2024 (slightly more non-White individuals in atypical AN)
- **Race/ethnicity not reported:** 12 of 17 articles
- **Critical diversity gap:** Overwhelming representation of White, Western populations severely limits generalizability

Notable Demographic Biases:
- Systematic underrepresentation of males, racial/ethnic minorities, and non-Western populations across all studies
- Limited socioeconomic diversity reporting prevents understanding of SES-related factors
- Geographic concentration in Europe, North America, and Australia

4. Study Comparison

Similarities in Design and Methodology:
- **Systematic review/meta-analysis approach:** All 17 articles employed systematic review methodology with most conducting meta-analyses
- **Database search strategies:** Consistent use of PubMed/MEDLINE, with common inclusion of PsycINFO, Web of Science, and Embase (Articles 1-17)
- **Quality assessment:** Most employed standardized tools (Newcastle-Ottawa Scale, ROBINS-E, PRISMA guidelines)
- **Random-effects models:** Standard approach for meta-analyses across studies

Differences in Populations and Settings:
- **Age focus:** Mulligan2021 (adolescents/young adults <25), Saure2020 (illness duration categories), others mixed ages
- **Diagnostic scope:** Single disorder focus (Burger2024, Degasperi2024, Gonzalez2020, Inal_Kaleli, Mulligan2021, Niederau2025, Pagador2025, Saure2020) versus multiple EDs (Chan2024, Dang2024, Jenkins2024, Lopes2022, Nijakowski2023, Puttevils2021, Wilkop2023)
- **Geographic representation:** European-dominated (Niederau2025, Inal_Kaleli) versus international samples (Chan2024, Dang2024)

Convergent Findings:
- **OSFED severity:** Dang2024, Johnson_Munguia2023, Walsh2024, and Wilkop2023 all demonstrate OSFED conditions are not "subthreshold"
- **Autism-AN association:** Inal_Kaleli, Mulligan2021, and Saure2020 consistently show elevated autistic traits in AN
- **Sleep disturbances in AN:** Burger2024 and Degasperi2024 both demonstrate significant sleep impairments
- **Oral health complications:** Chan2024, Nijakowski2023, and Niederau2025 consistently show dental/oral problems across EDs

Divergent Findings:
- **Sleep patterns by ED type:** Degasperi2024 found no significant sleep differences in BN versus controls, while Burger2024 focused only

on AN
- **Physical activity patterns:** Pagador2025 challenges traditional "hyperactivity" assumptions in AN
- **Autism trait severity by age:** Mulligan2021 found smaller effect sizes in younger populations versus Inal_Kaleli's adult findings

Conclusion Similarities:
- Universal call for reconsidering diagnostic hierarchies and "subthreshold" classifications
- Emphasis on need for comprehensive, multidisciplinary assessment and treatment
- Recognition of EDs as serious medical conditions requiring specialized intervention
- Identification of significant research gaps in diversity and methodology

Conclusion Differences:
- **Treatment implications:** Some emphasize medical monitoring (bone health, oral health), others psychological interventions (emotion regulation, autism-informed care)
- **Research priorities:** Vary from epidemiological needs to intervention development to diagnostic refinement

5. Article-Specific Information

Article 1: Burger2024
- **Objective:** Characterize sleep patterns in AN patients through systematic review and meta-analysis
- **Design:** Systematic review and meta-analysis of 30 papers (~1,750 AN patients)
- **Population:** AN patients, predominantly female, varied ages
- **Key Findings:** TST reduced by 21 minutes (95% CI: -40.8, -1.3), sleep efficiency decreased by 3.30% (95% CI: -6.8, -0.8), sleep architecture alterations persist post-weight restoration
- **Limitations:** Limited diversity in samples, heterogeneity in sleep measurement methods

Article 2: Chan2024
- **Objective:** Comprehensive evaluation of oral health status across eating disorders
- **Design:** Systematic review and meta-analysis of 33 observational studies
- **Population:** ED patients versus healthy controls, predominantly female
- **Key Findings:** Dental erosion OR = 15.27 (95% CI: 9.92-23.51), caries SMD = 0.28 (95% CI: 0.08-0.48), reduced salivary flow
- **Subgroup Analyses:** BN showed 11.49 higher odds of erosion versus 8.12 for AN
- **Limitations:** Very low certainty evidence, observational designs only

Article 3: Dang2024
- **Objective:** First comprehensive meta-analysis comparing all OSFED subtypes to threshold EDs
- **Design:** Meta-analysis and systematic review of 33 studies, 20,452 participants
- **Population:** All OSFED groups versus AN/BN/BED and controls, majority female (48.9–100%)
- **Key Findings:** Atypical AN versus controls g = 0.85, NES versus controls g = 1.07, OSFED versus BN g = -0.24
- **Moderation Analyses:** Country significantly moderated multiple comparisons
- **Limitations:** Limited diversity (predominantly White, Western), insufficient power reporting (78% of studies)

Article 4: Degasperi2024
- **Objective:** Investigate sleep quality and circadian preferences in eating disorders
- **Design:** Systematic review and meta-analysis of 27 case-control studies
- **Population:** 711 ED patients, 653 healthy controls, predominantly female
- **Key Findings:** Sleep efficiency d = -0.78, total sleep time d = -0.38, PSQI d = 0.95, slow wave sleep d = -0.44
- **Subgroup Analyses:** AN showed significant impairments, BN showed no significant differences from controls
- **Limitations:** Limited BED studies, inconsistent treatment effects

Article 5: Gonzalez2020
- **Objective:** Systematic review and meta-analysis of AN incidence in women (1980–2019)
- **Design:** Systematic review and meta-analysis of 31 studies
- **Population:** Women only, primarily European and USA populations
- **Key Findings:** Hospital admissions 5.0/100,000 women-years (95% CI: 4.87–5.05), outpatient services 8.8/100,000 (95% CI: 7.83–9.80)
- **Limitations:** High heterogeneity (I^2 = 97.4%), methodological variations across studies

Article 6: Inal_Kaleli
- **Objective:** Assess autistic traits and ASD prevalence in AN populations
- **Design:** Systematic review and meta-analysis of 22 studies
- **Population:** 1,061 AN patients, 2,636 controls, 100% female in most studies
- **Key Findings:** Overall autistic traits g = 0.77 (95% CI: 0.54–1.01), 18% scored above ADOS cutoff (95% CI: 0.08–0.27)
- **Subgroup Analyses:** Social skills g = 0.60, communication g = 0.37, attention switching g = 0.42
- **Limitations:** Predominantly Caucasian samples (72–95.8%), limited diversity

Article 7: Jenkins2024
- **Objective:** First systematic quantitative synthesis of dietary intake across eating disorders
- **Design:** Systematic review and meta-analysis of 39 studies, >1,900 participants
- **Population:** Adults with AN/BN/BED, predominantly female (n=1,738 vs 131 male)
- **Key Findings:** AN 1,311 kcal/day (95% CI: 1,114–1,509), BED 2,429 kcal/day (95% CI: 2,275–2,583), BN 2,696 kcal/day (95% CI: 2,008–3,383)
- **Limitations:** Only 6/39 studies reported SES, 9/39 reported race/ethnicity

Article 8: Johnson_Munguia2023
- **Objective:** Compare psychological and clinical differences between atypical AN and AN
- **Design:** Systematic review and meta-analysis of 20 articles
- **Population:** AtypAN and AN patients, 96.8% female, predominantly white
- **Key Findings:** Overall ED psychopathology g = −0.26 (95% CI: −0.46 to −0.06), drive for thinness g = −0.40 (95% CI: −0.71 to −0.09)
- **Limitations:** Limited male and diverse population representation

Article 9: Lopes2022
- **Objective:** Meta-analysis of BMD, body composition, and amenorrhea in female ED patients
- **Design:** Systematic review and meta-analysis of 43 studies
- **Population:** 4,163 women with EDs across multiple diagnoses
- **Key Findings:** Total body BMD SMD = −2.62 (95% CI: −3.39 to −1.84), spine BMD SMD = −3.31 (95% CI: −3.98 to −2.63)
- **Meta-regression:** Low BMI ($\beta = 0.74$), low fat mass ($\beta = 0.26$), amenorrhea ($\beta = -0.04$) predicted lower BMD
- **Limitations:** Female-only samples, limited race/ethnicity reporting

Article 10: Mulligan2021
- **Objective:** Examine ASD traits in adolescents/young people with AN using AQ measures
- **Design:** Systematic review and meta-analysis of 7 studies
- **Population:** 1,634 participants (255 AN, 1,379 controls), female only, under 25 years
- **Key Findings:** Effect size d = 0.79 (95% CI: 0.63–0.96), smaller than adult samples
- **Limitations:** Female-only studies, no race/ethnicity data, limited longitudinal research

Article 11: Niederau2025
- **Objective:** Examine oral health parameters in AN from orthodontic perspective

- **Design:** Systematic review and meta-analysis of 11 studies
- **Population:** 345 AN patients, 312 controls, 100% female, mean age 18.4 years
- **Key Findings:** BOP MD = 16.57% (95% CI: 12.89-20.25%), DMFT MD = 2.00 (95% CI: 0.87-3.13), salivary flow MD = -0.27 ml/min (95% CI: -0.31 to -0.24)
- **Limitations:** Female-only populations, limited geographic diversity

Article 12: Nijakowski2023
- **Objective:** Examine relationship between eating disorders and dental erosion
- **Design:** Systematic review and meta-analysis of 31 studies, 17 countries
- **Population:** 1,699 ED participants, predominantly young women
- **Key Findings:** Overall erosion prevalence 42.1% (95% CI: 33.6-50.8), BN 54.4% (95% CI: 42.3-66.3), AN 26.7% (95% CI: 14.5-41.1)
- **Effect Sizes:** Self-induced vomiting OR = 16.176 (95% CI: 1.438-181.918)
- **Limitations:** Variable diagnostic criteria across studies, methodological heterogeneity

Article 13: Pagador2025
- **Objective:** Examine objectively measured physical activity levels in AN patients
- **Design:** Systematic review and meta-analysis of 16 studies
- **Population:** 726 participants (719 female, 7 male), predominantly Caucasian
- **Key Findings:** Sedentary time 617.49 min/day (95% CI: 535.7-699.27), MVPA 63.19 min/day (95% CI: 50.07-76.31)
- **Subgroup Analyses:** Adults showed higher LPA/MPA than teenagers; inpatients had highest sedentary time and MVPA
- **Limitations:** Limited generalizability due to predominantly female, Caucasian samples

Article 14: Puttevils2021
- **Objective:** Compare emotion regulation strategies between AN and BN
- **Design:** Systematic review and meta-analysis of 41 studies
- **Population:** 5,868 participants, ≥98% female, mean age 25.26 years
- **Key Findings:** Adaptive ER strategies g = 0.24 (95% CI: 0.08-0.40) favoring BN, acceptance g = 0.23 (95% CI: 0.03-0.43), reappraisal g = 0.22 (95% CI: 0.04-0.41)
- **Limitations:** Self-report measures only, predominantly female samples

Article 15: Saure2020
- **Objective:** Examine relationship between ASD characteristics and AN illness duration

- **Design:** Systematic review and meta-analysis of 53 studies
- **Population:** 2,142 AN participants (94.2% female), 2,244 controls (94.3% female)
- **Key Findings:** Prolonged illness (≥7 years) showed significant deficits: central coherence SMD = -0.72 (95% CI: -1.03 to -0.41), cognitive flexibility SMD = -0.63 (95% CI: -0.78 to -0.48)
- **Limitations:** Mostly Caucasian samples, cross-sectional designs limit causality inferences

Article 16: Walsh2024
- **Objective:** Compare clinical characteristics of atypical AN to AN and healthy controls
- **Design:** Systematic review and meta-analysis of 24 publications
- **Population:** Variable across studies, males more represented in atypical AN versus AN
- **Key Findings:** Large BMI differences between groups, comparable eating disorder psychopathology, less frequent menstrual disturbance in atypical AN, higher spinal BMD in atypical AN
- **Limitations:** Limited information on course and treatment outcomes, inconsistent demographic reporting

Article 17: Wilkop2023
- **Objective:** Examine impairments across DSM-5 eating disorders to assess diagnostic validity
- **Design:** Systematic review and multilevel meta-analysis of 69 studies
- **Population:** 20,651 participants (93% female), mean age 25.2 years
- **Key Findings:** No differences between AN and OSFED in eating/ general psychopathology, BN showed greater eating pathology than OSFED (g = 0.65), atypical AN showed greater eating pathology than AN (g = -0.25)
- **Limitations:** Significant underrepresentation of males (7%), very high heterogeneity, limited diversity

SUMMARY FOR ARTICLE GROUP 13

Cluster Analysis Results: articles
Number of articles: 14
==

FINAL SYNTHESIS: EATING DISORDERS COMORBIDITY AND PREVALENCE

1. Overall Cluster Label
Eating Disorders: Comorbidity and Global Prevalence

Subcategories identified:
- **Prevalence Studies** (Articles 1, 6, 11, 12, 13): Geographic and demographic prevalence patterns
- **Comorbidity Studies** (Articles 2, 3, 4, 5, 7, 8, 10, 14): Co-

occurring psychiatric conditions and behaviors
- **Treatment Innovation** (Article 9): Virtual reality interventions

2. Overall Findings Summary

Most Scientifically Relevant Findings:

Global Prevalence Patterns:
- Overall eating disorder lifetime prevalence: **1.69%** globally (Qian2021), with substantial geographic variation from **0.22%** in Asian countries to **1.89%** in Western countries
- Western Asia shows **22.07%** prevalence on EAT-26/40 screening but only **1.59% AN, 2.41% BN, 3.51% EDNOS** on clinical interviews (Alfalahi2022)
- Iran demonstrates protective cultural factors with **1%** lifetime prevalence versus **9%** in Western countries (Hashemi2022)
- Migrant populations show **55% reduced odds** (OR = 0.45, 95% CI: 0.35-0.59) of eating disorders compared to local populations (Siddiqi2024)

Mortality and Severity:
- Eating disorders carry severe mortality risk with overall **SMR = 3.39** (95% CI: 2.90-3.95), with anorexia nervosa showing highest risk at **SMR = 5.21** (95% CI: 4.10-6.62) (Krug2025)

Most Clinically Relevant Findings:

Self-Harm and Suicide Risk:
- **Non-suicidal self-injury** affects **40%** (95% CI: 33-46%) of individuals with eating disorders (Amiri2023), with youth showing **34.2%** prevalence (95% CI: 27.5-41.7%) (Meier2025)
- **Suicidal ideation** affects **51%** (95% CI: 41-62%) and **suicide attempts** occur in **22%** (95% CI: 18-25%) of eating disorder patients (Amiri2023)
- Compared to healthy controls, eating disorder patients show **6.85-fold increased odds** of NSSI and **5.16-fold increased odds** of suicide attempts (Sohn2023)

Substance Use and Behavioral Comorbidities:
- **Alcohol use** affects **26%** and **smoking** affects **30%** of individuals with eating disorders (Amiri2024)
- **Impulse control disorders** affect **22%** of eating disorder patients, with **compulsive buying (19%)** and **kleptomania (18%)** being most common (Devoe2022)
- **Psychotic symptoms** co-occur in **7%** (95% CI: 2-13%) of eating disorder cases (Drymonitou2024)

Subgroup Patterns:
- Binge-purge subtypes consistently show higher comorbidity rates: **BN patients show 44% NSSI** versus **29% in AN** (Amiri2023)

- **Bulimia nervosa** shows highest substance use rates: **28% alcohol use, 30% smoking** (Amiri2024)

3. Population Analysis

Gender Distribution:
- **Articles reporting gender data:** 1, 2, 3, 4, 5, 6, 7, 8, 10, 11, 12, 13, 14
- **Consistent pattern:** Predominantly female samples (75-100% female in most studies)
- **Notable findings:** Female-to-male ratios vary by disorder: AN (15.5:1), BN (3.2:1), BED (2.1:1) (Qian2021)

Socioeconomic Status:
- **Articles including SES data:** 5, 12
- **Limited reporting:** Most studies fail to systematically report SES data
- **Available data:** Pregnant women with EDs show 48.2% secondary education, 29.9% higher education (Sahu2025)

Race and Ethnicity:
- **Articles reporting race/ethnicity:** 1, 5, 13, 14
- **Major gap identified:** Predominantly White/Caucasian samples across Western studies
- **Geographic diversity:** Studies from Western Asia (Alfalahi2022), Iran (Hashemi2022), and migrant populations (Siddiqi2024) provide non-Western perspectives

Notable Demographic Gaps:
- Severe underrepresentation of males, ethnic minorities, and non-Western populations
- Limited SES reporting across most studies
- Age bias toward young adults with insufficient data on children and older adults

4. Study Comparison

Similarities in Design:
- **Systematic reviews/meta-analyses:** Articles 1-14 all employ systematic review methodology
- **Database searches:** Consistent use of PubMed, PsycINFO, Embase across studies
- **Quality assessment:** Most studies employ standardized quality assessment tools (JBI, Newcastle-Ottawa, Downs & Black)

Differences in Populations:
- **Geographic focus:** Regional (Articles 1, 6) vs. global (Articles 2, 3, 11) approaches
- **Age groups:** General population (Articles 1, 6, 11) vs. youth-specific (Articles 10, 14) vs. pregnancy-specific (Article 12)

- **Clinical vs. community samples:** Mixed approaches across studies

Convergent Findings:
- **High comorbidity rates:** Articles 2, 3, 4, 5, 7, 10, 14 consistently show elevated psychiatric comorbidity
- **Binge-purge risk pattern:** Articles 2, 3, 4, 7, 10, 14 consistently identify higher risk in binge-purge subtypes
- **Cultural protection:** Articles 1, 6, 13 suggest cultural factors may be protective in non-Western populations

Divergent Findings:
- **Assessment method effects:** Self-report screening shows higher prevalence than clinical interviews (Articles 1, 13)
- **Geographic variation:** Western vs. non-Western prevalence rates differ substantially (Articles 1, 6, 11, 13)

5. Article-Specific Information

Article 1 (Alfalahi2022):
- **Objective:** Determine eating disorder prevalence in Western Asia
- **Design:** Meta-analysis of 27 studies from 8 countries
- **Population:** 11,841 participants, mixed gender, Western Asian
- **Key Findings:** EAT-26/40: 22.07% (15.09-29.95%); Clinical interviews: AN 1.59%, BN 2.41%
- **Limitations:** High heterogeneity (I^2 > 90%), limited validation of screening tools

Article 2 (Amiri2023):
- **Objective:** Examine suicide-related behaviors in eating disorders
- **Design:** Meta-analysis of 52 studies
- **Population:** Predominantly female eating disorder patients
- **Key Findings:** NSSI 40% (33-46%), suicidal ideation 51% (41-62%), suicide attempts 22% (18-25%)
- **Subgroup Analysis:** BN shows higher rates than AN across all outcomes
- **Limitations:** High heterogeneity, limited male representation

Article 3 (Amiri2024):
- **Objective:** Assess alcohol and smoking prevalence in eating disorders
- **Design:** Meta-analysis of 42 studies
- **Population:** Eating disorder patients, predominantly female
- **Key Findings:** Alcohol use 26% (12-21%), smoking 30% (16-25%)
- **Subgroup Analysis:** BN highest (alcohol 28%, smoking 30%), AN lowest (alcohol 15%, smoking 13%)
- **Limitations:** High heterogeneity, geographic bias toward developed countries

Article 4 (Devoe2022):
- **Objective:** Examine impulse control disorders in eating disorders

- **Design:** Meta-analysis of 35 studies (9,646 participants)
- **Population:** 97.7% female, mean age 25.5 years
- **Key Findings:** Overall ICD/BA 22%, compulsive buying 19%, kleptomania 18%
- **Subgroup Analysis:** BN shows highest rates (24%), binge-purge behaviors associated with higher prevalence
- **Limitations:** Predominantly female samples, varied assessment methods

Article 5 (Drymonitou2024):
- **Objective:** Investigate eating disorder-psychosis comorbidity
- **Design:** Meta-analysis of 16 studies from 32 reviewed
- **Population:** Mixed clinical and community samples
- **Key Findings:** 7% (2-13%) comorbidity prevalence
- **Subgroup Analysis:** No significant differences between ED vs. psychosis populations as index conditions
- **Limitations:** High heterogeneity, limited diversity in samples

Article 6 (Hashemi2022):
- **Objective:** Determine eating disorder prevalence in Iran
- **Design:** Meta-analysis of 44 studies (61,088 participants)
- **Population:** 63.5% female, Iranian general population
- **Key Findings:** Point prevalence 22% (7-38%), lifetime prevalence 1% (1-2%)
- **Gender Analysis:** Similar rates between men (20%) and women (23%) for point prevalence
- **Limitations:** High heterogeneity, significant publication bias

Article 7 (Kirkpatrick2023):
- **Objective:** Examine NSSI prevalence across eating disorder diagnoses
- **Design:** Meta-analysis of 79 studies (32,334 participants)
- **Population:** Predominantly female eating disorder patients
- **Key Findings:** Overall NSSI 34.59% (30.49-38.81%)
- **Subgroup Analysis:** AN-BP 41.98%, BN 36.97%, AN-R 23.19%, BED 21.21%
- **Limitations:** Limited demographic diversity, varied NSSI definitions

Article 8 (Krug2025):
- **Objective:** Update mortality rates across eating disorders
- **Design:** Meta-analysis of 31 studies (262,982 patients)
- **Population:** Mixed gender, clinical eating disorder samples
- **Key Findings:** Overall SMR 3.39 (2.90-3.95), AN highest at 5.21 (4.10-6.62)
- **Moderator Analysis:** Older age at study onset and shorter follow-up predict higher AN mortality
- **Limitations:** Underrepresentation of certain ethnic groups

Article 9 (Low2020):

- **Objective:** Evaluate VR-enhanced CBT for binge-purging disorders
- **Design:** Meta-analysis of 6 RCTs (297 participants)
- **Population:** Bulimia nervosa and binge eating disorder patients
- **Key Findings:** VR-CBT superior for binge frequency (MD=0.29, 0.01-0.57) and situation-induced body dissatisfaction (SMD=0.72, 0.25-1.20)
- **Limitations:** Limited number of RCTs, moderate-high risk of bias

Article 10 (Meier2025):
- **Objective:** Examine NSSI in youth with eating disorders
- **Design:** Meta-analysis of 15 studies (3,311 participants)
- **Population:** 75.5-100% female, ages 9-19 years
- **Key Findings:** Lifetime NSSI 34.2% (27.5-41.7%)
- **Subgroup Analysis:** BN 53.2% vs. AN 23.3%; AN-BP 51.9% vs. AN-R 15.8%
- **Limitations:** Limited male representation, high heterogeneity

Article 11 (Qian2021):
- **Objective:** Update global eating disorder prevalence
- **Design:** Meta-analysis of 33 studies (315,877 participants)
- **Population:** General population, mixed gender
- **Key Findings:** Overall lifetime prevalence 1.69%, with BED most common at 1.53%
- **Subgroup Analysis:** Western countries 8.5× higher than Asian countries; DSM-5 shows higher prevalence than DSM-IV
- **Limitations:** Geographic bias, varied diagnostic criteria

Article 12 (Sahu2025):
- **Objective:** Examine eating disorders in pregnancy
- **Design:** Meta-analysis of 11 studies (2,369,520 pregnant women)
- **Population:** 100% female, pregnant women globally
- **Key Findings:** 4.3% (2-9%) prevalence, BED most common
- **Risk Factors:** Age <30 (61.6%), secondary education (48.2%), anxiety comorbidity (49.4%)
- **Limitations:** High heterogeneity, limited pregnancy-specific assessment tools

Article 13 (Siddiqi2024):
- **Objective:** Compare eating disorders in migrants vs. local populations
- **Design:** Meta-analysis of 6 studies, narrative synthesis of 4
- **Population:** International migrants and local comparison groups
- **Key Findings:** Migrants show 55% reduced odds (OR=0.45, 0.35-0.59) using diagnostic instruments
- **Subgroup Analysis:** Results vary significantly by assessment tool type
- **Limitations:** Limited number of studies, high heterogeneity

Article 14 (Sohn2023):
- **Objective:** Compare self-harm behaviors between eating disorder

and control populations
- **Design:** Meta-analysis of 32 studies
- **Population:** Mixed samples with eating disorders vs. healthy/psychiatric controls
- **Key Findings:** ED vs. HC: NSSI OR=6.85, suicidal ideation OR=3.63, suicide attempts OR=5.16
- **Subgroup Analysis:** BN shows highest suicide attempt risk (OR=5.93) among ED subtypes
- **Limitations:** High heterogeneity, predominantly female samples

This comprehensive cluster reveals eating disorders as complex conditions with substantial global prevalence variation, severe mortality risk, and extensive psychiatric comorbidity, particularly involving self-harm behaviors, substance use, and impulse control problems. The findings emphasize the critical need for culturally adapted assessment tools, comprehensive screening protocols, and integrated treatment approaches addressing multiple comorbid conditions.

SUMMARY FOR ARTICLE GROUP 14

Cluster Analysis Results: articles
Number of articles: 13
==
SYSTEMATIC UMBRELLA REVIEW CLUSTER ANALYSIS: MENTAL HEALTH COMORBIDITIES AND EPIDEMIOLOGY

1. Overall Cluster Label
Mental Health Comorbidity and Prevalence Studies

Subcategories Identified:
- **Neurodevelopmental Comorbidities** (Anderson2021, Micai2023, Kwon2024, Sharma2021)
- **Eating Disorder Comorbidities** (Cooney2024, Drakes2021, Mandelli2023)
- **Population Prevalence Studies** (Chan2023, Faria2025, Sacco2022, Solmi2022)
- **Familial Risk and Economic Burden** (Uher2023, Yadav2023)

2. Overall Findings Summary

Most Scientifically Relevant Findings

Transdiagnostic Risk Patterns:
- Uher2023 demonstrated that offspring of parents with mental disorders have 1.5-3.0 times higher risk of developing any mental disorder, with 37 significant transdiagnostic associations identified across 3.2 million offspring
- Sharma2021 found 69% (95% CI: 58-79%) of individuals with OCD have psychiatric comorbidity, establishing comorbidity as the rule rather

than exception

Neurodevelopmental Comorbidity Burden:
- Anderson2021 showed very preterm/very low birth weight individuals have dramatically elevated odds: ADHD OR=5.31 (95% CI: 3.10-9.35), ASD OR=10.5 (95% CI: 2.50-33.6)
- Micai2023 found autism has extensive comorbidities: developmental coordination disorder 87% (95% CI: 87-88%), ADHD 37% (95% CI: 28-46%), anxiety 35% (95% CI: 30-39%)
- Kwon2024 demonstrated epilepsy patients have elevated psychiatric risks: ASD OR=11.67 (95% CI: 6.35-21.41), ADHD OR=3.93 (95% CI: 3.81-4.18)

Eating Disorder Comorbidity Patterns:
- Drakes2021 found OCD prevalence in eating disorders: lifetime 13.9% (95% CI: 10.4-18.1), current 8.7% (95% CI: 5.8-11.8), with anorexia binge-purge subtype showing highest risk (22.2% lifetime)
- Cooney2024 showed PCOS increases eating disorder risk: any eating disorder OR=1.42 (95% CI: 1.18-1.71), binge eating disorder OR=2.09 (95% CI: 1.17-3.72)

Most Clinically Relevant Findings

Mortality Impact:
- Chan2023 revealed mental disorders reduce life expectancy by 14.66 years (95% CI: 13.88-15.98), with substance use disorders showing greatest impact (20.38 years YPLL)

Age-Related Patterns:
- Solmi2022 established critical timing: 34.6% of mental disorders onset before age 14, 48.4% before age 18, challenging the age-18 service division
- Sacco2022 found 15.5% (95% CI: 9.4-24.5%) prevalence of mental disorders in European youth, with anxiety disorders most common at 7.9%

Population Prevalence:
- Faria2025 found global eating disorder prevalence in youth of 5.23% (95% CI: 0.41-10.05%), with OSFED most common at 4.88%

Economic Impact:
- Yadav2023 quantified PCOS-related mental health costs at $3.15 billion annually in the US alone

3. Population Analysis

Gender Distribution
Articles reporting gender data: Anderson2021, Drakes2021, Mandelli2023, Faria2025, Micai2023, Sacco2022, Sharma2021
- **Female predominance:** Eating disorder studies (Drakes2021: 95.9%

female; Mandelli2023: 99-100% female)
- **Balanced representation:** Anderson2021, Sharma2021 (~51% female), Sacco2022 (varied by study)
- **Male predominance:** Micai2023 (only 20% female in autism studies)

Socioeconomic Status
Articles including SES data: None of the 13 articles provided detailed SES breakdowns, representing a significant demographic gap across the cluster.

Race and Ethnicity
Articles reporting race/ethnicity data: None provided detailed racial/ethnic breakdowns, though several noted geographic diversity (Chan2023: 24 countries; Uher2023: global scope; Sacco2022: 14 European countries).

Notable Demographic Gaps
- **Critical SES gap:** No studies systematically examined socioeconomic factors
- **Racial/ethnic representation gap:** Lack of diversity reporting across all studies
- **Geographic bias:** Predominance of high-income country data (Uher2023: only 1% from low/middle-income countries)
- **Gender imbalance:** Significant underrepresentation of females in autism research, males in eating disorder research

4. Study Comparison

Similarities in Design and Methodology
Meta-analytic approaches: All 13 studies used systematic review and meta-analysis methodology
Diagnostic criteria: Studies consistently used DSM/ICD criteria (Anderson2021, Drakes2021, Mandelli2023, Sharma2021, Sacco2022)
Random-effects modeling: Standard across studies due to expected heterogeneity
Quality assessment: Newcastle-Ottawa Scale or similar tools used consistently

Differences in Populations and Settings
Age ranges:
- **Pediatric focus:** Anderson2021 (preterm birth outcomes), Faria2025 (children/youth), Sacco2022 (European youth)
- **Adult focus:** Chan2023 (mortality), Cooney2024 (reproductive-age women), Yadav2023 (women with PCOS)
- **Lifespan approach:** Sharma2021, Solmi2022, Uher2023

Clinical vs. Population samples:
- **Clinical samples:** Drakes2021, Mandelli2023, Sharma2021 showed higher comorbidity rates
- **Population-based:** Sacco2022, Faria2025 provided community

prevalence estimates
- **Mixed approaches:** Anderson2021, Chan2023, Uher2023 combined multiple study types

Convergent vs. Divergent Findings

Convergent findings:
- **High comorbidity rates:** Consistent across Anderson2021, Micai2023, Kwon2024, Sharma2021
- **Gender patterns:** Males higher for neurodevelopmental disorders (Anderson2021, Micai2023, Sacco2022), females higher for eating/mood disorders (Drakes2021, Mandelli2023)
- **Age effects:** Earlier onset for neurodevelopmental conditions, later for mood disorders (Solmi2022, Sharma2021, Sacco2022)

Divergent findings:
- **Heterogeneity levels:** Extremely high (I^2>90%) in Faria2025, Sacco2022 vs. moderate in Anderson2021
- **Study design effects:** Registry studies showed 3-10 times lower risks than family studies (Uher2023)
- **Geographic variations:** Substantial differences across regions (Chan2023, Sacco2022)

Similarities and Differences in Conclusions

Similar conclusions:
- Mental health comorbidities are pervasive across conditions
- Early identification and intervention are critical
- Current service models inadequately address comorbidity burden
- Significant unmet mental health needs exist globally

Different conclusions:
- **Service implications:** Solmi2022 challenges age-18 service divisions; others focus on condition-specific screening
- **Prevention targets:** Uher2023 emphasizes familial risk; Anderson2021 focuses on perinatal factors
- **Economic priorities:** Yadav2023 quantifies costs; others focus on clinical burden

5. Article-Specific Information

Anderson2021
- **Research question:** Psychiatric disorder prevalence in very preterm/very low birth weight individuals
- **Design:** Individual participant data meta-analysis, 10 cohorts, 3,028 participants
- **Population:** VP/VLBW individuals vs. term controls across 8 countries
- **Key findings:** ADHD OR=5.31 (3.10-9.35), ASD OR=10.5 (2.50-33.6), anxiety OR=1.81 (1.26-2.58)

- **Subgroup analyses:** Age and sex interactions confirmed across developmental periods
- **Limitations:** Limited to structured diagnostic assessments, potential selection bias

Chan2023
- **Research question:** Life expectancy and years of potential life lost across mental disorders
- **Design:** Systematic review and meta-analysis, 109 studies, 12.2 million patients
- **Population:** Global sample across 24 countries, mixed ages and disorders
- **Key findings:** 14.66 years YPLL (13.88–15.98), substance use disorders highest at 20.38 years
- **Subgroup analyses:** Gender, geographic, and temporal variations examined
- **Limitations:** High heterogeneity, limited cause-of-death data quality

Cooney2024
- **Research question:** Eating disorders and disordered eating in PCOS vs. controls
- **Design:** Systematic review and meta-analysis, 20 studies, 167,079 women
- **Population:** Women with PCOS vs. controls across multiple countries
- **Key findings:** Any eating disorder OR=1.42 (1.18–1.71), binge eating OR=2.09 (1.17–3.72)
- **Subgroup analyses:** BMI stratification showed consistent effects across weight categories
- **Limitations:** Cross-sectional design, heterogeneous PCOS diagnostic criteria

Drakes2021
- **Research question:** OCD comorbidity prevalence in eating disorder populations
- **Design:** Bayesian multi-level meta-analysis, 59 studies, 35 lifetime and 42 current estimates
- **Population:** 95.9% female eating disorder samples across multiple countries
- **Key findings:** Lifetime OCD 13.9% (10.4–18.1), current 8.7% (5.8–11.8)
- **Subgroup analyses:** ANBP subtype highest risk (22.2% lifetime), clinical samples higher than community
- **Limitations:** Publication bias detected, limited male representation

Faria2025
- **Research question:** Global prevalence of eating disorders in children and young people

- **Design:** Systematic review and meta-analysis, 15 studies, 68,154 participants
- **Population:** Children and adolescents from 11 countries
- **Key findings:** Overall ED prevalence 5.23% (0.41-10.05), OSFED most common at 4.88%
- **Subgroup analyses:** Geographic location major predictor of heterogeneity (R^2=81.46%)
- **Limitations:** Extremely high heterogeneity (I^2>99%), limited geographic representation

Kwon2024
- **Research question:** Psychiatric comorbidities in persons with epilepsy
- **Design:** Systematic review and meta-analysis, 27 studies, 565,333 PWE
- **Population:** Epilepsy patients vs. 13.2 million controls
- **Key findings:** ASD OR=11.67 (6.35-21.41), alcohol dependence OR=4.94 (3.51-6.96)
- **Subgroup analyses:** Consistent across diagnostic methods and age groups
- **Limitations:** Limited demographic data, heterogeneous epilepsy populations

Mandelli2023
- **Research question:** OCD comorbidity rates across eating disorder subtypes
- **Design:** Binary random effects meta-analysis, 31 studies
- **Population:** 99-100% female eating disorder samples
- **Key findings:** Lifetime OCD 22.9% (16.3-28.9), current 14.4% (12.6-17.6)
- **Subgroup analyses:** AN higher current rates than BN (OR=2.14), longitudinal studies higher estimates
- **Limitations:** High heterogeneity (I^2=89%), publication bias detected

Micai2023
- **Research question:** Co-occurring conditions prevalence in autism across lifespan
- **Design:** Systematic review and meta-analysis, 340 studies, 592,169 participants
- **Population:** Autistic individuals across 68 different co-occurring conditions
- **Key findings:** Developmental coordination disorder 87%, sleep problems 43%, ADHD 37%
- **Subgroup analyses:** Age differences (children higher ADHD, adults higher depression)
- **Limitations:** Gender imbalance (20% female), high heterogeneity (I^2>90%)

Sacco2022

- **Research question:** Mental disorder prevalence in European children and adolescents
- **Design:** Systematic review and meta-analysis, 17 studies, 50,605 participants
- **Population:** Children/adolescents from 14 European countries
- **Key findings:** Overall prevalence 15.5% (9.4–24.5%), anxiety disorders highest at 7.9%
- **Subgroup analyses:** Gender and age differences, geographic variations across Europe
- **Limitations:** High heterogeneity (I^2>94%), limited substance use disorder data

Sharma2021
- **Research question:** Comorbidities in OCD across the lifespan
- **Design:** Systematic review and meta-analysis, 81 studies, 15,767 individuals
- **Population:** OCD patients across pediatric and adult samples globally
- **Key findings:** Any psychiatric comorbidity 69% (58–79%), mood disorders 37% (29–46%)
- **Subgroup analyses:** Age effects (anxiety higher in children, mood higher in adults)
- **Limitations:** High heterogeneity, predominantly clinical samples

Solmi2022
- **Research question:** Age at onset patterns across all mental disorders globally
- **Design:** Systematic review and meta-analysis, 192 studies, 708,561 individuals
- **Population:** General population across 54 countries, all mental disorder categories
- **Key findings:** 34.6% onset before age 14, peak age 14.5 years, median 18 years
- **Subgroup analyses:** Disorder-specific patterns from neurodevelopmental (peak=5.5) to mood (peak=20.5)
- **Limitations:** Heterogeneous data requiring novel statistical approaches

Uher2023
- **Research question:** Transdiagnostic mental health risks in offspring of affected parents
- **Design:** Systematic review and meta-analysis, 211 studies, 23.6 million participants
- **Population:** Offspring of parents with mental disorders vs. controls
- **Key findings:** Risk ratios 1.5–3.0 for any disorder, 2.2–8.4 for same disorder
- **Subgroup analyses:** 37 transdiagnostic associations, age-dependent cumulative incidence
- **Limitations:** High heterogeneity, 96% studies at high risk of

bias

Yadav2023
- **Research question:** Economic burden of mental health disorders in PCOS
- **Design:** Systematic review and meta-analysis, 25 studies
- **Population:** Women with PCOS across multiple countries
- **Key findings:** Anxiety PR=1.31 (1.21-1.41), depression PR=1.54 (1.33-1.78)
- **Economic analyses:** Total excess costs $3.15 billion annually in US
- **Limitations:** Limited to direct healthcare costs, US-focused economic modeling

SUMMARY FOR ARTICLE GROUP 15

Cluster Analysis Results: articles
Number of articles: 11
==

SYSTEMATIC UMBRELLA REVIEW CLUSTER ANALYSIS: EATING DISORDERS AND COMORBID CONDITIONS

1. Overall Cluster Label
Eating Disorders Comorbidity and Behavioral Addictions

Subcategories Identified:
- **Substance Use Comorbidities** (alcohol, tobacco)
- **Behavioral Addictions** (food addiction, exercise addiction)
- **Impulse Control Disorders** (kleptomania, compulsive buying)
- **Psychological Factors** (attachment patterns)
- **Social Determinants** (food insecurity)
- **Clinical Transitions** (diagnostic instability, post-surgical outcomes)

2. Overall Findings Summary

Primary Comorbidity Prevalence Findings

Substance Use:
- **Alcohol use in eating disorders**: 26% overall prevalence (95% CI: 19-31%) with differential patterns by ED subtype - BN: 38%, BED: 27%, AN: 24% (Amiri2024)
- **Lifetime alcohol use disorder in binge eating**: 19.9% prevalence (95% CI: 13.7-27.9%) with 59% increased risk vs. controls (RR=1.59, 95% CI: 1.41-1.79) (Bogusz2020)
- **Tobacco smoking**: AN: 23.4% (95% CI: 18.29-28.80%), BN: 19.3%, BED: 11.9% (95% CI: 9.52-14.45%) (Flores2025)

Behavioral Addictions:
- **Food addiction**: 20% global prevalence (95% CI: 18–21%), rising to 55% in binge eating clinical populations (95% CI: 34–75%) (Praxedes2022)
- **Exercise addiction**: 3.71-fold increased odds in eating disorders (95% CI: 2.00-6.89) (Trott2021)

Impulse Control Disorders:
- **Overall ICD/BA prevalence**: 22% (95% CI: 15–30%) with compulsive buying (19%, 95% CI: 13–25%) and kleptomania (18%, 95% CI: 9–29%) most common (Devoe2022)

Subgroup and Moderation Analyses

ED Subtype Patterns:
- Binge-purge subtypes (BN, AN-BP) consistently show higher comorbidity rates than restrictive subtypes across multiple conditions
- BN shows highest rates for most substances and behavioral addictions
- BED shows intermediate rates between BN and AN-R for most comorbidities

Gender Effects:
- Higher female representation associated with lower smoking rates in BED populations (Flores2025)
- AUD prevalence inversely related to proportion of women in samples (Bogusz2020)

Clinical vs. Community Samples:
- Clinical samples show consistently higher comorbidity rates (e.g., food addiction: 31% clinical vs. 14% non-clinical) (Praxedes2022)
- Community samples show higher AUD prevalence than clinical samples (27.45% vs. 14.45%) (Bogusz2020)

Social and Psychological Factors

Food Insecurity:
- Adults with food insecurity show 66% higher odds of binge eating (OR=1.66, 95% CI: 1.42–1.93) and 170% higher odds of BED (OR=2.70, 95% CI: 1.47–4.96) (Abene2023)

Attachment Patterns:
- Large effect sizes for attachment insecurity across all ED types: security (d=0.91–1.02), avoidance (d=0.81–0.87), anxiety (d=0.83–0.95) (Jewell2023)

Clinical Transitions and Outcomes

Diagnostic Instability:
- 31.73% of AN-R patients transition to binge-purge behaviors (95% CI: 22.47–41.01%) (Serra2021)

Post-Surgical Outcomes:
- 7.83% prevalence of eating disorders post-bariatric surgery (95% CI: 4.30-11.37%) with BED most common at 3.81% (Taba2021)

3. Population Analysis

Gender Distribution
- **Articles with gender data**: All 11 articles report gender information
- **Predominant pattern**: Heavily female-skewed samples (70-100% female in most studies)
- **Specific distributions**: Jewell2023, Serra2021 (exclusively female); Devoe2022 (97.7% female); Taba2021 (71% female); Trott2021 (70.6% female)

Socioeconomic Status
- **Articles with SES data**: Very limited reporting across all studies
- **Notable gaps**: Only Abene2023 reports federal benefits data (20.8% of studies); Jewell2023 notes bias toward higher SES
- **Major limitation**: Systematic underreporting of SES across the field

Race and Ethnicity
- **Articles with race/ethnicity data**: Abene2023 (diverse samples), Bogusz2020 (limited reporting), Jewell2023 (predominantly White)
- **Geographic bias**: Studies predominantly from Western, high-income countries
- **Major gap**: Significant underrepresentation of non-White populations across all studies

Notable Demographic Biases
- Systematic exclusion of males limits generalizability
- Geographic concentration in Western countries
- SES reporting inadequate for understanding social determinants
- Age bias toward young adults in most samples

4. Study Comparison

Methodological Similarities
- **Shared approaches**: All studies used systematic review with meta-analysis methodology following PRISMA guidelines
- **Statistical methods**: Random-effects models predominant across studies (Abene2023, Amiri2024, Bogusz2020, Devoe2022, Flores2025, Jewell2023, Praxedes2022, Serra2021, Taba2021, Trott2021, diGiacomi2022)
- **Quality assessment**: Most employed standardized quality assessment tools (Newcastle-Ottawa Scale, Downs and Black)

Population and Setting Differences
- **Sample sizes**: Ranged from 614 (Serra2021) to 269,050 participants (Praxedes2022)
- **Clinical focus**: Clinical samples (Serra2021, Taba2021) vs. mixed populations (Praxedes2022, Abene2023)
- **Geographic scope**: Global (Praxedes2022, Flores2025) vs. regional focus (Abene2023 - US only)

Convergent Findings
- **Consistent elevation**: All studies demonstrate elevated comorbidity rates in ED populations vs. controls
- **Binge-purge pattern**: Multiple studies (Amiri2024, Bogusz2020, Devoe2022, Flores2025) show highest comorbidity in binge-purge subtypes
- **Clinical significance**: All studies emphasize need for integrated screening and treatment

Divergent Findings
- **Community vs. clinical samples**: Bogusz2020 found higher AUD in community samples while Praxedes2022 found higher food addiction in clinical samples
- **Gender effects**: Variable across conditions (protective for some, risk factor for others)
- **Heterogeneity**: High statistical heterogeneity (I^2 >80%) in most analyses suggests substantial between-study variation

Conclusion Similarities and Differences
- **Shared conclusions**: All emphasize clinical importance of comorbidity screening and integrated treatment approaches
- **Differing emphasis**: Some focus on etiological implications (Abene2023, Jewell2023) while others emphasize clinical management (Taba2021, Serra2021)
- **Treatment implications**: Convergent recommendations for routine screening but different specific intervention suggestions

5. Article-Specific Information

Article 1: Abene2023
- **Research question**: Association between food insecurity and binge eating behaviors
- **Design**: Systematic review and meta-analysis of 24 articles (20 studies)
- **Population**: Mixed socioeconomic backgrounds, 87.5% studies ≥50% female, diverse racial composition
- **Primary findings**: Food insecure adults had 66% higher odds of binge eating (OR=1.66, 95% CI: 1.42-1.93) and 170% higher odds of BED (OR=2.70, 95% CI: 1.47-4.96)
- **Limitations**: US-focused studies, insufficient adolescent data, cross-sectional designs limit causality

Article 2: Amiri2024
- **Research question**: Prevalence of alcohol use and smoking across eating disorder subtypes
- **Design**: Meta-analysis of 42 studies
- **Population**: Predominantly female, international scope
- **Primary findings**: Overall alcohol use 26% (95% CI: 19-31%), smoking 29% (95% CI: 25-34%) with highest rates in BN
- **Subgroup analyses**: BN showed highest prevalence for both substances vs. AN and BED
- **Limitations**: High heterogeneity (I^2>88%), inconsistent demographic reporting

Article 3: Bogusz2020
- **Research question**: Lifetime prevalence of alcohol use disorder in binge eating disorder
- **Design**: Meta-analysis of 18 studies (69,233 individuals)
- **Population**: Mixed gender (31-100% women), limited race/ethnicity reporting
- **Primary findings**: 19.9% lifetime AUD prevalence (95% CI: 13.7-27.9%), RR=1.59 vs. controls (95% CI: 1.41-1.79)
- **Moderation**: Higher AUD in community vs. clinical samples (27.45% vs. 14.45%, p=0.041)
- **Limitations**: High heterogeneity (I^2=96.6%), varying diagnostic criteria

Article 4: Devoe2022
- **Research question**: Prevalence of impulse control disorders and behavioral addictions in eating disorders
- **Design**: Meta-analysis of 35 studies (9,646 participants)
- **Population**: 97.7% female, mean age 25.5 years
- **Primary findings**: Overall ICD/BA prevalence 22% (95% CI: 15-30%), compulsive buying 19% (95% CI: 13-25%), kleptomania 18% (95% CI: 9-29%)
- **Subgroup analyses**: BN and AN-BP showed higher rates than AN-R across most ICDs
- **Limitations**: High heterogeneity (I^2>90%), male underrepresentation, quality issues in included studies

Article 5: Flores2025
- **Research question**: Current tobacco smoking prevalence across eating disorder types
- **Design**: Meta-analysis of 30 studies (15,706 individuals)
- **Population**: 76.3% female, geographically diverse
- **Primary findings**: AN 23.4% (95% CI: 18.29-28.80%), BN 19.3%, BED 11.9% (95% CI: 9.52-14.45%)
- **Moderation**: For BED, higher female proportion and non-DSM tools associated with lower prevalence
- **Limitations**: High heterogeneity (I^2=77-96%), publication bias detected for BN

Article 6: Jewell2023
- **Research question**: Attachment patterns in eating disorders vs. community controls
- **Design**: Meta-analysis of 35 studies
- **Population**: Predominantly female, White, higher SES, Western countries
- **Primary findings**: Large effect sizes for attachment insecurity - security d=0.91-1.02, avoidance d=0.81-0.87, anxiety d=0.83-0.95
- **Moderation**: Only assessor blinding significant moderator for interview studies
- **Limitations**: Demographic homogeneity, high unexplained heterogeneity, potential confounding by general psychopathology

Article 7: Praxedes2022
- **Research question**: Global prevalence of food addiction across populations
- **Design**: Meta-analysis of 272 studies (269,050 participants)
- **Population**: Predominantly female (21 studies exclusively female)
- **Primary findings**: 20% overall prevalence (95% CI: 18-21%), 55% in binge eating clinical populations (95% CI: 34-75%)
- **Subgroup analyses**: Clinical samples 31% vs. non-clinical 14%, increasing with weight status
- **Limitations**: Predominantly female samples, cross-sectional designs, measurement tool variations

Article 8: Serra2021
- **Research question**: Transition from restrictive AN to binge-purge behaviors
- **Design**: Meta-analysis of 12 longitudinal studies (614 patients)
- **Population**: All women
- **Primary findings**: 31.73% transition rate (95% CI: 22.47-41.01%), 31.81% remission rate (95% CI: 19.85-56.74%)
- **Risk factors**: Higher premorbid BMI, depression, hostile family attitudes, parental criticism
- **Limitations**: Small sample size, limited to women, predominantly Western countries

Article 9: Taba2021
- **Research question**: Eating disorder development following bariatric surgery
- **Design**: Meta-analysis of 14 studies (7 in meta-analysis, 5,774 participants)
- **Population**: 71% women, 29% men
- **Primary findings**: 7.83% overall ED prevalence (95% CI: 4.30-11.37%), BED most common at 3.81%
- **Subgroup analyses**: Variable by surgery type (RYGB 3.33-7.58%, AGB 3.39-17.24%)
- **Limitations**: High heterogeneity (I^2=85.6%), methodological limitations in primary studies, lack of standardized assessment

Article 10: Trott2021
- **Research question**: Exercise addiction prevalence in eating disorders vs. controls
- **Design**: Meta-analysis of 9 studies (2,140 participants)
- **Population**: 70.6% female overall
- **Primary findings**: OR=3.71 (95% CI: 2.00-6.89) for exercise addiction in ED vs. non-ED populations
- **Subgroup analyses**: Varying ORs by measurement tool (2.4-6.9 range)
- **Limitations**: High heterogeneity (I^2=81%), limited number of studies, measurement tool variations

Article 11: diGiacomi2022
- **Research question**: Food addiction prevalence across different eating disorders
- **Design**: Meta-analysis of 6 studies (2,476 participants)
- **Population**: Gender not consistently reported
- **Primary findings**: BED vs. obese patients OR=5.72 (95% CI: 3.25-10.09), vs. general population OR=55.41 (95% CI: 8.16-376.10)
- **Subgroup analyses**: BN showed higher food addiction than BED (OR=0.85 for BED vs. BN), BED higher than AN-R (OR=8.75, 95% CI: 1.08-70.70)
- **Limitations**: Small number of studies, heterogeneous populations, limited demographic data

SUMMARY FOR ARTICLE GROUP 16

Cluster Analysis Results: articles
Number of articles: 10

==

SYSTEMATIC UMBRELLA REVIEW CLUSTER SYNTHESIS

1. Overall Cluster Label
Eating Disorders Treatment Outcomes Meta-Analyses

Subcategories Identified:
- Treatment effectiveness and modalities (5 articles)
- Cognitive and behavioral characteristics (3 articles)
- Long-term outcomes and prognosis (2 articles)

2. Overall Findings Summary

Treatment Effectiveness Findings

Psychological Interventions: Hilbert2020 demonstrated that psychotherapy and self-help treatments maintain significant effectiveness for BED up to 12 months (abstinence rates 46-52% and 45-53% respectively), while Raisi2023 found large effect sizes for exercise interventions in BED (SMD = -0.94, 95% CI: -1.46 to -0.31).

Weight Loss Interventions: Forman2023 showed that individuals with BED achieved substantial weight loss in behavioral weight loss interventions (8.1 kg) but 1.4 kg less than non-BED individuals, with 50% higher dropout odds (OR=1.50, 95% CI: 1.14-1.98). Conversely, Tsompanaki2025 found weight loss interventions improved disordered eating symptoms (-1.47 SMD, 95% CI: -1.67, -1.27), with greater improvements in those with baseline eating disorders (-2.35 SMD, 95% CI: -2.78, -1.91).

Specialized Treatments: Atti2021 demonstrated that compulsory eating disorder treatment achieved similar outcomes to voluntary treatment despite patients having lower baseline BMI (mean difference = -0.57 kg/m^2, 95% CI: -0.22 to -0.91) and longer hospitalizations.

Cognitive and Behavioral Characteristics

Cognitive Functioning: Keegan2021 found that bulimia nervosa patients showed cognitive inefficiencies comparable to anorexia nervosa in central coherence (BN: g = -0.70, 95% CI: -1.14, -0.25; AN: g = -0.53, 95% CI: -0.80, -0.27) and set-shifting (BN: g = -0.55, 95% CI: -0.81, -0.29; AN: g = -0.38, 95% CI: -0.50, -0.26).

Binge Episode Characteristics: Mourilhe2021 revealed that binge episodes involve extremely large caloric intake (BN: 3070 kcal in laboratory studies, 1789 kcal in clinical studies; BED: ~2000 kcal), with positive correlation between caloric intake and depression severity (β = 55.5; p = 0.019).

Long-term Outcomes and Prognostic Factors

Duration Effects: Radunz2020 found no significant association between eating disorder duration and treatment outcomes (r = .05, 95% CI: -.03 to .13), challenging the clinical utility of "severe and enduring" classifications.

Behavioral Trajectories: Romano2020 demonstrated significant long-term improvements in most disordered eating behaviors over 10+ years, with 20-75% risk reductions for compensatory behaviors, though clinical samples showed better trajectories than community samples.

Surgical Outcomes: Kops2020 found no significant differences in weight loss outcomes between bariatric surgery patients with and without pre-operative BED across multiple timepoints (6-60 months).

3. Population Analysis

Gender Distribution:
- **Predominantly female samples:** Hilbert2020 (90%), Romano2020 (87.7%), Tsompanaki2025 (82%), Kops2020 (80.3%)

- **Exclusively female:** Raisi2023 (100%)
- **Mixed gender with high female representation:** Atti2021 (87.9–100%), Keegan2021, Radunz2020 (89.2–100%)
- **Gender not systematically reported:** Forman2023, Mourilhe2021 (only 4 of 43 studies included males)

Race/Ethnicity:
- **Reported with diverse representation:** Hilbert2020 (49–89% Caucasian, 4–35% African American, 4–20% Hispanic), Romano2020 (20.5% non-White, 5.6% Hispanic/Latinx), Tsompanaki2025 (57% White)
- **Predominantly White where reported:** Radunz2020 (75–99% White), Keegan2021
- **Not systematically reported:** Atti2021, Forman2023, Kops2020, Mourilhe2021, Raisi2023

Socioeconomic Status:
- **Largely middle-to-upper-middle class where reported:** Keegan2021
- **Not systematically reported across studies:** All other articles

Notable Demographic Gaps:
- Significant underrepresentation of male participants across all studies
- Limited racial/ethnic diversity reporting in half the studies
- Minimal socioeconomic status documentation
- Age ranges vary widely but systematic age-related analyses limited

4. Study Comparison

Similarities in Design and Methodology:
- **Meta-analytic approach:** All 10 articles employed systematic review and meta-analysis methodologies
- **Random-effects modeling:** Used consistently across studies (Atti2021, Forman2023, Hilbert2020, Keegan2021, Kops2020, Mourilhe2021, Radunz2020, Romano2020, Tsompanaki2025)
- **PRISMA guidelines:** Followed by multiple studies (Atti2021, Mourilhe2021, others)
- **Quality assessment:** Newcastle-Ottawa Scale (Atti2021), risk of bias assessments (multiple studies)

Differences in Populations and Settings:
- **Clinical vs. community samples:** Hilbert2020, Romano2020, Tsompanaki2025 included both; others focused primarily on clinical populations
- **Diagnostic focus:** Specialized focus on specific disorders (Raisi2023-BED only, Atti2021-AN/BN) vs. transdiagnostic approaches (Keegan2021, Romano2020)
- **Treatment settings:** Inpatient focus (Atti2021), outpatient emphasis (most others), surgical settings (Kops2020)

Convergent Findings:

- **Treatment effectiveness:** Multiple studies (Hilbert2020, Raisi2023, Tsompanaki2025) support various interventions' efficacy
- **Cognitive similarities:** Keegan2021 and broader literature suggest transdiagnostic cognitive features
- **Long-term improvement:** Romano2020 and Hilbert2020 both demonstrate sustained benefits over time
- **Weight-related outcomes:** Convergent evidence that weight concerns don't necessarily predict poor outcomes (Kops2020, Tsompanaki2025)

Divergent Findings:
- **Weight loss intervention effects:** Forman2023 shows reduced effectiveness in BED patients vs. Tsompanaki2025 showing improved disordered eating symptoms
- **Clinical vs. laboratory findings:** Mourilhe2021 demonstrates significant methodological differences in binge episode assessment
- **Duration effects:** Radunz2020 challenges traditional prognostic assumptions about illness duration

Conclusion Similarities:
- All studies emphasize the complexity of eating disorders and need for individualized treatment
- Multiple studies highlight gaps in long-term follow-up research
- Consistent calls for improved standardization of assessment measures
- Recognition of the importance of addressing comorbid conditions

Conclusion Differences:
- **Treatment recommendations:** Range from cognitive remediation (Keegan2021) to exercise interventions (Raisi2023) to challenging exclusion criteria (multiple studies)
- **Clinical practice implications:** Some challenge existing practices (Atti2021, Radunz2020) while others support current approaches
- **Research priorities:** Vary from methodological improvements to specific population studies

5. Article-Specific Information

Article 1: Atti2021
- **Research Question:** Compare outcomes between compulsory vs. voluntary eating disorder treatment
- **Design:** Meta-analysis of 9 retrospective studies (242 compulsory vs. 738 voluntary patients)
- **Population:** 87.9-100% female, primarily AN/BN patients
- **Key Findings:** Similar outcomes despite compulsory patients having lower baseline BMI (-0.57 kg/m^2, 95% CI: -0.22 to -0.91) and longer hospitalizations
- **Limitations:** Retrospective studies, limited to hospitalized patients, heterogeneous legal frameworks

Article 2: Forman2023
- **Research Question:** Compare behavioral weight loss intervention outcomes in BED vs. non-BED individuals
- **Design:** Meta-analysis of 30 manuscripts (65 treatment groups, n=7,864)
- **Population:** BED n=1,519, non-BED n=6,345
- **Key Findings:** BED group lost less weight (8.1 vs. 9.4 kg) with higher dropout rates (OR=1.50, 95% CI: 1.14-1.98)
- **Subgroup Analysis:** Difference more pronounced without meal replacements (2.9 kg difference)
- **Limitations:** Heterogeneous interventions, limited long-term follow-up

Article 3: Hilbert2020
- **Research Question:** Long-term effectiveness of BED treatments across modalities
- **Design:** Meta-analysis of 114 studies (8,862 individuals)
- **Population:** 90% female, diverse racial representation (49-89% Caucasian)
- **Key Findings:** Psychotherapy and self-help maintained effectiveness up to 12 months (abstinence 45-53%)
- **Subgroup Analysis:** Limited long-term data beyond 12 months, particularly for pharmacotherapy
- **Limitations:** Within-group analyses only, high heterogeneity, limited high-quality studies (4%)

Article 4: Keegan2021
- **Research Question:** Compare cognitive functioning between eating disorder subtypes
- **Design:** Meta-analysis of central coherence (16 studies, 1,112 participants) and set-shifting (38 studies, 3,505 participants)
- **Population:** Primarily female, predominantly White, middle-to-upper-middle class
- **Key Findings:** AN and BN showed similar cognitive inefficiencies; BED did not show significant deficits
- **Moderation Analysis:** No significant differences between AN and BN effect sizes
- **Limitations:** Limited BED studies, potential publication bias, cross-sectional design

Article 5: Kops2020
- **Research Question:** Impact of preoperative BED on bariatric surgery weight loss outcomes
- **Design:** Meta-analysis of 19 studies (3,223 participants)
- **Population:** 80.25% female, median age 41 years
- **Key Findings:** No significant differences in weight loss between BED and non-BED groups across all timepoints (6-60 months)
- **Subgroup Analysis:** Results consistent across surgical types and assessment methods
- **Limitations:** High heterogeneity, variable BED assessment

methods, limited long-term follow-up

Article 6: Mourilhe2021
- **Research Question:** Characterize binge eating episodes across eating disorders
- **Design:** Meta-analysis of 43 studies examining caloric intake, duration, and psychopathology associations
- **Population:** Predominantly female (only 4 studies included males), limited ethnic diversity reporting
- **Key Findings:** Laboratory studies showed higher caloric intake than clinical studies (BN: 3070 vs. 1789 kcal)
- **Moderation Analysis:** Positive correlation between caloric intake and depression ($\beta = 55.5$; $p = 0.019$)
- **Limitations:** Methodological heterogeneity, limited male representation, temporal gaps in research

Article 7: Radunz2020
- **Research Question:** Relationship between eating disorder duration and treatment outcomes
- **Design:** Meta-analysis of 31 studies (24 with extractable data, 2,349 participants)
- **Population:** Mixed gender (89.2-100% female), predominantly White (75-99%)
- **Key Findings:** No significant association between duration and outcomes ($r = .05$, 95% CI: -.03 to .13)
- **Subgroup Analysis:** Results consistent across diagnoses and outcome types
- **Limitations:** High heterogeneity, variable outcome measures, potential confounding variables

Article 8: Raisi2023
- **Research Question:** Effectiveness of physical exercise interventions for BED
- **Design:** Bayesian meta-analysis of 5 RCTs (264 female participants)
- **Population:** 100% female
- **Key Findings:** Large effect size favoring exercise (SMD = -0.94, 95% CI: -1.46 to -0.31)
- **Bayesian Analysis:** 93.7% posterior probability for random-effect model, 15x greater probability of non-zero effect
- **Limitations:** Small number of studies, exclusively female samples, short-term follow-up

Article 9: Romano2020
- **Research Question:** Long-term changes in disordered eating behaviors over 10+ years
- **Design:** Meta-analysis of 17 studies (26 subsamples, baseline N=31-9,031)
- **Population:** 87.72% female, 20.51% non-White, 5.63% Hispanic/Latinx

- **Key Findings:** Significant risk reductions for most compensatory behaviors (20–75% lower risk)
- **Moderation Analysis:** Clinical samples showed better trajectories than community samples; younger age associated with higher risk
- **Limitations:** Variable follow-up periods, heterogeneous samples, limited standardized measures

Article 10: Tsompanaki2025
- **Research Question:** Impact of weight loss interventions on disordered eating symptoms
- **Design:** Meta-analysis of 38 studies (66 arms, 3,364 participants)
- **Population:** 82% female, 57% White ethnicity
- **Key Findings:** Weight loss interventions improved disordered eating symptoms (–1.47 SMD, 95% CI: –1.67, –1.27)
- **Subgroup Analysis:** Greater improvement in those with baseline eating disorders (–2.35 SMD, 95% CI: –2.78, –1.91)
- **Limitations:** High heterogeneity, variable intervention types, median 4–month follow-up
