# Supplementary material for: An Artificial Intelligence-Assisted Umbrella Review of Eating Disorder Meta-Analyses (2020–2025): Mapping Treatment Efficacy, Comorbidity, and Persistent Gaps in Sample Diversity
Source: Nutrients. 2026 Nov 3;18(21):3650. doi: 10.3390/nu18213650 (PMC13765187; doi:10.3390/nu18213650)
Supplement: Supplementary file 1 [file nutrients-18-03650-s001.zip › Phase2_summaries.pdf]

SUMMARY FOR CLUSTER 1

This cluster of 26 articles reveals consistent evidence that eating disorders involve profound neurocognitive dysfunction across multiple domains, with the most robust findings including moderate-to-large executive function deficits (particularly cognitive flexibility and inhibitory control), widespread gray matter reductions most pronounced in anorexia nervosa (Cohen's d up to −0.95; Walton2022), and severe interoceptive impairments across all eating disorder subtypes with large effect sizes (AN d=1.57, BN d=2.61, BED d=1.96; Cobbaert2024). Neuroimaging meta-analyses consistently identify limbic-prefrontal circuit alterations with distinct patterns between disorders—anorexia showing reward system hypoactivation while bulimia demonstrates hyperactivation—and evidence suggests these deficits are partially reversible with treatment, indicating both state and trait components. However, this research is severely limited by systematic underrepresentation of males, ethnic minorities, and non-Western populations, highlighting an urgent need for more diverse samples to establish the generalizability of these neurocognitive mechanisms and inform equitable treatment approaches.

SUMMARY FOR CLUSTER 2

This comprehensive synthesis of eating disorder treatment research reveals several robust findings with important clinical implications. **Cognitive Behavioral Therapy demonstrates consistently strong effectiveness across diverse populations and settings, with large effect sizes ranging from 0.88 in controlled trials to 1.12 in routine clinical care, achieving 36% abstinence rates compared to 10% in controls** (Cuijpers2024, Öst2023). **Family-based treatments show superior weight outcomes compared to individual therapy (g = 0.46) for adolescents, with separated parent-focused approaches outperforming conjoint formats** (Austin2024). Digital interventions emerge as a promising modality, demonstrating moderate effectiveness (g = 0.58) with self-monitoring identified as a critical behavior change technique present in all effective digital interventions (Haderlein2022, Thomas2024). However, the research base suffers from significant demographic limitations, with 90-100% female samples and predominantly White, Western populations across most studies, severely limiting generalizability. Despite treatment advances, substantial proportions of patients remain unremitted even with evidence-based interventions, highlighting the need for continued innovation in treatment approaches and the critical importance of developing more inclusive research that represents diverse gender, racial, ethnic, and

socioeconomic populations.

SUMMARY FOR CLUSTER 3

Research synthesis of 23 studies reveals emerging evidence for diversified eating disorder interventions beyond traditional psychotherapy approaches. Pharmacological treatments demonstrate notable efficacy, with lisdexamfetamine showing large effect sizes for binge eating disorder (SMD=0.93), while GLP-1 agonists and combination naltrexone/bupropion therapy show promise for weight-related outcomes. Neurostimulation interventions, particularly deep brain stimulation for treatment-refractory anorexia nervosa, demonstrate substantial effects (Hedges' g=1.13), challenging assumptions about intervention hierarchies that traditionally favor CBT as first-line treatment. However, the evidence base remains limited by predominantly female samples (85-100% across studies), complete absence of socioeconomic status reporting, and minimal racial/ethnic diversity, severely constraining generalizability. Future research priorities should address these demographic gaps while pursuing longer-duration studies to establish maintenance of treatment effects and develop individualized treatment algorithms that integrate pharmacological, neurostimulation, and psychological approaches based on patient characteristics and disorder subtypes.

SUMMARY FOR CLUSTER 4

This cluster of 19 articles reveals consistent pathophysiological patterns in eating disorders, with robust evidence for hormonal dysregulation including decreased leptin (SMD -1.62 to -3.84), elevated ghrelin (SMD 2.51), and increased cortisol across measurement methods, alongside reduced growth factors BDNF and IGF-1, elevated inflammatory markers TNF-α and IL-1β, and cardiac structural abnormalities with preserved functional capacity. These biomarker changes appear to represent adaptive responses to starvation rather than primary pathology, with genetic associations showing population-specific effects (5-HTR2A polymorphisms significant only in Caucasian populations). Critically, medical instability occurs across weight ranges, with 29-42% of non-underweight adolescents requiring hospitalization, emphasizing that rapid weight loss is more predictive of complications than absolute weight. The convergent findings across multiple large meta-analyses suggest these biological markers collectively characterize the eating disorder phenotype, though individual biomarkers lack diagnostic specificity, pointing toward the need for multi-modal clinical approaches and highlighting significant

research gaps in socioeconomic and ethnic diversity representation.

SUMMARY FOR CLUSTER 5

This cluster reveals consistently elevated eating disorder prevalence across vulnerable populations, with particularly high rates in transgender individuals (17.7%) and those with type 1 diabetes (14.4% increased risk), alongside strong bidirectional relationships between eating disorders and medical conditions including diabetes, pregnancy complications, and psychiatric comorbidities. The findings demonstrate robust associations between childhood maltreatment and eating disorder development (OR 2.37), with sexual abuse showing the strongest relationship, while medical comorbidities present complex patterns—anorexia nervosa paradoxically reduces gestational diabetes risk (OR 0.63) but increases low birth weight risk (OR 1.74). Critical research gaps emerge in the overwhelming focus on White, Western, female populations, with 83% of studies lacking adequate racial/ethnic diversity and 89% providing insufficient socioeconomic data, highlighting an urgent need for systematic screening protocols in high-risk populations and culturally responsive research approaches that address these demographic biases in future investigations.

SUMMARY FOR CLUSTER 6

Meta-analytic evidence reveals that eating disorders present with severe multi-system complications requiring comprehensive clinical attention. Sleep disturbances in anorexia nervosa (AN) include reduced total sleep time by 21 minutes and decreased sleep efficiency by 3.3% that persist after weight restoration (Burger2024), while dental complications show dramatic increases in erosion across all eating disorders (OR=15.27) with particularly severe effects in bulimia nervosa (54.4% prevalence) (Chan2024). Importantly, other specified feeding or eating disorders (OSFED) demonstrate comparable or even greater severity than full-threshold diagnoses, with atypical AN patients showing higher eating pathology than typical AN patients, challenging traditional diagnostic hierarchies (Dang2024; Johnson_Munguia2023). AN patients exhibit significantly elevated autism spectrum traits across age groups (effect sizes 0.77-0.79) with 18% scoring above clinical cutoffs (Inal_Kaleli), and show severe bone mineral density deficits across all skeletal sites (Lopes2022). These findings underscore the need for multidisciplinary treatment approaches addressing medical, dental, sleep, and neurodevelopmental factors, while highlighting critical gaps in research diversity as studies overwhelmingly represent White, female, Western populations.

SUMMARY FOR CLUSTER 7

This comprehensive cluster analysis of 14 systematic reviews reveals that eating disorders affect 1.69% of the global population with substantial geographic variation (0.22% in Asian countries vs. 1.89% in Western countries), carry severe mortality risk (standardized mortality ratio = 5.21 for anorexia nervosa), and demonstrate extensive psychiatric comorbidity including non-suicidal self-injury in 40% of patients and suicidal ideation in 51%. The research consistently shows that binge-purge subtypes (bulimia nervosa and anorexia nervosa binge-purge type) exhibit the highest rates of comorbid conditions, including substance use (alcohol 28%, smoking 30% in bulimia nervosa) and impulse control disorders (22% overall prevalence). These findings underscore the critical need for comprehensive screening protocols that assess suicide risk and comorbid conditions, culturally adapted assessment tools given the protective factors observed in non-Western populations, and integrated treatment approaches that address the complex interplay between eating pathology and co-occurring psychiatric conditions, particularly for high-risk binge-purge presentations.

SUMMARY FOR CLUSTER 8

Mental health comorbidities are pervasive across psychiatric conditions, with multiple large-scale meta-analyses demonstrating that comorbidity is the rule rather than the exception. Particularly striking patterns emerge in neurodevelopmental conditions, where very preterm birth increases ADHD risk 5-fold and autism risk 10-fold (Anderson2021), while autism itself shows extensive comorbidities including 87% prevalence of developmental coordination disorder and 37% ADHD prevalence (Micai2023). Familial transmission follows transdiagnostic patterns, with offspring of parents with mental disorders showing 1.5-3.0 times higher risk of developing any mental disorder across 37 significant transdiagnostic associations (Uher2023). The clinical impact is severe, with mental disorders reducing life expectancy by nearly 15 years globally (Chan2023), while age-of-onset data reveals that 34.6% of all mental disorders begin before age 14 and 48.4% before age 18 (Solmi2022), challenging traditional service delivery models that divide care at age 18. These findings underscore the critical need for transdiagnostic screening approaches, early intervention strategies targeting high-risk populations, and integrated care models that address the complex comorbidity burden rather than treating conditions in isolation.

SUMMARY FOR CLUSTER 9

This cluster reveals substantial comorbidity between eating disorders and addictive/compulsive behaviors, with particularly elevated rates in binge-purge subtypes. Key findings include alcohol use disorders affecting ~20% of individuals with binge eating (Bogusz2020), food addiction prevalence of 55% in clinical binge eating populations (Praxedes2022), and impulse control disorders occurring in 22% of eating disorder cases overall (Devoe2022). Social determinants significantly influence risk, with food insecurity increasing binge eating odds by 66% (Abene2023), while attachment insecurity shows large effect sizes across all eating disorder types (Jewell2023). These findings underscore the critical need for integrated screening and treatment approaches that address both eating pathology and co-occurring addictive behaviors, with particular attention to the elevated risk profiles of bulimia nervosa and binge eating disorder populations.

SUMMARY FOR CLUSTER 10

This cluster of 10 meta-analyses reveals consistent evidence for the effectiveness of diverse eating disorder treatments, with psychological interventions maintaining 45–53% abstinence rates at 12 months (Hilbert2020), exercise showing large effect sizes for BED (SMD = −0.94; Raisi2023), and weight loss interventions actually improving rather than worsening disordered eating symptoms (SMD = −1.47; Tsompanaki2025). Notably, several findings challenge traditional clinical assumptions: illness duration shows no significant association with treatment outcomes (r = .05; Radunz2020), compulsory treatment achieves similar effectiveness to voluntary treatment despite more severe presentations (Atti2021), and individuals with BED can successfully engage in weight loss interventions, though with modestly reduced weight loss and higher dropout rates compared to non-BED individuals (Forman2023). The research demonstrates significant long-term improvements across most disordered eating behaviors over 10+ years (Romano2020) and reveals that cognitive inefficiencies are similarly present in both anorexia and bulimia nervosa but not in BED (Keegan2021), supporting a more optimistic, individualized approach to treatment while highlighting the need for continued research in more diverse populations beyond the predominantly female, largely White samples that characterize current literature.

SUMMARY FOR CLUSTER 11

Research evidence across eating disorder treatment and prevention interventions reveals a consistent pattern of effectiveness for various intensity levels and delivery modalities. Low-intensity and self-guided interventions demonstrate equivalent efficacy to high-intensity therapy for eating disorder psychopathology ($g = -0.13$, 95% CI: -0.30, 0.04) while being more accessible, though with lower remission rates (Davey2023). Early treatment response emerges as a robust predictor of outcomes across all eating disorder types ($r = 0.41$, 95% CI: 0.32-0.48) (Chang2021), while intensive residential treatments show superior outcomes to other settings with large effect sizes for symptom improvement ($g = 0.97$, 95% CI: 0.85, 1.09) (deBoer2023). Prevention programs demonstrate significant effectiveness in reducing eating disorder onset by 38% (IRR = 0.62, 95% CI: 0.44, 0.87), with peer-led and single risk factor approaches showing particular promise (Harrer2020; Stice2021). These findings support stepped-care models incorporating early identification, accessible low-intensity interventions, and intensive treatment when needed, though the severe underrepresentation of males and limited racial/ethnic diversity across studies represents a critical gap requiring urgent attention in future research.

SUMMARY FOR CLUSTER 12

This systematic analysis of nine meta-analyses reveals consistent medium-to-large associations between key psychological factors and eating disorders, with self-disgust ($g = 1.90$) and eating-related shame ($r = 0.589$) showing the strongest effects, followed by social physique anxiety ($r = 0.51$) and self-criticism ($r = 0.37$), while perfectionism demonstrated more moderate but consistent associations across youth and adults ($r = 0.20$-0.33). Self-compassion emerged as a protective factor with medium negative associations ($r = -0.34$) and showed promise in interventions ($g = 0.58$), while CBT targeting perfectionism demonstrated efficacy for eating disorder symptoms ($g = 0.61$). These findings suggest that shame, disgust, and self-criticism may represent priority targets for intervention, though research is severely limited by overrepresentation of White, female, educated participants and predominantly cross-sectional designs, highlighting critical needs for longitudinal studies and diverse populations in future eating disorder research.

SUMMARY FOR CLUSTER 13

This cluster of nine systematic reviews reveals critical gaps in eating disorder care, with only 30% of individuals seeking help despite effective treatments being available. The most robust finding is that approximately two-thirds of people with eating disorders have unmet treatment needs (Ali2024), primarily due to denial of severity and perceived inability of others to help (Radunz2022). While recovery rates reach 46% overall and improve to 67% with long-term follow-up (Solmi2024), relapse remains common at 36-37% across disorders, with post-treatment BMI and pre-treatment depression being the strongest predictors (Rijk, Sala2023). The SCOFF questionnaire demonstrates good screening accuracy (84% sensitivity, 80% specificity) (Feltner2022), and evidence-based treatments like guided self-help and lisdexamfetamine for binge eating disorder show significant effectiveness. However, a major limitation across all studies is the systematic under-representation of males, racial/ethnic minorities, and diverse socioeconomic populations, highlighting an urgent need for more inclusive research to ensure findings are generalizable to all affected populations.

SUMMARY FOR CLUSTER 14

Multiple systematic reviews and meta-analyses consistently demonstrate that COVID-19 significantly worsened eating disorder symptoms and increased healthcare utilization, with 57-65% of patients experiencing symptom deterioration across studies (Haghshomar2022; Khraisat2022; Sideli2021). Güzel2023's large-scale analysis (n=4,688,559) found increased ED prevalence (OR=1.30) and moderate effect sizes for symptom severity across anorexia nervosa, bulimia nervosa, and binge eating disorder (d=0.24-0.29), while Madigan2025 reported a 54% increase in pediatric ED healthcare utilization (RR=1.54), with disproportionate impacts on adolescent girls. However, vanReekum2023's longitudinal before-after analysis found no significant objective symptom changes (MD=0.44), suggesting a discrepancy between perceived and measured deterioration. The research reveals concerning demographic gaps, with severe underrepresentation of diverse racial/ethnic groups and limited socioeconomic data across all studies, predominantly reflecting Western, female populations and highlighting the need for more inclusive research to understand pandemic impacts across diverse communities.

SUMMARY FOR CLUSTER 15

This cluster of six systematic reviews reveals that pharmacological interventions for anorexia nervosa show limited efficacy, with olanzapine emerging as the only medication with modest but significant benefits for weight gain in adults (BMI increase of 0.67 kg/m^2) but not adolescents (Han2022). Despite evidence of significant oxytocin dysregulation in both restricting and binge-eating/purging subtypes of AN (Ferreira2022), intranasal oxytocin supplementation failed to improve social cognition or food intake in AN patients, contrasting with beneficial effects observed in non-psychiatric populations (Chen2021, Hasselbalch2020). Traditional psychotropic medications, including antidepressants and other antipsychotics, demonstrated no significant effects on weight recovery or psychiatric symptoms (Cassioli2020), though dropout rates for second-generation antipsychotics remain acceptable at 28%, primarily driven by personal choice rather than adverse effects (Kan2020). These convergent findings underscore the limited role of pharmacological approaches in AN treatment and highlight the critical need for larger, well-designed trials while reinforcing psychological interventions as the primary evidence-based treatment modality.

SUMMARY FOR CLUSTER 16

This cluster establishes ARFID as a clinically significant eating disorder with a prevalence of 4.51% (95% CI: 0.7–10.68%) using quality-effects meta-analysis, representing a 15-fold higher rate than previously estimated (Nicholls-Clow2024). Despite similar BMI z-scores to anorexia nervosa patients, ARFID patients demonstrate distinct physiological profiles including significantly higher heart rates (WMD = 12.82 bpm, 95% CI: 7.58–18.12) and physical health complications across the entire weight spectrum (James2024). The strong comorbidity with autism spectrum disorder, with autism prevalence of 16.27% (95% CI: 8.64%–28.53%) in ARFID populations compared to ~1% in the general population (Sader2025), highlights the need for cross-condition screening protocols. These findings collectively indicate that ARFID requires specialized clinical assessment and monitoring approaches that differ from traditional eating disorder protocols, particularly given its unique demographic profile showing male predominance or equal gender distribution rather than the female predominance typical of other eating disorders.
